# Rational Design of an Electronically Gated Dicyano-BODIPY Platform for Reversible-Covalent Imaging of Methylglyoxal

**DOI:** 10.1021/jacsau.6c00566

**Published:** 2026-06-15

**Authors:** John M. Talbott, Samrat Kundu, Brandon Li, Leslie Hassanein, Prakashkumar Dobariya, David Weinshenker, Swati S. More, Monika Raj

**Affiliations:** † Department of Chemistry, 1371 Emory University, Atlanta, Georgia 30322, United States; ‡ Department of Human Genetics, Emory University School of Medicine, Atlanta, Georgia 30322, United States; § Center for Drug Design, College of Pharmacy, University of Minnesota, Minneapolis, Minnesota 55455, United States

**Keywords:** methylglyoxal, BODIPY, photoinduced electron transfer (PET), live-cell imaging, tissue imaging

## Abstract

Reactive α-dicarbonyls, such as methylglyoxal (MGO), are critical biomarkers of carbonyl stress, yet their real-time monitoring is stifled by a selectivity-biocompatibility paradox. Existing probes either suffer from aldehyde promiscuity, failing to distinguish dicarbonyls from the global lipid peroxidation background, or rely on high-energy, cytotoxic excitation that precludes longitudinal study. Herein, we report the rational design of a dicyano-BODIPY platform engineered to resolve this tension through a precision-tuned acceptor-photoinduced electron transfer (a-PET) mechanism. By employing density functional theory (DFT) as a predictive blueprint, we strategically depressed the BODIPY core HOMO to −6.63 eV, establishing a specific energetic gradient that enforces a robust “off” state until triggered by reversible covalent capture of α-dicarbonyls. This electronically gated design enables longitudinal, visible-light imaging of bidirectional MGO flux in living cells, a feat inaccessible to current irreversible sensors. We further demonstrate the platform’s high-fidelity performance in complex biological matrices by mapping dose-responsive MGO burden in murine brain tissue following controlled intracranial perturbation, providing a vital tool for interrogating the role of glyoxal stress in tissue-level pathologies. This work provides a generalizable electronic framework for the development of reversible-covalent sensors capable of monitoring metabolic dynamics in intact biological environments

## Graphical abstract

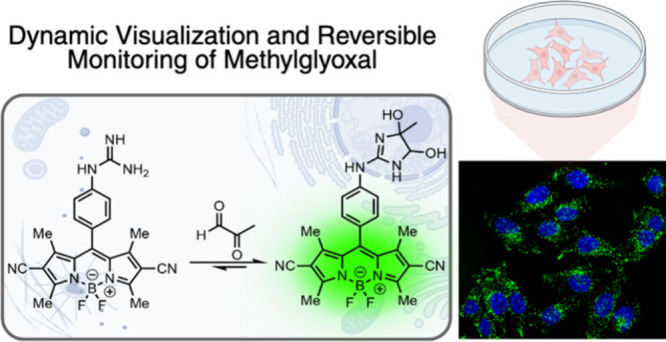

The central challenge in glyoxal biology is not merely detection, but differentiation within a crowded electrophilic landscape.^1,2^ Reactive α-dicarbonyls such as methylglyoxal (MGO) drive the formation of advanced glycation end-products (AGEs) implicated in neurodegeneration and metabolic disease,^3–14^ yet in cells they coexist with an overwhelming background of abundant monoaldehydes. Conventional analytical approaches typically require cell or tissue lysis followed by chromatography- or antibody-based readouts.^15,16^ While powerful, these workflows are inherently destructive and can obscure compartment-specific or time-dependent changes in α-dicarbonyl burden.^17,18^ Aldehyde-sensing motifs, most prominently phenylenediamines, are intrinsically promiscuous and often yield indistinguishable responses to α-dicarbonyls and common aliphatic aldehydes.^19–23^ Despite numerous literature reports of phenylenediamine systems for selective glyoxal detection,^19,20^ we have robustly established the rapid reaction of phenylenediamines with aliphatic aldehydes, yielding stable, fluorescent benzimidazole products (Figure 1a).^21,23^ Compounding this limitation, many probe designs rely on irreversible trapping, producing a cumulative record of exposure rather than a dynamic readout of real-time metabolic burden. A probe that is both chemoselective and reversible would therefore enable time-resolved imaging of dicarbonyl flux in living systems. Guanidino-based recognition offers substantially improved selectivity for α-dicarbonyls; however, its broad deployment has been constrained by bioincompatibility. Reported guanidine-based probes frequently require acidic conditions to access reactive guanidinium states or high-energy UV excitation that can induce cytotoxicity, limiting longitudinal studies in living systems (Figure 1b) (Supplementary Figure S1).^24,25^

Herein, we sought to move beyond empirical optimization toward a computationally guided electronic architecture. We reasoned that the performance of a guanidine-BODIPY probe depends critically on engineering a robust “off” state (Figure 1c). Initial studies indicated that standard BODIPY scaffolds are electronically mismatched: their HOMO energies are insufficiently aligned to enable efficient photoinduced electron transfer (PET) quenching by a phenylguanidine moiety. We hypothesized that this mismatch could be resolved by electronically engineering the fluorophore core (Figure 1c). Accordingly, we performed a systematic DFT-guided screen to match the energy levels of a phenylguanidine quencher to a series of substituted BODIPY reporters.

**Figure 1:**
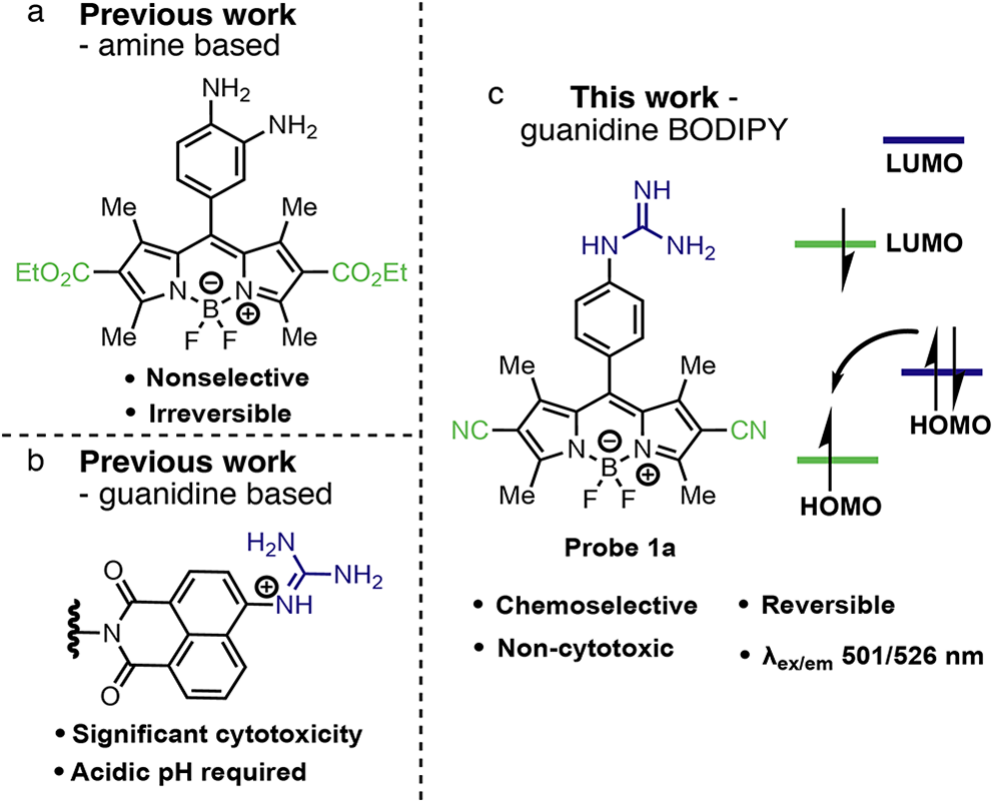
(a) Earlier generation BODIPY probes utilizing *o*-phenylenediamine motifs suffer from inherent aldehyde promiscuity and irreversible trapping mechanisms. These probes react indiscriminately with the global cellular aldehydome including ubiquitous monoaldehydes to produce cumulative signals that obscure specific dicarbonyl flux. (b) Previous guanidine-based recognition offers superior selectivity toward methylglyoxal but are severely limited by bioincompatibility. These systems often exhibit significant cytotoxicity, require acidic operational environments, or rely on high-energy, blue-shifted excitation that induces substantial cellular photodamage. (c) This work: The dicyano-BODIPY platform (**1a**), a precision-engineered dicyano-BODIPY. The probe is reversible, noncytotoxic, and selective toward methyl glyoxal while utilizing cellularly compatibly wavelengths enabling real-time monitoring of MGO dynamics in intact biological systems.

By appending dicyano electron-withdrawing groups, we lowered the BODIPY HOMO to ≈−6.63 eV, enabling an efficient PET-gated “off” state that is specifically and reversibly relieved upon α-dicarbonyl capture (Figure 1c). Because guanidine-MGO binding proceeds via a reversible covalent adduct, fluorescence reports the current dicarbonyl burden rather than historical exposure. As a result, the probe supports longitudinal live-cell imaging and enables mapping of pathological MGO burden in murine brain tissue, thus linking molecular recognition to organ-level metabolic reporting.

## Results and Discussion

### Designing a Dicarbonyl-Specific Fluorescent Probe

Our objective was to engineer a reversible, α-dicarbonyl-gated off-to-on response via a guanidine recognition unit. We targeted a design in which the free probe is quenched by acceptor-photoinduced electron transfer (a-PET), and α-dicarbonyl capture shifts orbital energetics to suppress PET and restore emission.^26^ Achieving this behavior required deliberate control of the relative HOMO energies of the donor (phenylguanidine) and the BODIPY core.

DFT calculations showed that direct installation of phenylguanidine onto our previously used diester-BODIPY scaffold was electronically mismatched for a-PET gating. The phenylguanidine HOMO (≈−6.29 eV) lies below the BODIPY HOMO (≈−5.99 eV), eliminating a favorable energetic gradient for donor-to-acceptor electron transfer. In this configuration, the probe is predicted to be insufficiently quenched in the unbound state, undermining a true turn-on response. Rather than proceeding by empirical synthesis and screening, we reframed the problem as an orbital alignment challenge.

We evaluated two computational strategies to restore PET quenching: (i) raising the phenylguanidine HOMO via electron-donating substitution and (ii) lowering the BODIPY HOMO via electron-withdrawing substitution. We first attempted to “push” the phenylguanidine HOMO upward by installing subsitituent groups around the aryl ring (Figure 2a and Supplementary Figure S2). However, the calculated HOMO energies either decreased or remained relatively equivalent (e.g., from ≈−6.29 to ≈−6.31 eV for dihydroxyl subsitution) and did not produce the energetic offset needed to enable robust a-PET quenching.

**Figure 2:**
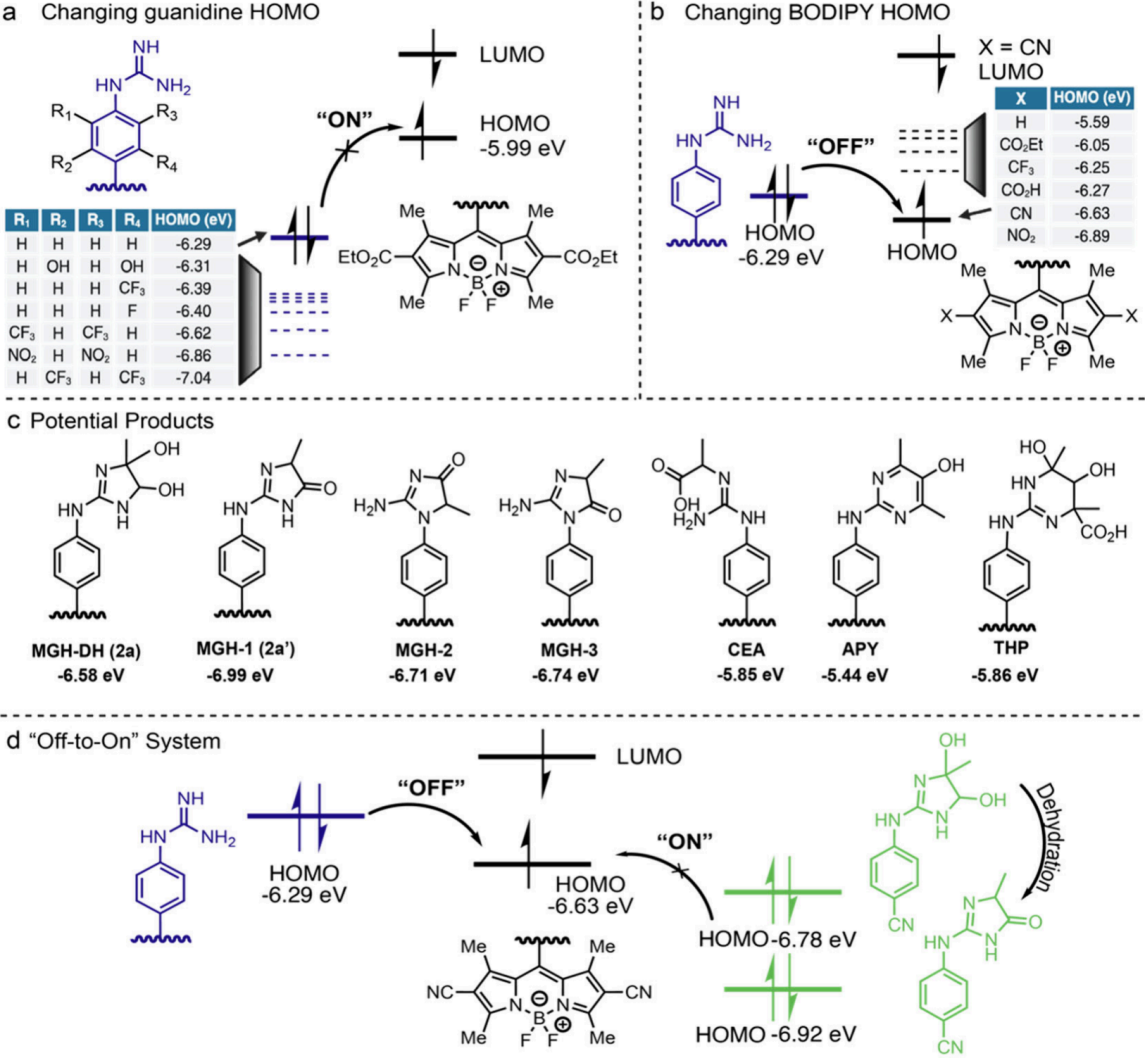
Density functional theory (DFT) calculations for probe optimization. (a) Phenyl rings with varying substituents demonstrated that standard scaffolds fail to facilitate a-PET quenching due to the relatively low HOMO energy of the quencher (−6.29 eV) compared to standard diester-BODIPY cores. (b) Systematic screening of the BODIPY core with diverse electron-withdrawing groups (EWGs). Only dicyano and dinitro substitution successfully depressed the BODIPY HOMO to a critical threshold (−6.63 eV), establishing the specific energetic gradient required for a robust “off” state. (c) Evaluation of the seven theoretically possible covalent adducts between phenylguanidine and MGO. (d) Comprehensive schematic of the precision-tuned a-PET system.

Realizing the phenylguanidine donor was electronically stubborn, we inverted our strategy to focus on lowering the BODIPY acceptor HOMO to restore the energetic gradient required for a-PET quenching. We therefore carried out a systematic DFT screen of BODIPY cores bearing electron-withdrawing substituents. Carboxylic acids (CO_2_H) and esters (CO_2_Et) produced only modest stabilization (BODIPY HOMO ≈ −6.05 to −6.27 eV), and even trifluoromethyl substitution (CF_3_; ≈−6.25 eV) failed to create a sufficient offset relative to phenylguanidine (≈−6.29 eV). In contrast, dicyano substitution produced a pronounced depression, lowering the BODIPY HOMO to ≈−6.63 eV (Figure 2b and Supplementary Figure S1). This inversion of orbital ordering, placing the phenylguanidine HOMO above the BODIPY HOMO, established a robust driving force for a-PET in the free probe, enforcing a strongly quenched “off” state. Notably, NO_2_ substitution also yielded potentially favorable energies (HOMO ≈ 6.89 eV), suggesting additional design space for future variants with parallel donor tuning. Collectively, these calculations pinpointed the dicyano-BODIPY core as the minimal electronic modification that converts the guanidine handle from a poor quencher into an effective PET gate, setting the stage for a probe that remains silent until α-dicarbonyl capture.

With the off-state scaffold established, we next had to evaluate if the probe would become fluorescent upon MGO binding. Guanidine-MGO chemistry can, in principle, furnish up to seven distinct adduct classes including open-chain addition products, hydrated intermediates, dehydrated cyclic species, and rearranged isomers,^8,11^ so a useful probe must react selectively and produce a fluorescent signal from the adduct(s) that actually dominates under relevant physiological conditions. Most candidate adducts, including argpyrimidine (APY) and THP-type structures, were predicted to generate HOMO energies (≈−5.44 to −5.86 eV) that preserve the energetic driving force for a-PET and therefore remain effectively “off“ (Figure 2c and Supplementary Figure S2). In contrast, formation of the dihydroxyimidazolidine adduct (MGH-DH, **2a**) and its dehydrated derivative (**MGH-1, 2a**') were predicted to shift the frontier orbital alignment (≈−6.58 to −6.99 eV) that could eliminate a-PET quenching and restore fluorescence.

Notably, in an initial simplified model the calculated HOMO for MGH-DH was −6.58 eV, which appeared insufficient to fully disrupt a-PET and produce a robust turn-on response (Figure 2c and Figure S2). We reasoned, however, that this value likely overestimated the true HOMO energy level because it did not fully capture the strong electron-withdrawing influence of the dicyano-BODIPY core. To approximate this effect while keeping the calculations tractable, we introduced a para-cyano substituent as an electronic surrogate and recalculated the adduct, which lowered the predicted HOMO for MGH-DH to ≈−6.78 eV (Figure 2d and Supplementary Figure S2), aligning with the “on-state” regime identified above. Encouragingly, the dihydroxyimidazolidine is reported to be the kinetically favored and predominant species in aqueous solution near physiological pH, with alternative adducts observed only in trace amounts,^8,11,23,24^ supporting the expectation that probe activation will track the major dicarbonyl-capture event. Taken together, these calculations suggested that the dicyano-BODIPY/guanidine architecture should function as a true off-to-on system triggered by α-dicarbonyl capture (Figure 2d and Supplementary Figure S2), motivating synthesis of probe **1a** and experimental validation of its response.

### Translating Electronic Predictions into Synthesis

With the dicyano-BODIPY scaffold identified by DFT capable of enforcing an a-PET-quenched “off” state while remaining electronically poised for turn-on upon α-dicarbonyl capture, we next developed a modular synthesis to access probe **1a** (Figure 3 and Supplementary Figure S3). The route was designed to enable late-stage installation of the guanidine recognition unit, minimizing functional group incompatibilities while preserving the electronically tuned core.^27^ Briefly, cyano-dimethylpyrrole **5** was prepared in 50% yield over four steps and condensed (TFA-catalyzed) with 4-nitrobenzaldehyde to afford the nitro-substituted precursor **6**. An iron-mediated reduction then furnished aniline **7** in 86% yield (over two steps), providing a convenient handle for incorporation of the protected guanidine moiety. Installation of the guanidine core proceeded efficiently using HgCl_2_ and di-Boc protected *S*-methylisothiosemicarbazide to give intermediate **8** (91% yield). In the final step, oxidation with DDQ followed by BF_2_ complexation delivered the target dicyano-BODIPY probe **1a**, with BF_2_ installation simultaneously promoting Boc deprotection to reveal the active guanidinium recognition element (Figure 3 and Supplementary Figure S3).^28^ This sequence provided straightforward access to **1a** for photophysical characterization and biological validation of the computation-guided design. Probe **1a** was stable at −80 °C for >6 months with no noticeable degradation.

**Figure 3:**
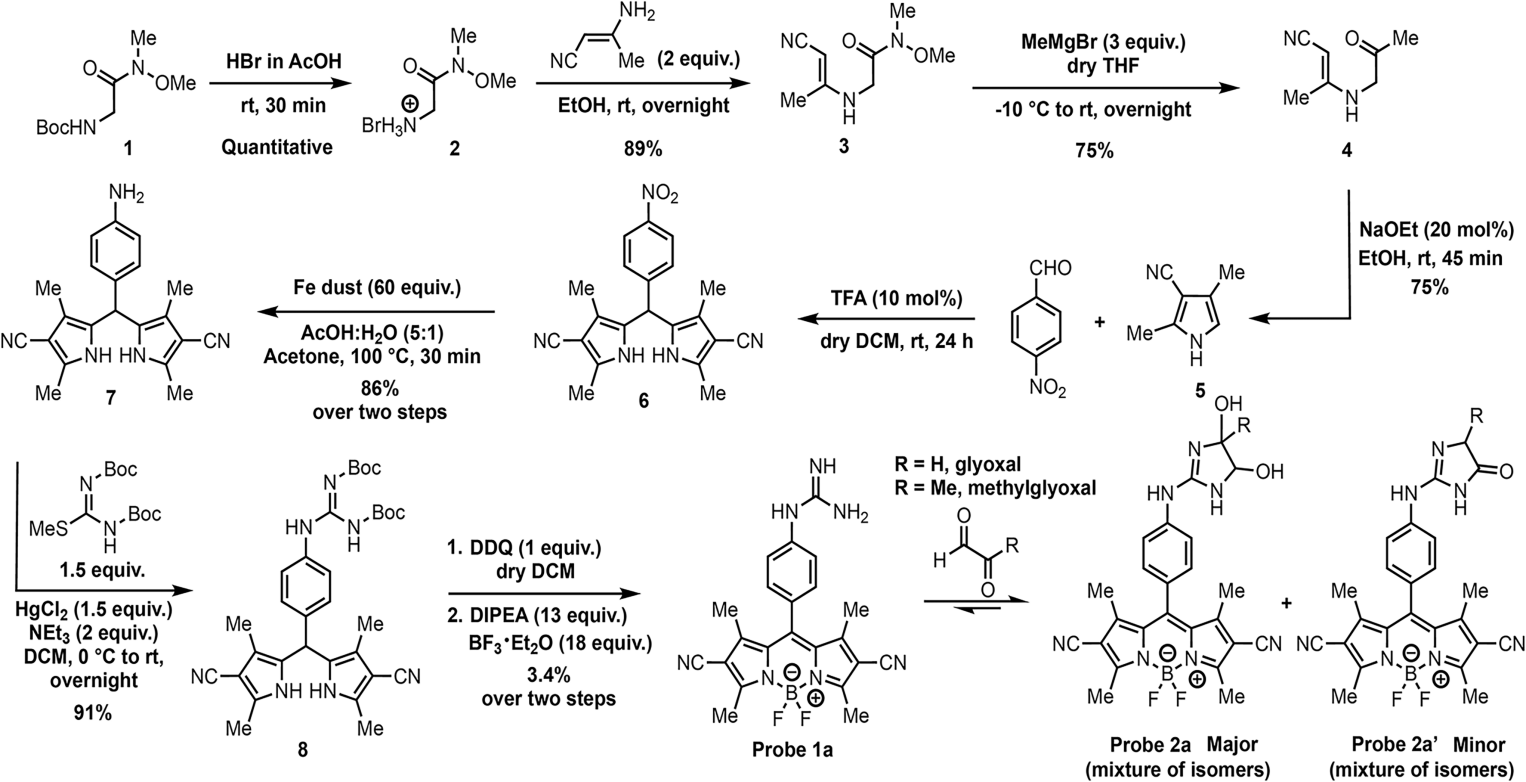
Synthesis of probe **1a** and reaction with glyoxal species yielding fluorescent probe **2a/2a′**.

### Experimental Validation of Electronic Gating

Exposure of probe **1a** with MGO for 2 h produced the dihydroxyimidazolidine adduct (**2a**) as the major product and its dehydrated derivative (**2a′**) as a minor product as characterized by HPLC and HRMS (Figure 4a and Supplementary Figure S3).

**Figure 4:**
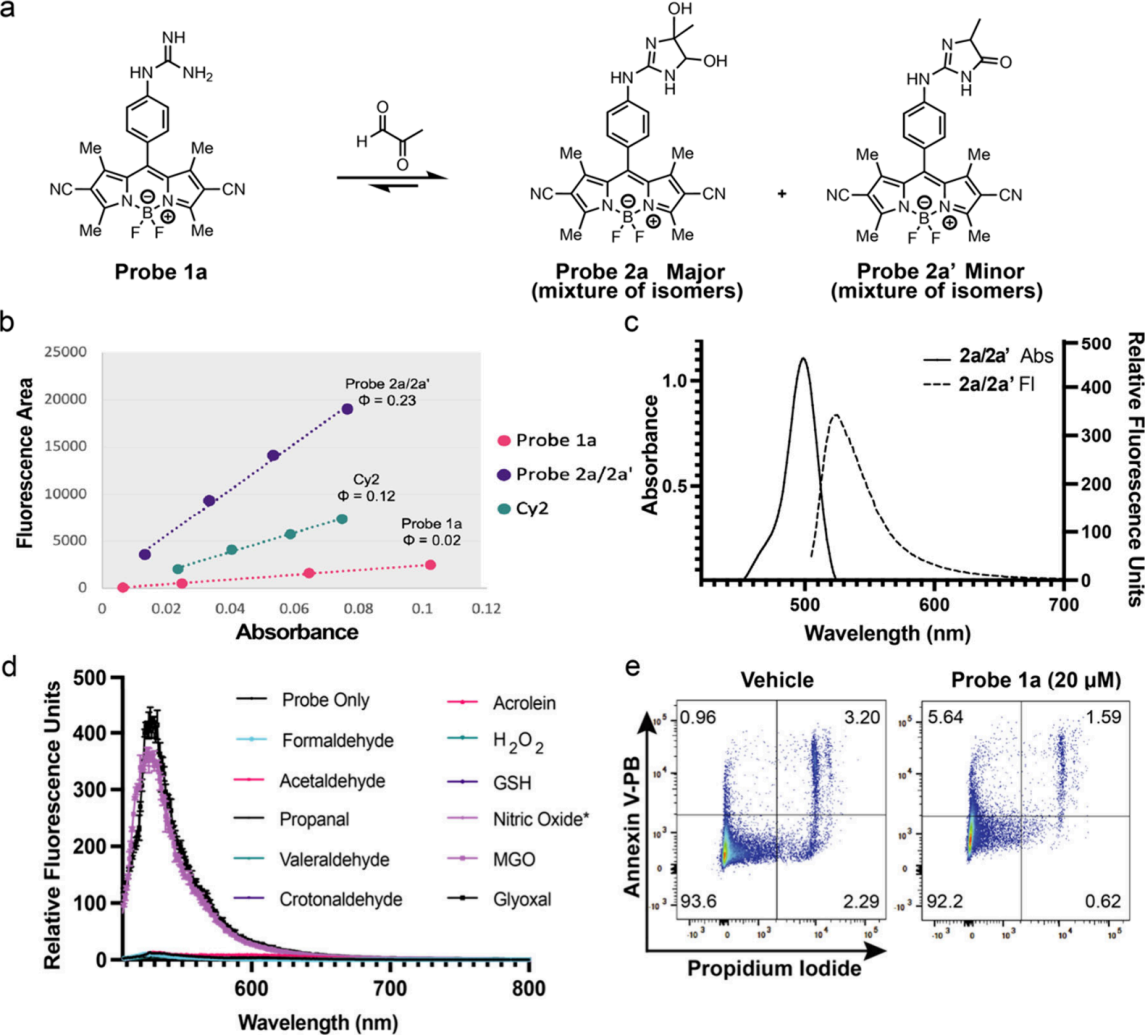
Characteristics of probe **1a**. (a) Reaction of probe **1a** with dicarbonyl species generates hydrate product **2a** (major) and eliminated product **2a′** (minor). (b) Quantum yield of probe **1a** (∼0.02) and its activated adducts **2a/2a′** (∼0.23) relative to Cy2 (∼0.12), demonstrating a high-efficiency turn-on response. Data displayed is an average of three replicates. (c) Absorbance and emission spectra of the activated species in PBS buffer (10 mM, pH 7.4). Excitation λ = 501 nm, emission λ = 526 nm. (d) Fluorescence response of **1a** challenged with 100 equiv. of representative monoaldehydes and metabolic interferents. Significant activation is strictly isolated to (MGO/GO), highlighting the specificity of the engineered guanidine-PET gate. NO was generated in situ using *S*-nitroso-*N*-acetylpenicillamine (SNAP). Error bars represent mean ± standard deviation. (e) Annexin V/PI flow cytometry of HeLa cells following 24-h incubation with **1a** (20 μM, 24 h). No significant loss in viability was observed relative to vehicle controls, establishing the probe’s suitability for longitudinal metabolic imaging. Data was repeated in triplicate.

Consistent with the computation-guided design, probe **1a** is weakly emissive in buffered aqueous media (Φ ≈ 0.02, Figure 4b and Supplementary Figure S4), supporting effective quenching in the unbound state and validating that dicyano substitution provides the energetic requirements for an a-PET-gated “off” state. Upon exposure to α-dicarbonyl analytes, **1a** exhibited a pronounced fluorescence activation, increasing the quantum yield to Φ ≈ 0.23 (≈11-fold enhancement; Figure 4b and Figure S4), consistent with disruption of the a-PET quenching pathway predicted by DFT.

The activated adducts displayed excitation/emission maxima at 501/526 nm in PBS buffer (10 mM, pH 7.4) (Figure 4c and Supplementary Figure S5), placing the signal in a visible window advantageous for biological imaging by reducing autofluorescence interference and minimizing photodamage relative to UV-excited guanidine-based platforms.

To assess chemoselectivity, probe **1a** was challenged with representative monoaldehydes (formaldehyde, acetaldehyde, propanal, valeraldehyde, crotonaldehyde, and acrolein), α-dicarbonyls (MGO and GO), and common biological interferents (H_2_O_2_, glutathione, and nitric oxide (NO)) under matched conditions (100 equiv.; Figure 4d and Supplementary Figure S5).

In striking contrast to phenylenediamine-based probes, which rapidly turn on broadly across the cellular aldehydome,^21,23^ **1a** remained essentially silent toward monoaldehydes and interferents, while showing strong activation only in the presence of MGO and GO. To assess selectivity, a competition study was conducted using equimolar quantities of MGO and potential interfering species (100 equiv. each), including monoaldehydes and biologically relevant interferants. Fluorescence responses remained consistent across all conditions, confirming that sensor reactivity is uncompromised in complex chemical environments (Supplementary Figure S5). Collectively, these data demonstrate that the guanidine recognition motif, in concert with electronic gating, effectively discriminates the 1,2-dicarbonyl signature from the broader reactive carbonyl background, enabling selective, visible-light turn-on detection under biocompatible conditions.

To evaluate reversibility, probe **1a** was subjected to alternating additions of MGO and aminoguanidine, a well-established MGO scavenger,^24,25^ with fluorescence intensity monitored across successive cycles. A pronounced and reproducible response was observed with each addition, confirming that the sensing event is reversible and consistent over multiple cycles as analyzed by MS spectrometry and fluorometer (Supplementary Figure S5).

The reaction kinetics of sensor **1a** with MGO were subsequently characterized under pseudo-first-order conditions. A rate constant of *k*_obs_ = 0.00145 s^–1^ was determined (*R* ^2^ = 0.95, *n* = 3), with an initial lag phase of approximately 15 min attributable to the two-step condensation–cyclization mechanism, and near-complete equilibration (∼95%) achieved within approximately 50 min. To ensure full equilibration and a stable fluorescence readout in cellular contexts, a 1.5-h incubation period was adopted for all subsequent cell-based assays (Supplementary Figure S5).

### Biocompatibility and Subcellular Accessibility in Living Systems

To establish operational compatibility for longitudinal imaging, we evaluated the cytotoxicity of probe **1a** under conditions used for live-cell studies. HeLa cells were incubated with probe **1a** (20 μM, 24 h) and assessed by flow cytometry using Annexin V/PI staining. Under these conditions, probe **1a** produced no significant loss in viability, relative to vehicle controls, supporting its suitability for live-cell studies over day-scale time windows (Figure 4e and Supplementary Figure S6).

Because α-dicarbonyl stress is generated and buffered across multiple intracellular environments, we next examined whether **1a** accesses relevant subcellular compartments.

Co-localization with organelle trackers showed that **1a** is broadly distributed throughout the cytoplasm, with notable partial localization to mitochondria and lysosomes, while nuclear signal remained low (Supplementary Figure S7). This distribution is advantageous for glyoxal biology as mitochondria and lysosomes represent metabolically active and degradative hubs where carbonyl stress and downstream damage can be amplified. Furthermore, minimal nuclear background helps ensure that readouts predominantly reflect cytosolic and organelle-associated dicarbonyl dynamics. Together, the low cytotoxicity and broad intracellular accessibility indicate that **1a** can report dicarbonyl fluctuations across cellular compartments without requiring targeted delivery, enabling practical longitudinal measurements in living systems.

### Live-Cell Compatibility and Imaging of α-Dicarbonyl Burden

To define the operational sensitivity of **1a** in living cells, HeLa cells were pretreated with exogenous MGO (10–250 μM, 30 min), followed by incubation with probe **1a** (20 μM) for 1.5 h prior to confocal imaging. Under these conditions, fluorescence increased dose-dependently with MGO concentration (Figure 5a and Supplementary Figure S8). Quantification of normalized pixel intensity relative to vehicle-treated controls (probe **1a** only) showed statistically significant enhancements across the tested range, with an empirical detection threshold of ∼ 25 μM under these imaging conditions (Figure 5b and Supplementary Figure S8). Having established dose responsiveness, we next asked whether probe **1a** can report bidirectional changes in endogenous α-dicarbonyl burden driven by pharmacological modulation of detoxification pathways.

**Figure 5:**
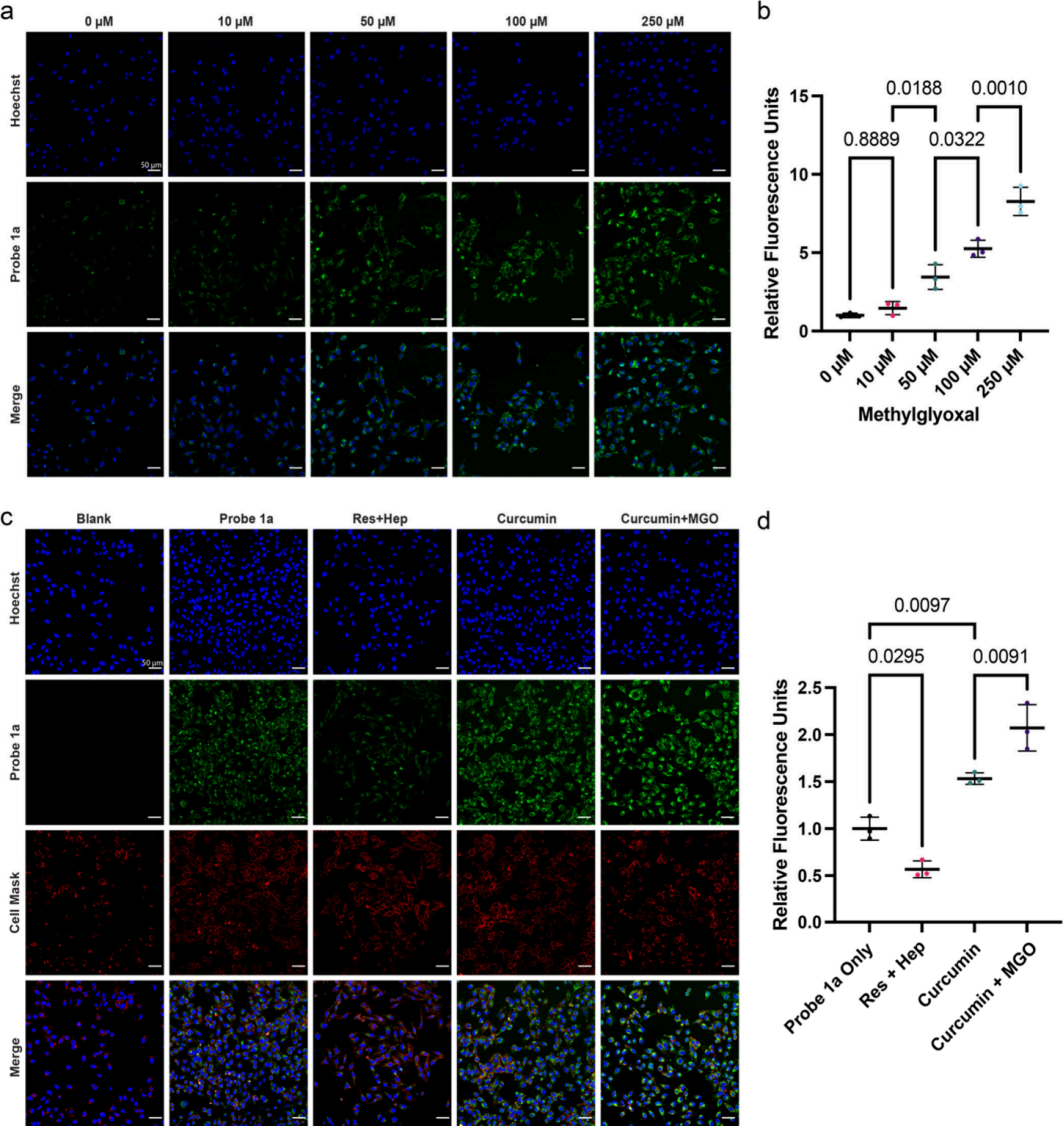
Live cell imaging of probe **1a**. (a) Live cell imaging of HeLa cells with probe **1a** (20 μM) with varying concentrations of MGO (0–250 μM) showing dose dependent increase in the fluorescence. (b) Quantification relative pixel intensity for each dosage. (c) Live cell imaging of HeLa cells with probe **1a** (20 μM) alone or with various endogenous modulators of glyoxalase-dependent MGO detoxification: treatment with glyoxalase activators, Res + Hep (resveratrol (10 μM) and hesperetin (50 μM)), resulted in decreased fluorescence; treatment with glyoxalase inhibitor, curcumin (10 μM), or curcumin (10 μM) and MGO (50 μM) resulted in increased fluorescence. (d) Quantification of relative pixel intensity. Errors bars represent mean ± standard deviation. Statistical significance determined by one-way ANOVA, *p*-values shown on graph. All experiments were performed in triplicate.

Because glyoxalase activity is a major determinant of intracellular MGO clearance, we applied complementary perturbations expected to decrease or increase cellular dicarbonyl burden (Figure 5c and Supplementary Figure S9). Pretreatment with resveratrol + hesperetin (Res 10 μM; Hep 50 μM; 24 h), a reported glyoxalase-activating regimen,^29–31^ produced a significant decrease in probe activation relative to vehicle controls, consistent with reduced intracellular MGO burden (Figure 5d and Supplementary Figure S9). Conversely, treatment with the GLO1 inhibitor curcumin^32,33^ (10 μM; 4 h) led to a significant increase in fluorescence, consistent with elevated intracellular MGO burden under impaired detoxification. As a positive-control condition, cotreatment with curcumin and exogenous MGO (50 μM) produced the highest fluorescence across conditions, consistent with additive effects of detoxification inhibition and increased MGO load via reduced clearance capacity. Additionally, we verified that probe **1a** does not react with the keto/enol functionalities in resveratrol, hesperetin, and curcumin via HPLC, as well as verifying that curcumin is not fluorescently active at the 501 nm excitation utilized for probe **1a** in physiologically relevant conditions (PBS buffer, pH 7.4), matching literature reports (Supplementary Figure S10).^34–37^ To determine the impact of curcumin on the probe uptake inside cells, HeLa cells were dosed with either MGO or MGO with curcumin. Maximum MGO dosage (250 μM) was utilized to verify that no additional fluorescent signal from curcumin inhibition of GLO1 would impact the overall fluorescent intensity. Confocal microscopy and quantitative analysis revealed no change in fluorescence between the two treatments, indicating that curcumin did not impact probe uptake (Supplementary Figure S10). Collectively, these results demonstrate that probe **1a** reports both increases and decreases in cellular MGO burden under live-cell imaging conditions, supporting its use as a reversible reporter of MGO stress dynamics.

### Longitudinal Live-Cell Reporting of α-Dicarbonyl Dynamics

A key advantage of a reversible-covalent reporter is the ability to track accumulation as well as clearance, visualizing the rise-and-fall of metabolite burden in intact cells rather than a singular time point. We therefore asked whether **1a** could report time-dependent changes in intracellular α-dicarbonyl load under conditions where detoxification capacity is experimentally tunable (Figure 6a and Figure S11). HeLa cells were pretreated with resveratrol + hesperetin (Res 10 μM; Hep 50 μM; 24 h) to enhance glyoxalase-dependent clearance, then incubated with probe **1a**. Consistent with reduced basal dicarbonyl burden, these cells displayed low fluorescence prior to challenge. Upon addition of an exogenous MGO pulse (100 μM), fluorescence increased rapidly and then decayed over ∼12 h, consistent with cellular clearance of the added dicarbonyl load (Figure 6b).

**Figure 6:**
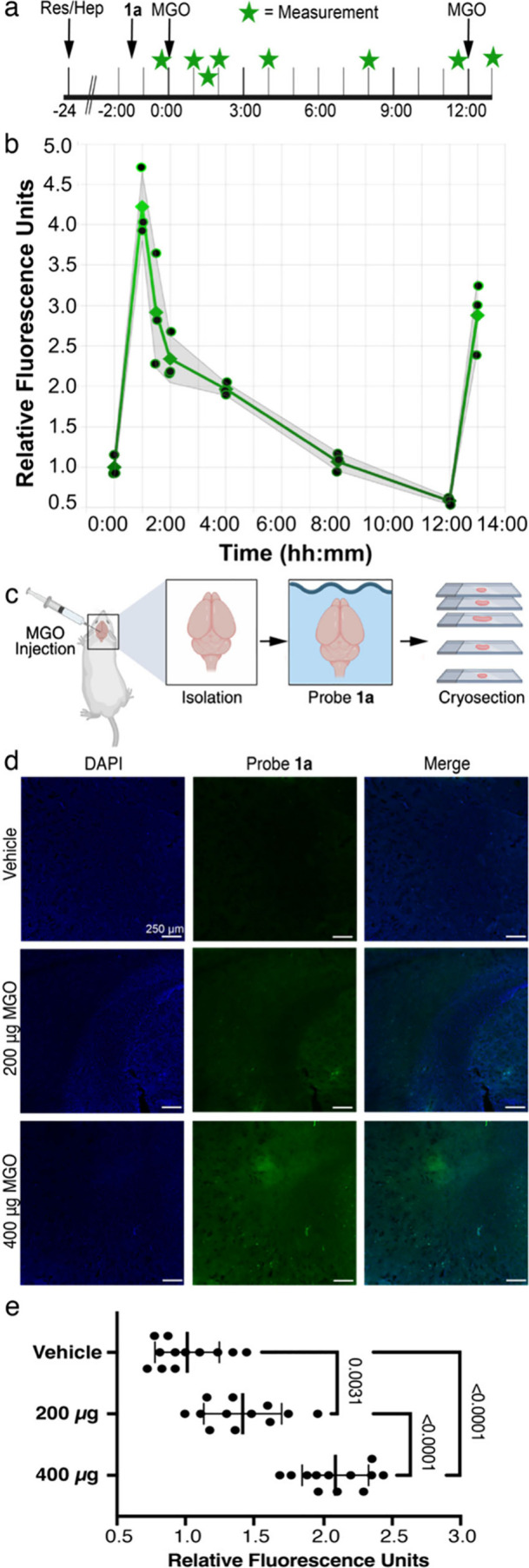
Biological applications of probe **1a**. (a,b) Time-course monitoring of bidirectional MGO dynamics in HeLa cells. Cells pretreated with glyoxalase activators (Res+Hep) followed by addition of MGO exhibited rapid peak fluorescence followed by an ∼12-h decay as the endogenous detoxification system removes MGO burden. HeLa cells were dosed with Res + Hep (resveratrol (10 μM) and hesperetin (50 μM)) for *t* = −24 h. Probe **1a** (20 μM) was added at *t* = −1.5 h. MGO (100 μM) was added at *t* = 0 h and *t* = 12 h. Relative fluorescence units over the span of 12 h. Black dots represent individual data point, diamonds represent the mean, shaded region represents the standard deviation. Repeated three times with biological replicates. (c) Schematic representation of the intracranial perturbation and ex vivo brain tissue labeling workflow. (d) Representative confocal images of murine brain sections following stereotactic injection of saline or MGO (200 μg or 400 μg for 1 h prior to sacrifice). Brains were flash frozen, then treated with probe **1a** for 24 h. Brains were fixed, cryosectioned, and counterstained with DAPI. Scale bar = 250 μm. (e) Normalized pixel intensity ROIs (two ROI per tissue slice image, two slices per mouse, from three different mice; *n* = 12). ROI quantification demonstrates a robust, dose-responsive signal enhancement in the complex neural matrix. Error bars represent mean ± standard deviation. Statistical significance determined by one-way ANOVA (*n* = 12), *p* values shown on graph.

Crucially, a second MGO pulse at the 12 h produced a renewed fluorescence increase, demonstrating that the probe remains responsive over repeated perturbation–recovery cycles. In this way, **1a** functions as a live readout of MGO flux, enabling repeated monitoring of dicarbonyl load and clearance dynamics under physiological conditions, capabilities that are difficult to achieve with irreversible trapping chemistries.

### Ex Vivo Brain Tissue Labeling

Encouraged by the live-cell performance of **1a**, we next asked whether the probe could bridge molecular sensing to a tissue context by reporting α-dicarbonyl burden in the murine brain, where MGO is a key precursor to harmful AGEs implicated in oxidative stress, inflammation, and accelerated cognitive decline.^14,38–40^ A probe that remains selective in this high-background matrix would be valuable for mapping carbonyl stress in neural tissue and for future efforts toward biomarker-relevant workflows. We sought to apply our probe on ex vivo brains that had been exposed to MGO in vivo. Live mice received stereotactic injection of MGO (200 μg or 400 μg) or vehicle (saline) into the prefrontal cortex and were sacrificed 1 h later. Brains were harvested and incubated with probe **1a** ex vivo in saline for 24 h, followed by PFA fixation, sucrose cryoprotection, cryosectioning, and DAPI counterstaining (Figure 6c and Supplementary Figure S12). Confocal imaging revealed a robust fluorescence signal in MGO-treated samples relative to vehicle controls (Figure 6d and Supplementary Figure S12). Region-of-interest quantification showed a statistically significant, dose-dependent increase in fluorescence with increasing MGO dose (two ROIs per image, two sections per mouse, three mice; *n* = 12; Figure 6e). Notably, the visible excitation/emission window of the activated probe (≈501/526 nm) supported usable signal-to-noise in brain sections, where autofluorescence and optical scattering can complicate fluorescence measurements.

Together, these results provide functional validation that the computation-guided, electronically gated design of **1a** remains operational in an intact organ matrix. While these experiments use a controlled perturbation rather than a disease model, they establish a practical foundation for applying **1a** to interrogate the contribution of dicarbonyl stress to neurodegenerative pathology and, more broadly, to explore α-dicarbonyl burden as a candidate readout in tissue-based biomarker studies.

## Conclusion

We have developed a computation-guided fluorescent platform that transforms α-dicarbonyl capture into an electronically gated emission switch. By deliberately engineering frontier orbital alignment between a phenylguanidine recognition element and a dicyano-BODIPY core, we enforced a quenched ground state and selective fluorescence restoration upon reversible dihydroxyimidazolidine formation.

Probe **1a** operates under visible-light excitation, displays high chemoselectivity for α-dicarbonyls over monoaldehydes, and supports longitudinal imaging of bidirectional MGO flux in live cells. Extension to murine brain tissue demonstrates that electronically programmed reversible sensing can function in complex biological matrices.

More broadly, this work establishes orbital engineering as a generalizable strategy for designing reversible-covalent fluorescent reporters whose selectivity is governed by chemical reactivity and electronic alignment. We anticipate that this framework will enable dynamic imaging of other transient electrophilic species in living systems.

## Supplementary Material



## Data Availability

The data underlying this study are available in the published article and its Supporting Information.

## Notes

General experimental procedures, computational calculations, and characterization details including HPLC, HRMS, and ^1^H and ^13^C NMR spectra of all compounds (PDF)
