# Supplementary material for: Rational Design of an Electronically Gated Dicyano-BODIPY Platform for Reversible-Covalent Imaging of Methylglyoxal
Source: JACS Au. 2026 Jun 15;6(7):4039–47. doi: 10.1021/jacsau.6c00566 (PMC13417195; doi:10.1021/jacsau.6c00566)

Supporting Information

Rational Design of an Electronically Gated dicyano-BODIPY Platform for Reversible-Covalent Imaging of Methylglyoxal

John M. Talbott,[‡a] Samrat Kundu,[‡a] Brandon Li,[a] Leslie Hassanein,[b] Prakashkumar Dobariya,[c] David Weinshenker,[b] Swati S. More,[c] Monika Raj[a]*

[a]Department of Chemistry, Emory University, Atlanta, GA, 30322, United States.
[b]Department of Human Genetics, Emory University School of Medicine, Atlanta, GA, 30322, United States.
[c]Center for Drug Design, College of Pharmacy, University of Minnesota, Minneapolis, MN, 55455, United States.
*Corresponding author: monika.raj@emory.edu
‡ These authors contributed equally

Table of Contents **Page**

**I.**	General	S2
**II.**	Materials	S2
**III.**	Analytical Methods	S3
**IV.**	Cell Culture Technique	S3
**V.**	Supplementary Figure S1: Reported MGO Probes	S4-S7
**VI.**	Supplementary Figure S2: Computational Analysis	S7-34
**VII.**	Supplementary Figure S3: Synthesis of **1a**	S34-39
**VIII.**	Supplementary Figure S4: Quantum Yield of **1a** and **2a**	S39-40
**IX.**	Supplementary Figure S5: Probe **1a** Chemoselectivity	S40-43
**X.**	Supplementary Figure S6: Flow Cytometry Cell Viability	S43-45
**XI.**	Supplementary Figure S7: Localization of Probe **1a** in HeLa cells	S45-46
**XII.**	Supplementary Figure S8: Imaging of HeLa cells with Exogenous MGO	S47
**XIII.**	Supplementary Figure S9: Endogenous Imaging of MGO in HeLa cells	S48
**XIV.**	Supplementary Figure S10: Probe **1a** Reactivity with Cellular Modifiers	S49-51
**XV.**	Supplementary Figure S11: Reversible Monitoring of MGO in HeLa cells	S52
**XVI.**	Supplementary Figure S12: Imaging of Murinal Brain Tissue	S52
**XVII.**	References	S53
**XVIII.**	Characterization of Synthesized Compounds	S54-67

I. General. All commercial materials (Sigma-Aldrich, Ambeed, and ThermoFisher) were used without further purification. All solvents were reagent or HPLC (Fisher) grade. Percent conversions refer to chromatographically pure compounds. Reaction progress was monitored by TLC plates (TLC Silica gel 60 F_{254}) and visualized with UV lamps.

II. Materials. AV/PI stains (AV-PacificBlue and AV-FITC) were purchased from Biolegend. HeLa cells were obtained from the Salaita lab in the Department of Chemistry at Emory University. All small molecules were purchased from CombiBlocks or Ambeed with the exception of MGO which was purchased from Oakwood and subsequently purified as previously reported.[1,2]

MGO Purification

Part 1: Anion Exchange Resin Preparation

1. To a beaker, add 8 g Na_2CO_3, 200 L H_2O, and 100 g Amberlite™ IRA-400 chloride form (cas: 60177-39-1). Cover the top of the beaker with aluminum foil and stir overnight.
2. Use a Hirsch funnel to filter off resin via vacuum filtration. Rinse resin with H_2O to remove excess Na_2CO_3 until pH drops to neutral (~1-2 L). Leave resin on vacuum filtration and allow to dry overnight.

Part 2: Distillation and Neutralization

1. Distill a 40% solution of MGO using reduced pressure from a water aspirator. Water will distill first and then the greenish vapors of methylglyoxal will begin to condense. Collect both fractions in the same flask using an ice bath below the receiving flask to condense the methylglyoxal vapors.
2. Acidic components must be removed by treating distillate with Amberlite™ IRA-400 resin (carbonate form, prepared in Part 1). Use ~100 mg of resin for every 100 mL of MGO distillate. Add ~50 mL H_2O per 100 mL of MGO distillate to decrease viscosity and promote stirring. This was stirred for 4 hours. Resin was removed via vacuum filtration and filtrate was stored at -20 °C.

Part 3: Quantification via Titration

1. Dilute 2 mL of purified MGO with 98 mL H_2O
2. To a 250 mL Erlenmeyer flask add 25 mL of diluted MGO and 2-3 drops of phenolphthalein TS and ½ drop of 0.1 N NaOH. This solution should be slightly pink.
3. Add 200 µL 30% H_2O_2 and 40 mL 0.1 N NaOH to the flask. Stir at RT for 20 minutes. The solution should be dark pink.
4. Titrate with 0.1 N HCl until solution turns clear. This should take ~30-40 mL total but varies based on earlier distillation.
5. Repeat steps 2-4 two-three more times, average the volume of HCL added
6. Calculation follows as:
    a. Calculate moles of HCl
    b. Subtract moles of HCl from moles of NaOH initially added
    c. Each mol of MGO produces 2 moles of acid, thus divide answer from part B by 2 to get moles of MGO
    d. Multiply answer by 4 to determine moles of MGO in the total 100 mL diluted solution
    e. The 100 mL dilute MGO solution contained 2 mL of concentrated MGO, thus dividing by 2 gives the final molarity of purified MGO.

III. Analytical Methods.

NMR: NMR spectra were recorded on a 400 MHz or 600 MHz Bruker NMR spectrometer. Proton chemical shifts were referenced to residual $CDCl_3$ at 7.26 ppm and carbon chemical shifts were referenced to $CDCl_3$ at 77.16 ppm. Spectra were processed using MestReNova ver. 12.0.4 and TOPSPIN software. The following abbreviations (or combinations thereof) are used to refer to multiplicities: s = singlet, d = doublet, t = triplet, q = quartet, p = quintet, and m = multiplet. Coupling constants (*J*), are reported in Hertz units (Hz).

HPLC: High performance liquid chromatography (HPLC) was conducted with an Agilent 1100 series equipped with a 5 µm particle size, C-18 reversed-phase column. All separations involved a mobile phase of water with 0.1% formic acid (solvent A) and acetonitrile with 0.1% formic acid (solvent B). The HPLC method employed a linear gradient of 0-60% solvent B over 30 minutes at ambient temperature with a flow rate of 1 mL/min. The eluent was monitored by absorbance at 220 nm and 280 nm.

HRMS. High resolution MS data were acquired on Thermo Exactive Plus using a heated electrospray source. The solution was infused at a rate of 10-25 µL min^{-1} electrospray using 3.3 kV. The typical settings were Capillary temp 320 °C. S-lens RF level was between 30-80 with an AGC setting of 1 E6. The maximum injection time was set to 50 ms. Spectra were taken at 140,000 resolutions at m/z 200 using Tune software and analyzed with ThermoFischer's Freestyle software. ver. 1.8.63.0.

Fluorimeter: Fluorescence spectroscopy was performed with an Agilent Cary Eclipse. Probe **1a** had an excitation of 501 nm and an emission of 526 nm.

Microscopy: Confocal Imaging with fluorescence lifetime imaging microscopy (FLIM) was performed on a Stellaris® 8 Leica DMi8 microscope.

Cells were plated in an IBIDI 8-well glass bottom chamber at a density of 25,000 cells per well in media and allowed to adhere overnight at 37 °C, 5% CO_2. The media was aspirated and samples pre-dissolved in DMSO were introduced into each well at the respective concentrations. Incubation was performed at 37 °C, 5% CO_2.

Cells were imaged live using an incubator-equipped Leica Stellaris® 8 microscope (20x objective) with fast lifetime contrast (FALCON) module. Samples were excited using an 80 MHz pulsed white light laser tuned to 501 nm for **1a**, 405 nm for Hoechst, and 647 nm for Cell Mask. Emitted photons were detected using HyD® X (GaAsP hybrid photocathode).

IV. Cell Culture Technique: Cells were maintained at 37 °C and 5% CO_2. HeLa cells were obtained from the Salaita lab in the Department of Chemistry at Emory University. HeLa cells were cultured in RPMI 1640 supplemented with 10% (V/V) fetal bovine serum (FBS) and 1% (V/V) penicillin/streptomycin (100 µg/mL).

V. Supplementary Figure S1: Reported MGO Probes

Probe / Structure	Ex / Em (nm)	LOD (µM)	Response Time	Detection Conditions	Reference
OPD-based and other reported fluorescent probes for MGO					
**CMFP** 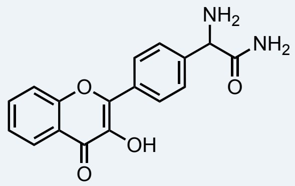	350 / 525→440 (dual-channel ratiometric)	0.24	90 min	Ratiometric OPD scaffold operating in 9:1 PBS/DMSO (100 mM, pH 7.4) at 37 °C; a 10 µM working concentration of sensor was exposed to 500 µM MGO to elicit the dual-wavelength shift	*Wang et al., Anal. Chem. 2019, 91, 5646–5653*
**NI-OPD** 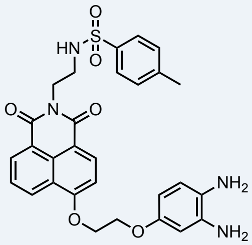	380 / 460 (turn-on) excitation: 780 nm (Two Photon)	5.6×10^{-4}	2 h	Naphthalimide-OPD scaffold with two-photon capability (780 nm); 10 µM sensor deployed in 1:9 DMF/PBS (10 mM, pH 7.4) at 37 °C against a 30 µM MGO challenge	*Yang et al., Chem. Sci. 2018, 9, 6758–6764*
**NP** 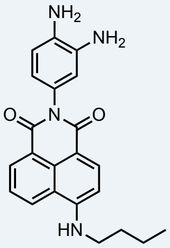	440 / 550 (turn-on) excitation: 760 nm (Two Photon)	1.47	1 h	Two-photon OPD scaffold (760 nm); responses recorded in 1:19 DMSO/aqueous buffer (pH 7.4) using 5 µM sensor against 150 µM MGO	*Gao et al., J. Fluoresc. 2019, 29, 155–163*
**PND-1** 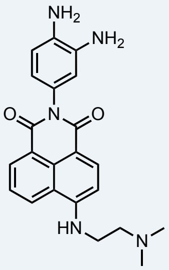	440 / 528 (turn-on) excitation: 880 nm (Two Photon)	0.077	12 h	Two-photon naphthalimide-OPD scaffold (880 nm); 5 µM sensor in 1:9 DMSO/PBS (10 mM, pH 7.4) at 37 °C; a 12 h equilibration period with 50 µM MGO was necessary for full response development	*Tang et al., Anal. Methods 2015, 7, 2386–2390*

Probe / Structure	Ex / Em (nm)	LOD (µM)	Response Time	Detection Conditions	Reference
**MBo** 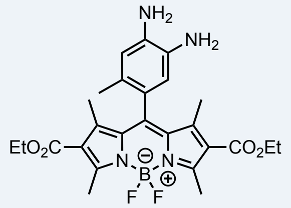	480 / 532 (turn-on)	0.050–0.100	1 h	BODIPY-OPD scaffold evaluated entirely in aqueous PBS (pH 7.4) at 37 °C; 10 µM sensor paired with 50 µM MGO; absence of organic co-solvent is a notable feature	*Wang et al., J. Am. Chem. Soc. 2013, 135, 12429–12433*
**L** 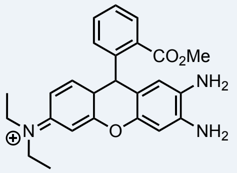	520 / 642 (fluorescence turn-off)	N.A.	N.A.	Rhodamine-based turn-off sensor; 5 µM sensor in 3:7 EtOH/Tris-HCl (10 mM, pH 7.4); unlike most MGO probes, emission decreases rather than increases upon analyte binding	*Liu et al., Dyes Pigm. 2017, 138, 23–29*
**DAF-2** 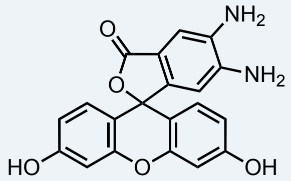	435 / 510 (turn-on)	0.70	30 min	Fluorescein-OPD scaffold; 10 µM sensor assessed against 50 µM MGO in undiluted aqueous PBS (100 mM, pH 7.4) at 37 °C; one of the faster-responding probes in this series	*Shaheen et al., Biochem. Soc. Trans. 2014, 42, 548–555*
**DAR-1** 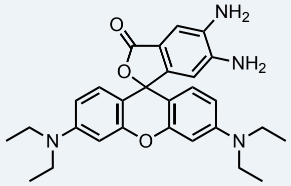	545 / 570 (turn-on)	N.A.	30 min	Rhodamine-OPD scaffold with red-shifted excitation; 10 µM sensor against 50 µM MGO in aqueous PBS (100 mM, pH 7.4) at 37 °C; shares the 30 min response window of the DAF-2 analog	*Shaheen et al., Biochem. Soc. Trans. 2014, 42, 548–555*
**MEBTD** 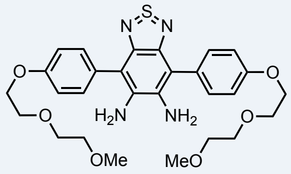	496 / 650 (near-IR turn-on)	0.018	N.A.	Near-infrared activatable scaffold; 50 µM sensor combined with an equimolar (50 µM) MGO load in 2:3 DMAC/PBS (10 mM, pH 7.4); long-wavelength emission reduces tissue autofluorescence	*Ding et al., Anal. Chem. 2019, 91, 15577–15584*

Probe / Structure	Ex / Em (nm)	LOD (μM)	Response Time	Detection Conditions	Reference
**DBTPP** 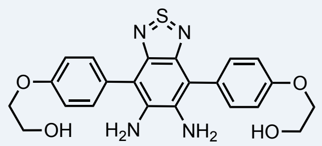	500 / 650 (near-IR turn-on)	0.262	N.A.	Near-infrared activatable scaffold; 25 μM sensor challenged with 60 μM MGO in 2:3 DMAC/PBS (10 mM, pH 7.4); developed for tumor-associated MGO imaging	*Dang et al., Chem. Commun. 2020, 56, 707–710*
Guanidine-based fluorescent probes for GOS					
**NAP-DCP-1** 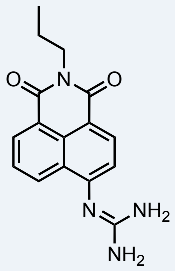	425 / 564 (turn-on)	0.72 (MGO) 0.58 (GO)	30 min	First-generation naphthalimide-guanidine sensor; 2 μM probe evaluated at ambient temperature in PBS (10 mM, pH 7.4) against 200 μM MGO; signal arises from a reversible, equilibrium-driven covalent adduct	*Xu et al., Anal. Chem. 2020, 92, 13829–13838*
**NAP-DCP-3** 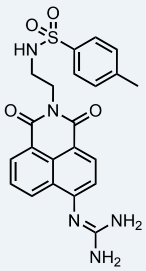	425 / 564 (turn-on)	0.13 (MGO) 0.16 (GO)	30 min	Optimized naphthalimide-guanidine analog; 2 μM probe at ambient temperature in PBS (10 mM, pH 7.4) against 200 μM MGO; ~5-fold lower detection threshold than the first-generation NAP-DCP-1	*Xu et al., Anal. Chem. 2020, 92, 13829–13838*
**ANC-DCP-1** 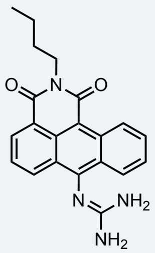	525 / 615 (turn-on)	12.6 (MGO) 12.1 (GO)	2 h	Anthracenecarboximide-guanidine analog with a lower pKa enabling function under weakly acidic conditions; 5 μM probe against 1 mM analyte at pH 6.0 and 37 °C; demonstrated in diabetic urine and Manuka honey	*Chen et al., RSC Adv. 2022, 12, 9473–9477*

Probe / Structure	Ex / Em (nm)	LOD (μM)	Response Time	Detection Conditions	Reference
**This work — dicyano-BODIPY platform (a-PET gated, reversible, visible-light)**					
**Probe 1a (dicyano-BODIPY)**<br>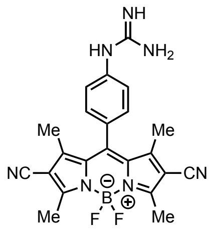 	**501 / 526 (turn-on)**	**~17**	**1.5 h**	DFT-guided acceptor-PET design; dicyano-BODIPY core paired with a phenylguanidine recognition unit.<br>20 μM probe in PBS (10 mM, pH 7.4); visible-light excitation at 501 nm avoids UV-associated cytotoxicity; fully reversible adduct formation enables real-time flux monitoring; biocompatibility confirmed in HeLa cells and murine brain tissue	***This work***

VI. Supplementary Figure S2: Computational Analysis

Computations were performed in Gaussian 10 through GaussView interface. DFT Ab initio calculations were performed at B3LYP/6-31+G(d,p) level with the default solvation set as water. Below are the reported geometry optimizations. The grey, white, red, blue, cyan, and rose balls represent the carbon, hydrogen, oxygen, nitrogen, fluorine, and boron atoms respectively.

1-phenylguanidine
HOMO = -0.23130 Hartree (-6.29 eV)

Center Number	Atomic Number	Atomic Type	Coordinates (Angstroms) X	Y	Z
1	6	0	-2.081498	-0.969163	0.000000
2	6	0	-0.686338	-0.969163	0.000000
3	6	0	0.011200	0.238588	0.000000
4	6	0	-0.686454	1.447097	-0.001199
5	6	0	-2.081279	1.447019	-0.001678
6	6	0	-2.778880	0.238813	-0.000682
7	1	0	-2.631257	-1.921480	0.000450
8	1	0	-0.136830	-1.921676	0.001315
9	1	0	1.110880	0.238668	0.000634
10	1	0	-2.631401	2.399300	-0.002631
11	1	0	-3.878484	0.238996	-0.000862
12	7	0	0.049027	2.719876	-0.001278
13	1	0	-0.191347	3.243711	-0.818479
14	6	0	-0.307288	3.491776	1.197935
15	7	0	0.303792	4.600421	1.464182
16	1	0	0.047116	5.131366	2.271780

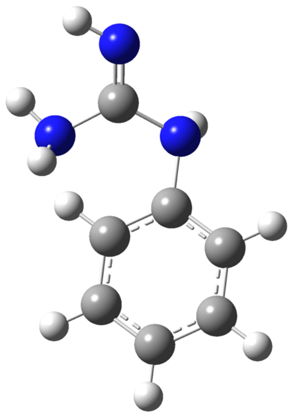

17	7	0	-1.361633	3.002171	2.097682
18	1	0	-0.971231	2.818509	2.999820
19	1	0	-2.078316	3.694773	2.179338

1-(3-fluorophenyl)guanidine
HOMO = -0.23522 Hartree (-6.40 eV)

Center Number	Atomic Number	Atomic Type	Coordinates (Angstroms) X	Y	Z
1	6	0	-4.988987	-0.572687	0.000000
2	6	0	-3.593827	-0.572687	0.000000
3	6	0	-2.896289	0.635064	0.000000
4	6	0	-3.593943	1.843573	-0.001199
5	6	0	-4.988768	1.843495	-0.001678
6	6	0	-5.686369	0.635289	-0.000682
7	1	0	-5.538746	-1.525004	0.000450
8	1	0	-1.796609	0.635144	0.000634
9	1	0	-5.538890	2.795776	-0.002631
10	1	0	-6.785973	0.635472	-0.000862
11	7	0	-2.858462	3.116352	-0.001278
12	1	0	-3.098836	3.640187	-0.818479
13	6	0	-3.214778	3.888251	1.197935
14	7	0	-2.603695	4.996895	1.464183
15	1	0	-2.860375	5.527842	2.271779
16	7	0	-4.269124	3.398648	2.097680
17	1	0	-3.878723	3.214986	2.999820
18	1	0	-4.985806	4.091250	2.179335
19	9	0	-2.919219	-1.742047	0.001614

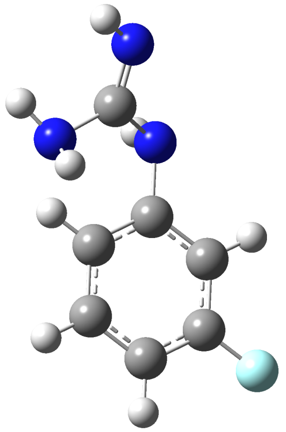

1-(3-(trifluoromethyl)phenyl)guanidine
HOMO = -0.23503 Hartree (-6.39 eV)

Center Number	Atomic Number	Atomic Type	Coordinates (Angstroms) X	Y	Z
1	6	0	-0.385029	-1.901265	-0.547919
2	6	0	1.014625	-1.971182	-0.549474
3	6	0	1.775016	-0.794220	-0.527377
4	6	0	1.135753	0.452659	-0.503724
5	6	0	-0.263901	0.522575	-0.502168
6	6	0	-1.024292	-0.654387	-0.524266
7	1	0	-0.965604	-2.799902	-0.564793
8	1	0	1.502716	-2.923201	-0.567531
9	1	0	2.843683	-0.847602	-0.528565
10	1	0	-0.751993	1.474594	-0.484107
11	7	0	1.933366	1.687234	-0.480544
12	1	0	2.114072	1.984984	-1.417929
13	6	0	1.191149	2.736804	0.232489
14	7	0	0.256166	2.374735	1.307484
15	1	0	-0.654342	2.226532	0.921465
16	1	0	0.569599	1.536416	1.753556
17	7	0	1.360782	3.979047	-0.086037
18	1	0	0.855875	4.693040	0.399021
19	6	0	-2.562373	-0.577556	-0.522557
20	9	0	-2.956914	0.557611	-1.137564
21	9	0	-3.066925	-1.643791	-1.179117
22	9	0	-3.011599	-0.579136	0.750507

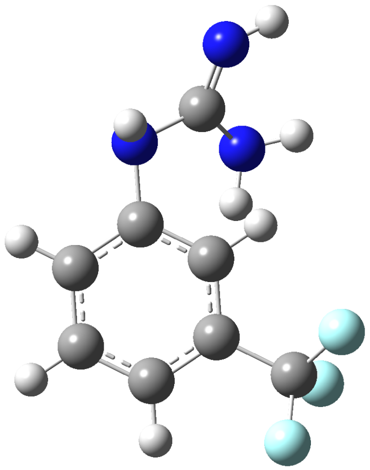

1-(3,5-bis(trifluoromethyl)phenyl)guanidine
HOMO = -0.25887 Hartree (-7.04 eV)

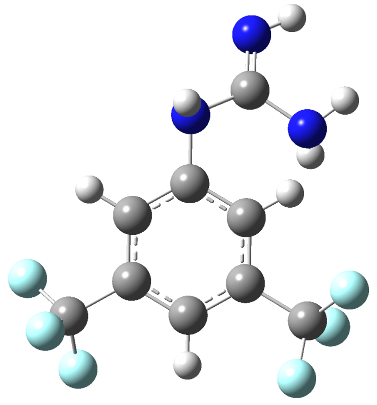

Center Number	Atomic Number	Atomic Type	Coordinates (Angstroms) X	Y	Z
1	6	0	-1.785287	0.939725	-0.829260
2	6	0	-0.398895	0.839132	-1.007363
3	6	0	0.405882	1.981518	-0.901477
4	6	0	-0.175734	3.224496	-0.617487
5	6	0	-1.562125	3.325089	-0.439382
6	6	0	-2.366902	2.182703	-0.545269
7	1	0	-2.399751	0.067488	-0.910106
8	1	0	1.464422	1.904713	-1.037464
9	1	0	-2.006201	4.274130	-0.222549
10	7	0	0.668438	4.422803	-0.506417
11	1	0	0.752978	4.855358	-1.404053
12	6	0	0.055246	5.368398	0.437376
13	7	0	0.801548	6.164888	1.131665
14	1	0	0.384410	6.808149	1.773702
15	7	0	-1.406017	5.408890	0.592205
16	1	0	-1.682864	4.771096	1.310937
17	1	0	-1.689300	6.335047	0.841168
18	6	0	-3.890409	2.293245	-0.349550
19	6	0	0.240243	-0.526778	-1.319439
20	9	0	-4.191300	2.108425	0.953449
21	9	0	-0.495892	-1.507561	-0.754931
22	9	0	1.494190	-0.561375	-0.820521
23	9	0	-4.509076	1.350878	-1.092313
24	9	0	0.282714	-0.708788	-2.656439
25	9	0	-4.306394	3.517335	-0.738215

2,6-trifluoromethyl phenylguanidine
HOMO = -0.24316 Hartree (-6.62 eV)

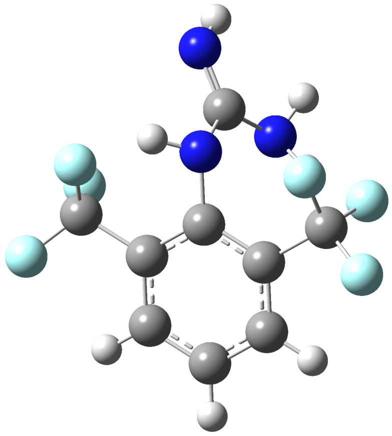

Center Number	Atomic Number	Atomic Type	Coordinates (Angstroms) X	Y	Z
1	6	0	-1.790821	-0.439075	-0.902148
2	6	0	-0.397327	-0.505660	-0.769248
3	6	0	0.367582	0.667766	-0.812905
4	6	0	-0.261003	1.907779	-0.989460
5	6	0	-1.654497	1.974364	-1.122362
6	6	0	-2.419406	0.800938	-1.078705
7	1	0	-2.374846	-1.335012	-0.868816
8	1	0	0.082612	-1.452438	-0.634445
9	1	0	-3.483369	0.851777	-1.180178
10	7	0	0.541349	3.138646	-1.035252
11	1	0	0.404052	3.593503	-1.915169
12	6	0	0.123519	4.036621	0.051007
13	7	0	-0.403873	3.478243	1.304391
14	1	0	-1.396071	3.376404	1.232473
15	1	0	0.011698	2.584244	1.471921
16	7	0	0.219932	5.318212	-0.096064
17	1	0	-0.064310	5.929080	0.642887
18	6	0	-2.345249	3.337015	-1.316381
19	6	0	1.898895	0.594595	-0.666863
20	9	0	-2.661622	3.856793	-0.111292
21	9	0	2.351000	1.729991	-0.093298
22	9	0	2.227186	-0.461965	0.106705
23	9	0	-3.469990	3.171759	-2.044494
24	9	0	-1.509666	4.177025	-1.963439
25	9	0	2.460883	0.451614	-1.885971

1-(2,6-dinitrophenyl)guanidine
HOMO = -0.25218 Hartree (-6.86 eV)

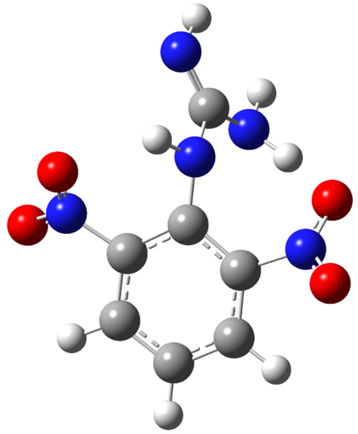

Center Number	Atomic Number	Atomic Type	Coordinates (Angstroms) X	Y	Z
1	6	0	-0.673641	1.136881	-0.478781
2	6	0	0.712494	1.177489	-0.118136
3	6	0	1.139902	2.472463	0.355125
4	6	0	0.308383	3.591896	0.356342
5	6	0	-1.008456	3.505025	-0.078318
6	6	0	-1.493274	2.266847	-0.476702
7	1	0	0.729200	4.530076	0.694826
8	1	0	-1.650683	4.377166	-0.079426
9	1	0	-2.529197	2.134784	-0.759930
10	7	0	1.560891	0.116775	-0.112691
11	1	0	2.319316	0.205289	0.563827
12	6	0	1.933503	-0.787228	-1.139804
13	7	0	2.837953	-1.616879	-0.798173
14	1	0	3.123324	-2.199202	-1.581962
15	7	0	1.453373	-0.492187	-2.417386
16	1	0	1.865570	-1.106842	-3.110074
17	1	0	0.441623	-0.530652	-2.482432
18	7	0	-1.356376	-0.142016	-0.714800
19	7	0	2.512489	2.742058	0.812168
20	8	0	-2.585178	-0.143908	-0.802419
21	8	0	2.883366	3.913617	0.842409
22	8	0	3.217606	1.796082	1.206375
23	8	0	-0.659375	-1.162436	-0.803307

1-(3,5-dihydroxyphenyl)guanidine
HOMO = -0.23206 Hartree (-6.31 eV)

Center Number	Atomic Number	Atomic Type	Coordinates (Angstroms) X	Y	Z
1	6	0	-2.487264	-1.361189	0.000000
2	6	0	-1.092104	-1.361189	0.000000
3	6	0	-0.394566	-0.153438	0.000000
4	6	0	-1.092220	1.055071	-0.001199
5	6	0	-2.487045	1.054993	-0.001678
6	6	0	-3.184646	-0.153213	-0.000682
7	1	0	-3.037023	-2.313506	0.000450
8	1	0	0.705114	-0.153358	0.000634
9	1	0	-3.037167	2.007274	-0.002631
10	7	0	-0.356739	2.327850	-0.001278
11	1	0	-0.597114	2.851685	-0.818479
12	6	0	-0.713055	3.099749	1.197935
13	7	0	-0.101973	4.208393	1.464183
14	1	0	-0.358652	4.739340	2.271779
15	7	0	-1.767401	2.610146	2.097680
16	1	0	-1.377000	2.426484	2.999820
17	1	0	-2.484084	3.302748	2.179335
18	8	0	-4.614646	-0.152975	-0.000916
19	1	0	-4.935391	-0.755962	-0.675550
20	8	0	-0.377520	-2.599844	0.001710
21	1	0	-0.409540	-2.989485	-0.875077

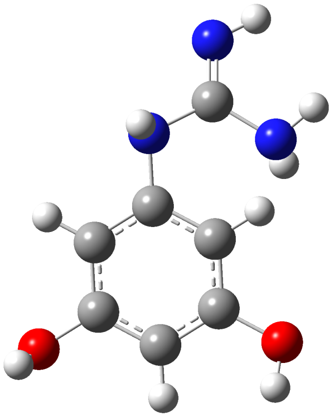

External Hydrate Product (MGH-DH)
4-methyl-2-(phenylamino)-4,5-dihydro-1*H*-imidazole-4,5-diol
HOMO = -0.24212 Hartree (-6.58 eV)

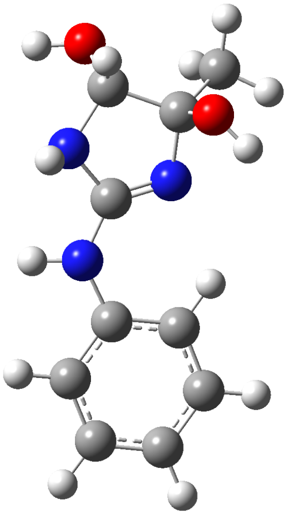

Center Number	Atomic Number	Atomic Type	Coordinates (Angstroms) X	Y	Z
1	6	0	-2.588405	-1.924633	-0.018179
2	6	0	-1.918132	-1.138418	0.921064
3	6	0	-1.843831	0.250549	0.786152
4	6	0	-2.463238	0.867757	-0.311184
5	6	0	-3.138215	0.079980	-1.259742
6	6	0	-3.197098	-1.304048	-1.113571
7	1	0	-2.634569	-3.002994	0.097690
8	1	0	-1.435222	-1.607090	1.773936
9	1	0	-1.310644	0.856857	1.505768
10	1	0	-3.621053	0.555390	-2.111008
11	1	0	-3.722693	-1.896406	-1.856938
12	7	0	-2.450382	2.261710	-0.524213
13	1	0	-2.806101	2.569332	-1.418060
14	6	0	-1.459299	5.453810	0.717726
15	1	0	-2.105082	6.250543	1.102084
16	6	0	-1.926912	3.258681	0.251891
17	7	0	-1.197650	3.138798	1.307898
18	7	0	-2.204059	4.579518	-0.190252
19	1	0	-3.193890	4.792945	-0.271615
20	6	0	-1.028202	4.486398	1.860942
21	6	0	0.380082	4.694151	2.397424
22	1	0	0.479657	5.694506	2.826315
23	1	0	0.572595	3.951493	3.177912
24	1	0	1.118724	4.571332	1.603121
25	8	0	-2.003331	4.692382	2.896837
26	1	0	-2.044263	3.871882	3.408987
27	8	0	-0.316628	5.990313	0.076855
28	1	0	-0.566794	6.216373	-0.829000

External Eliminated Product (MGH-1)
5-methyl-2-(phenylamino)-3,5-dihydro-4*H*-imidazol-4-one
HOMO = -0.25717 Hartree (-6.99 eV)

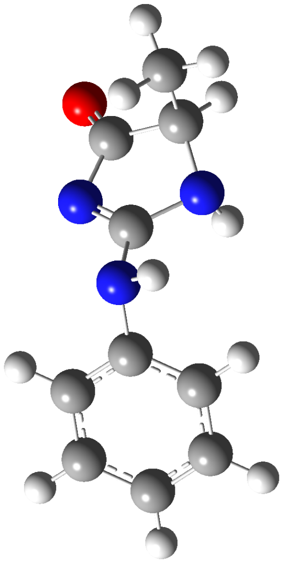

Center Number	Atomic Number	Atomic Type	Coordinates (Angstroms) X	Y	Z
1	6	0	-3.106562	-1.621243	0.066876
2	6	0	-1.835834	-1.390249	0.610764
3	6	0	-1.148774	-0.206850	0.308379
4	6	0	-1.732441	0.745555	-0.537896
5	6	0	-3.003168	0.514559	-1.081786
6	6	0	-3.690228	-0.668840	-0.779400
7	1	0	-3.631148	-2.524794	0.297754
8	1	0	-1.390190	-2.117432	1.256912
9	1	0	-0.178545	-0.030481	0.723650
10	1	0	-3.448810	1.241741	-1.727935
11	1	0	-4.660457	-0.845209	-1.194672
12	7	0	-1.011749	1.986883	-0.855082
13	1	0	-1.274355	2.300959	-1.767439
14	6	0	-2.000335	5.130618	0.723706
15	1	0	-2.797730	5.748540	1.080406
16	6	0	-1.352916	3.018273	0.135241
17	6	0	-1.175938	4.480563	1.813763
18	8	0	-0.942572	4.942325	2.960884
19	6	0	-1.078866	5.996027	-0.155818
20	1	0	-0.276417	5.395519	-0.530474
21	1	0	-0.680624	6.799676	0.427664
22	1	0	-1.639315	6.394175	-0.975741
23	7	0	-0.694072	3.207805	1.261157
24	7	0	-2.490123	3.962830	-0.005438
25	1	0	-3.296180	3.560565	0.428676

MGH-2
2-amino-5-methyl-1-phenyl-1,5-dihydro-4*H*-imidazol-4-one
HOMO: -0.24680 Hartree (-6.71 eV)

Center Number	Atomic Number	Atomic Type	Coordinates (Angstroms) X	Y	Z
1	6	0	0.627482	4.832539	-0.651730
2	1	0	1.328951	5.269168	0.070858
3	7	0	1.205302	3.621088	-1.257948
4	6	0	1.457918	3.914807	-2.576195
5	7	0	1.104276	5.118658	-2.987160
6	6	0	0.568196	5.768505	-1.889032
7	8	0	0.112307	6.898225	-1.848335
8	6	0	-0.735695	4.614761	-0.000337
9	1	0	-0.663830	3.958927	0.872784
10	1	0	-1.129240	5.584050	0.317401
11	1	0	-1.439550	4.174225	-0.713292
12	7	0	2.026832	3.013555	-3.413175
13	1	0	2.518836	2.215169	-3.041534
14	1	0	2.328060	3.382323	-4.303163
15	6	0	1.507915	2.422282	-0.563311
16	6	0	1.118916	1.175467	-1.080188
17	6	0	2.176699	2.477449	0.669865
18	6	0	1.424884	0.003192	-0.384908
19	1	0	0.559630	1.129849	-2.009249
20	6	0	2.457279	1.302466	1.368120
21	1	0	2.479057	3.437176	1.076233
22	6	0	2.091715	0.060162	0.841455
23	1	0	1.119601	-0.954828	-0.795476
24	1	0	2.973385	1.359371	2.321962
25	1	0	2.317530	-0.852179	1.384622

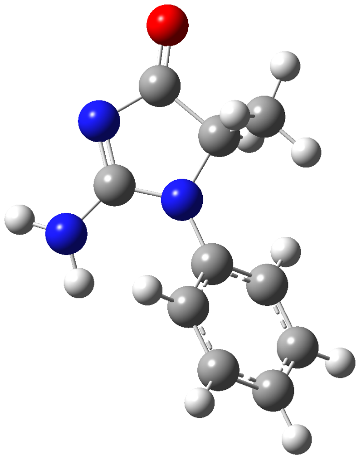

Internal Eliminated Product (MGH-3)
2-amino-5-methyl-3-phenylimidazolidin-4-one
HOMO = -0.24770 Hartree (-6.74 eV)

Center Number	Atomic Number	Atomic Type	Coordinates (Angstroms) X	Y	Z
1	7	0	1.306917	3.643402	-1.357400
2	6	0	1.655999	3.903041	-2.705119
3	7	0	1.307224	5.054272	-3.157567
4	7	0	2.289840	2.927059	-3.433327
5	1	0	2.872822	2.273214	-2.928997
6	1	0	2.679143	3.267098	-4.302460
7	6	0	1.531821	2.446910	-0.614474
8	6	0	1.075051	1.215388	-1.103504
9	6	0	2.186165	2.515332	0.621698
10	6	0	1.300277	0.051649	-0.365004
11	1	0	0.545606	1.175084	-2.049732
12	6	0	2.389200	1.348875	1.360719
13	1	0	2.516171	3.476016	1.000289
14	6	0	1.954688	0.114953	0.868270
15	1	0	0.948830	-0.901623	-0.748482
16	1	0	2.890854	1.406040	2.322055
17	1	0	2.119397	-0.790415	1.444655
18	6	0	0.611207	5.743682	-2.069873
19	1	0	1.172053	6.648273	-1.795748
20	6	0	-0.823127	6.144907	-2.433733
21	1	0	-1.307306	6.631052	-1.581898
22	1	0	-0.806703	6.834312	-3.281957
23	1	0	-1.409197	5.265220	-2.717919
24	6	0	0.652799	4.786670	-0.873226
25	8	0	0.220528	4.941648	0.252267

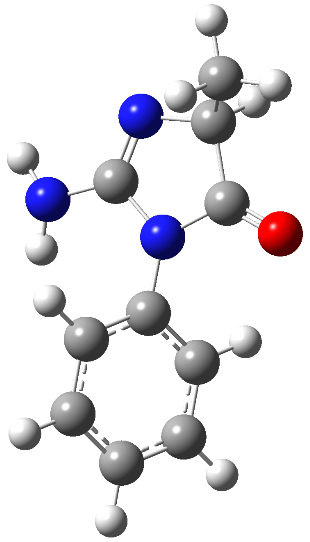

THP
5,6-dihydroxy-4,6-dimethyl-2-(phenylamino)-3,4,5,6-tetrahydropyrimidine-4-carboxylic acid
HOMO: -0.21564 Hartree (-5.86 eV)

Center Number	Atomic Number	Atomic Type	Coordinates (Angstroms) X	Y	Z
1	6	0	-0.480923	-0.530224	0.120040
2	6	0	-0.034802	0.240757	-0.957951
3	6	0	0.094193	1.621395	-0.830197
4	6	0	-0.225462	2.264724	0.380672
5	6	0	-0.682213	1.493910	1.462305
6	6	0	-0.798262	0.108520	1.320192
7	1	0	-0.580915	-1.606868	0.023302
8	1	0	0.217936	-0.231979	-1.902764
9	1	0	0.450875	2.209557	-1.673350
10	1	0	-0.945930	1.986536	2.386666
11	1	0	-1.150851	-0.474563	2.166671
12	7	0	-0.052375	3.660695	0.418799
13	1	0	0.212909	4.078664	-0.461578
14	6	0	-0.234041	4.573147	1.443603
15	7	0	-0.856522	4.267442	2.532677
16	6	0	-0.626174	4.464849	4.929956
17	1	0	0.346182	3.964478	4.906786
18	1	0	-0.684442	5.141396	5.790355
19	1	0	-1.392157	3.694012	5.052166
20	6	0	-1.205776	7.726363	1.596144
21	1	0	-2.066672	7.059293	1.615751
22	1	0	-1.395292	8.555099	2.281964
23	1	0	-1.079865	8.120116	0.583221
24	8	0	1.390993	6.120900	3.779635
25	1	0	1.462455	5.908902	4.718145
26	6	0	-0.903698	5.220083	3.621360
27	6	0	0.067243	6.949940	2.004678
28	6	0	0.034860	6.447649	3.468303
29	1	0	-0.333850	7.248525	4.118705
30	8	0	-2.228100	5.784274	3.697492
31	1	0	-2.832850	5.077666	3.426419
32	6	0	1.275106	7.880153	1.827598
33	8	0	2.176887	7.698289	1.036290
34	8	0	1.200508	8.968951	2.617742
35	1	0	1.995896	9.501903	2.449501
36	7	0	0.285560	5.817271	1.107604
37	1	0	1.200201	5.827876	0.665428

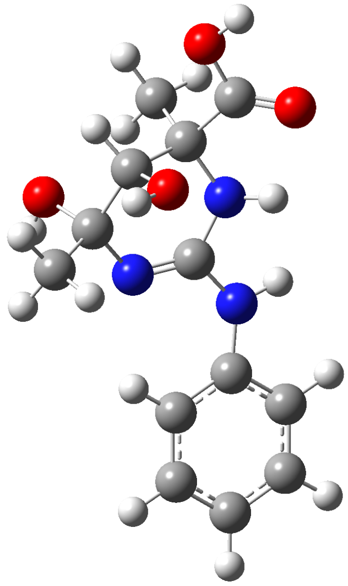

APY
4,6-dimethyl-2-(phenylamino)pyrimidin-5-ol
HOMO: -0.20002 Hartree (-5.44 eV)

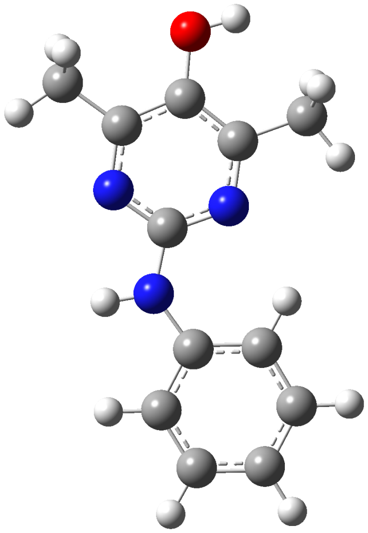

Center Number	Atomic Number	Atomic Type	Coordinates (Angstroms) X	Y	Z
1	6	0	-0.383312	-0.705005	-0.099533
2	6	0	-0.412386	0.153853	-1.203473
3	6	0	-0.422021	1.533723	-1.022836
4	6	0	-0.402835	2.092872	0.271277
5	6	0	-0.373692	1.230734	1.381704
6	6	0	-0.364301	-0.152389	1.182258
7	1	0	-0.375727	-1.781643	-0.238260
8	1	0	-0.427688	-0.250144	-2.211812
9	1	0	-0.444645	2.191264	-1.889233
10	1	0	-0.358882	1.648053	2.377615
11	1	0	-0.341687	-0.803927	2.051769
12	7	0	-0.414738	3.491684	0.351251
13	1	0	-0.437433	3.982759	-0.531911
14	6	0	-0.401371	4.368842	1.417150
15	6	0	-0.410570	6.587605	2.005877
16	6	0	-0.360380	4.840259	3.655129
17	6	0	-0.379473	6.202753	3.358511
18	7	0	-0.421234	5.667048	1.042706
19	7	0	-0.371663	3.918225	2.675393
20	6	0	-0.326087	4.342558	5.077765
21	1	0	0.569421	4.692611	5.608158
22	1	0	-1.205145	4.675644	5.645413
23	1	0	-0.315806	3.251913	5.079481
24	6	0	-0.432310	8.037873	1.615171
25	1	0	-1.307090	8.541723	2.041021
26	1	0	0.449484	8.558357	2.005058
27	1	0	-0.455294	8.123448	0.527975
28	8	0	-0.369652	7.200529	4.307300
29	1	0	-0.347763	6.819297	5.194089

CEA
(*Z*)-2-((amino(phenylamino)methylene)amino)propanoic acid
HOMO: -0.21518 Hartree (-5.85 eV)

Center Number	Atomic Number	Atomic Type	Coordinates (Angstroms) X	Y	Z
1	6	0	1.026846	3.590526	-2.637602
2	7	0	1.137948	4.858832	-2.866654
3	7	0	1.460107	2.721629	-3.629419
4	1	0	1.901838	1.863461	-3.324132
5	1	0	1.953880	3.210747	-4.364112
6	6	0	0.173519	1.651190	-1.257650
7	6	0	-0.390121	0.818670	-2.240717
8	6	0	0.351416	1.144666	0.042600
9	6	0	-0.741377	-0.495235	-1.924139
10	1	0	-0.558151	1.202220	-3.240104
11	6	0	-0.019402	-0.163810	0.350338
12	1	0	0.783322	1.784008	0.808001
13	6	0	-0.561488	-0.997216	-0.632465
14	1	0	-1.174874	-1.125262	-2.695932
15	1	0	0.125540	-0.534009	1.361313
16	1	0	-0.843615	-2.018133	-0.394495
17	6	0	0.475053	5.898634	-2.095705
18	1	0	0.703883	6.836668	-2.609003
19	6	0	-1.066591	5.778035	-2.034700
20	1	0	-1.504693	6.660924	-1.556785
21	1	0	-1.447342	5.714842	-3.057870
22	1	0	-1.387120	4.887617	-1.489475
23	6	0	1.021711	6.074128	-0.677222
24	8	0	1.124308	5.201524	0.171867
25	8	0	1.377218	7.349129	-0.415469
26	1	0	1.683583	7.383008	0.507855
27	7	0	0.504796	3.001570	-1.493876
28	1	0	0.595970	3.592876	-0.670033

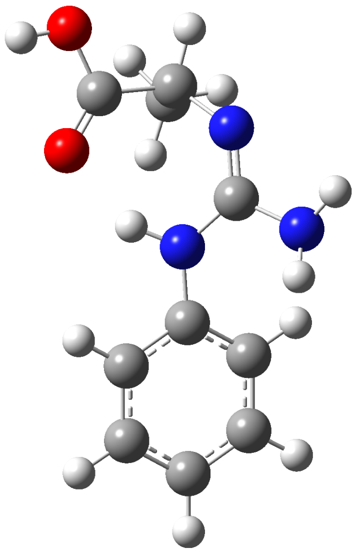

4-((4,5-dihydroxy-5-methyl-4,5-dihydro-1*H*-imidazol-2-yl)amino)benzonitrile (MGH-DH-cyano)
HOMO: -0.24921 hartree (-6.78 eV)

Center Number	Atomic Number	Atomic Type	Coordinates (Angstroms) X	Y	Z
1	6	0	-2.585886	-1.928906	-0.010801
2	6	0	-1.929824	-1.130123	0.940990
3	6	0	-1.862975	0.252183	0.795539
4	6	0	-2.467672	0.863584	-0.316465
5	6	0	-3.123243	0.065325	-1.273686
6	6	0	-3.181933	-1.312605	-1.126511
7	1	0	-1.461866	-1.599340	1.800137
8	1	0	-1.343254	0.863818	1.520275
9	1	0	-3.593217	0.531498	-2.136151
10	1	0	-3.690180	-1.917476	-1.869843
11	7	0	-2.458400	2.247930	-0.531725
12	1	0	-2.815259	2.556497	-1.425169
13	6	0	-1.465678	5.441278	0.717351
14	1	0	-2.112977	6.234951	1.105193
15	6	0	-1.935245	3.249731	0.249249
16	7	0	-1.205322	3.122932	1.300918
17	7	0	-2.213572	4.566430	-0.190571
18	1	0	-3.202135	4.782594	-0.279962
19	6	0	-1.029851	4.471921	1.857590
20	6	0	0.383247	4.672234	2.383550
21	1	0	0.491497	5.672608	2.809708
22	1	0	0.578119	3.929317	3.163400
23	1	0	1.115874	4.543748	1.584677
24	8	0	-1.999579	4.679159	2.893972
25	1	0	-1.985583	3.894940	3.461406
26	8	0	-0.323459	5.976314	0.079520
27	1	0	-0.585830	6.281440	-0.799326
28	6	0	-2.645881	-3.351265	0.148295
29	7	0	-2.697109	-4.507795	0.275800

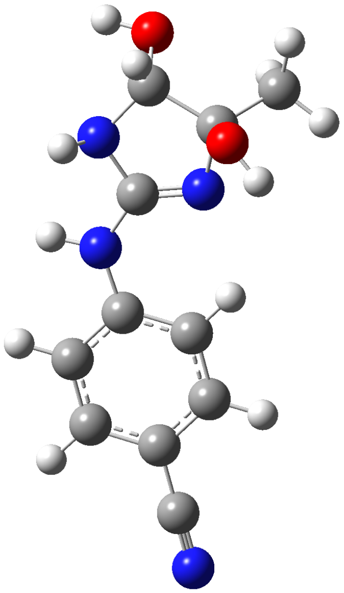

4-((5-methyl-4-oxo-4,5-dihydro-1*H*-imidazol-2-yl)amino)benzonitrile (MGH-1-cyano)
HOMO: -0.25443 Hartree (-6.92 eV)

Center Number	Atomic Number	Atomic Type	Coordinates (Angstroms) X	Y	Z
1	6	0	-2.883807	-1.996771	0.064494
2	6	0	-2.184089	-1.201036	0.987135
3	6	0	-2.053503	0.171934	0.799025
4	6	0	-2.634772	0.770927	-0.330327
5	6	0	-3.335889	-0.021401	-1.257019
6	6	0	-3.459882	-1.390292	-1.065835
7	1	0	-1.734671	-1.663130	1.859706
8	1	0	-1.507434	0.780708	1.506822
9	1	0	-3.788356	0.438080	-2.132538
10	1	0	-4.002075	-1.993308	-1.786135
11	7	0	-2.564683	2.151869	-0.603211
12	1	0	-2.966615	2.431299	-1.487839
13	6	0	-1.475905	5.324776	0.627847
14	1	0	-2.263510	5.882877	1.152154
15	6	0	-1.969714	3.151524	0.106186
16	6	0	-0.894862	4.277028	1.614779
17	8	0	-0.212064	4.554555	2.580055
18	6	0	-0.421931	6.285122	0.083206
19	1	0	0.349756	5.738010	-0.466152
20	1	0	0.050707	6.807143	0.919773
21	1	0	-0.868788	7.026352	-0.587001
22	7	0	-1.269515	2.995155	1.204054
23	7	0	-2.069053	4.440987	-0.384723
24	1	0	-2.910698	4.728399	-0.868124
25	6	0	-3.010331	-3.409616	0.268492
26	7	0	-3.116378	-4.557793	0.430210

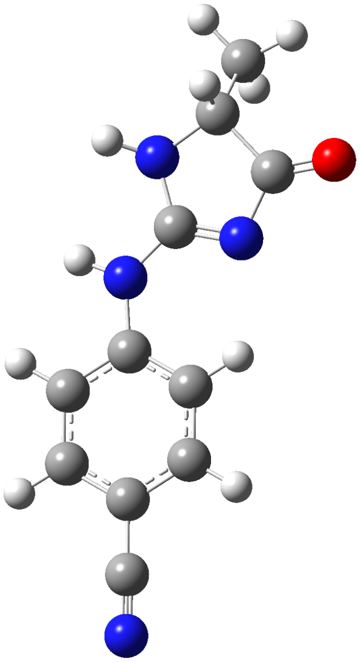

BODIPY-H
HOMO = -0.20559 Hartree (-5.59 eV)

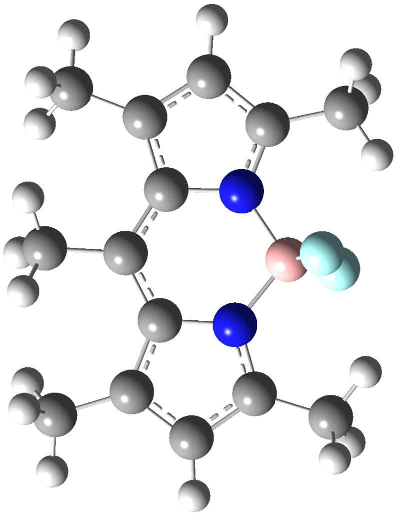

Center Number	Atomic Number	Atomic Type	Coordinates (Angstroms) X	Y	Z
1	6	0	5.305328	-2.014906	-0.069650
2	6	0	6.188500	-2.925052	-0.687343
3	7	0	5.715951	-1.266084	1.045345
4	6	0	8.459840	-4.144430	-0.473737
5	6	0	9.511348	-3.955984	0.444880
6	6	0	9.130845	-2.907956	1.339057
7	6	0	4.717130	-0.464538	1.438487
8	6	0	3.591219	-0.686572	0.587459
9	6	0	3.949659	-1.666075	-0.359382
10	6	0	7.452668	-3.195636	-0.121768
11	6	0	3.046771	-2.276600	-1.393390
12	1	0	3.186032	-3.357527	-1.460430
13	1	0	2.004551	-2.077481	-1.153720
14	1	0	3.229931	-1.853386	-2.389580
15	6	0	4.833369	0.486936	2.582122
16	1	0	4.257767	0.113959	3.436529
17	1	0	5.870125	0.618048	2.885289
18	1	0	4.393952	1.450202	2.315072
19	6	0	8.415422	-5.196415	-1.543780
20	1	0	9.191216	-5.939292	-1.367336
21	1	0	7.443863	-5.694979	-1.581199
22	1	0	8.608441	-4.767402	-2.535772
23	6	0	9.905592	-2.317454	2.472631
24	1	0	10.053355	-3.066152	3.257381
25	1	0	10.901343	-2.020985	2.133203
26	1	0	9.395092	-1.456335	2.896864
27	7	0	7.906105	-2.480792	0.999221
28	9	0	6.978403	-1.641194	3.070931
29	9	0	7.795413	-0.121418	1.547883
30	5	0	7.122503	-1.336806	1.714088
31	6	0	5.800421	-3.579234	-1.990430
32	1	0	5.289202	-4.536328	-1.827207
33	1	0	5.134119	-2.940611	-2.565579
34	1	0	6.676620	-3.774258	-2.604210
35	1	0	10.426575	-4.510071	0.460626
36	1	0	2.655452	-0.149639	0.720174

BODIPY-CO_2Et
HOMO = -0.22263 Hartree (-6.05 eV)
LUMO = -0.11108 Hartree (-3.02 eV)

Center Number	Atomic Number	Atomic Type	Coordinates (Angstroms) X	Y	Z
1	6	0	5.744297	-1.107782	0.606383
2	6	0	7.031361	-1.219258	1.173057
3	7	0	5.330771	-1.981631	-0.410814
4	6	0	9.352281	-2.347718	0.948584
5	6	0	9.770579	-3.404122	0.117644
6	6	0	8.653792	-3.803198	-0.677971
7	6	0	4.073731	-1.683566	-0.774492
8	6	0	3.628024	-0.566523	-0.004322
9	6	0	4.673343	-0.198248	0.864756
10	6	0	7.970744	-2.131458	0.648840
11	6	0	4.657733	0.975644	1.801015
12	1	0	5.593503	1.538330	1.759715
13	1	0	3.829767	1.637347	1.553335
14	1	0	4.504978	0.658094	2.840858
15	6	0	3.335753	-2.463620	-1.814283
16	1	0	3.233116	-1.869112	-2.728312
17	1	0	3.853877	-3.389168	-2.055715
18	1	0	2.324472	-2.688135	-1.469221
19	6	0	10.222820	-1.550384	1.878112
20	1	0	11.272056	-1.700405	1.632842
21	1	0	10.001793	-0.482449	1.820655
22	1	0	10.094260	-1.862921	2.922624
23	6	0	8.590681	-4.886664	-1.702796
24	1	0	9.093306	-4.560723	-2.620194
25	1	0	9.132752	-5.767430	-1.353393
26	1	0	7.561058	-5.147883	-1.939764
27	7	0	7.601999	-3.034363	-0.360531
28	9	0	6.288909	-2.990734	-2.389565
29	9	0	5.605938	-4.376614	-0.683877
30	5	0	6.186682	-3.142223	-1.002736
31	6	0	7.385466	-0.391047	2.384094
32	1	0	7.815959	0.578289	2.102749
33	1	0	6.504810	-0.195900	2.991911
34	1	0	8.108381	-0.906224	3.012265
35	6	0	11.086498	-4.054802	-0.007299
36	8	0	11.397454	-4.803587	-0.920242
37	8	0	11.937726	-3.764417	1.009528
38	6	0	2.318481	0.104101	-0.088296
39	8	0	1.902491	0.937537	0.702969

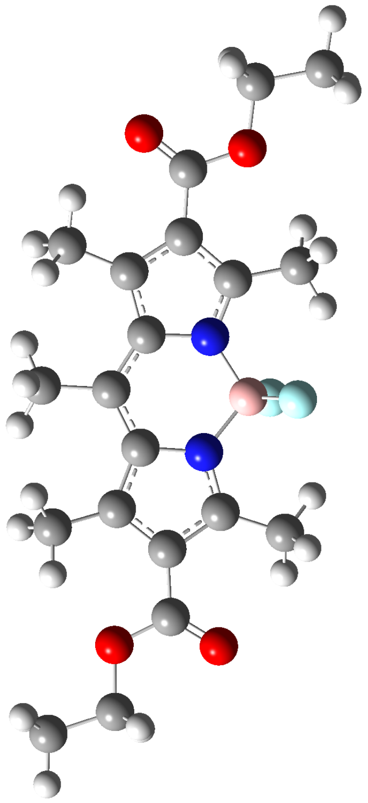

40	8	0	1.592077	-0.294924	-1.160885
41	6	0	0.285050	0.314823	-1.309335
42	6	0	-0.359160	-0.275256	-2.549655
43	1	0	0.413509	1.398474	-1.389148
44	1	0	-0.298944	0.114226	-0.406039
45	1	0	-1.349486	0.169265	-2.693844
46	1	0	0.241188	-0.070749	-3.441329
47	1	0	-0.481204	-1.358547	-2.454949
48	6	0	13.246686	-4.384520	0.937230
49	6	0	14.039866	-3.924663	2.145985
50	1	0	13.721724	-4.091760	-0.003899
51	1	0	13.116196	-5.470617	0.920710
52	1	0	15.033026	-4.385458	2.128638
53	1	0	14.168251	-2.837755	2.145654
54	1	0	13.543415	-4.215355	3.076834

BODIPY-NO_2
HOMO = -0.25338 Hartree (-6.89 eV)

Center Number	Atomic Number	Atomic Type	Coordinates (Angstroms) X	Y	Z
1	6	0	5.316084	-1.999546	-0.075985
2	6	0	6.191714	-2.918170	-0.692559
3	7	0	5.726183	-1.259031	1.046353
4	6	0	8.439309	-4.171978	-0.470033
5	6	0	9.468753	-3.978723	0.462258
6	6	0	9.116227	-2.936350	1.365162
7	6	0	4.737242	-0.447505	1.444322
8	6	0	3.635625	-0.666477	0.569922
9	6	0	3.969479	-1.636989	-0.385538
10	6	0	7.446855	-3.207480	-0.116420
11	6	0	3.050040	-2.225701	-1.413895
12	1	0	3.202563	-3.300716	-1.520293
13	1	0	2.011975	-2.040795	-1.141655
14	1	0	3.198714	-1.758946	-2.395862
15	6	0	4.858932	0.505558	2.584351
16	1	0	5.902820	0.679037	2.838951
17	1	0	4.369520	1.450533	2.340362
18	1	0	4.341699	0.103637	3.462333
19	6	0	8.376345	-5.244961	-1.516186
20	1	0	9.079061	-6.042888	-1.280843
21	1	0	7.374034	-5.668315	-1.597766
22	1	0	8.666728	-4.859431	-2.501691

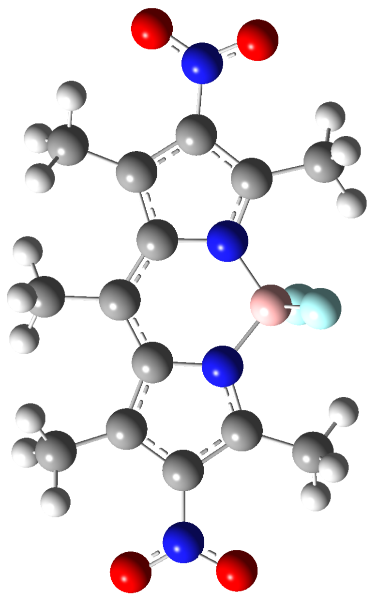

23	6	0	9.902189	-2.364814	2.495982
24	1	0	9.837612	-3.027239	3.366057
25	1	0	10.959009	-2.303681	2.229023
26	1	0	9.528818	-1.380979	2.774281
27	7	0	7.902806	-2.495463	1.006615
28	9	0	7.004828	-1.607356	3.066978
29	9	0	7.818946	-0.124530	1.506873
30	5	0	7.139539	-1.327050	1.708869
31	6	0	5.807922	-3.551091	-2.006834
32	1	0	5.276699	-4.499808	-1.860408
33	1	0	5.160015	-2.893026	-2.581673
34	1	0	6.688066	-3.753479	-2.613213
35	7	0	10.702896	-4.709755	0.531216
36	8	0	11.323333	-4.693030	1.600956
37	8	0	11.083676	-5.312670	-0.480391
38	7	0	2.378791	0.018810	0.684547
39	8	0	1.634732	0.046905	-0.304097
40	8	0	2.109429	0.550532	1.768251

BODIPY-CF_3
HOMO = -0.22960 Hartree (-6.25 eV)

Center Number	Atomic Number	Atomic Type	Coordinates (Angstroms) X	Y	Z
1	6	0	5.305329	-2.014906	-0.069650
2	6	0	6.188501	-2.925052	-0.687343
3	7	0	5.715952	-1.266084	1.045345
4	6	0	8.459841	-4.144430	-0.473737
5	6	0	9.511349	-3.955984	0.444880
6	6	0	9.130846	-2.907957	1.339057
7	6	0	4.717130	-0.464538	1.438488
8	6	0	3.591220	-0.686572	0.587459
9	6	0	3.949660	-1.666075	-0.359382
10	6	0	7.452668	-3.195637	-0.121768
11	6	0	3.046771	-2.276600	-1.393390
12	1	0	3.186032	-3.357528	-1.460430
13	1	0	2.004552	-2.077481	-1.153720
14	1	0	3.229932	-1.853386	-2.389580
15	6	0	4.833370	0.486936	2.582123
16	1	0	4.257767	0.113959	3.436530
17	1	0	5.870125	0.618048	2.885289
18	1	0	4.393953	1.450203	2.315072
19	6	0	8.415423	-5.196415	-1.543780

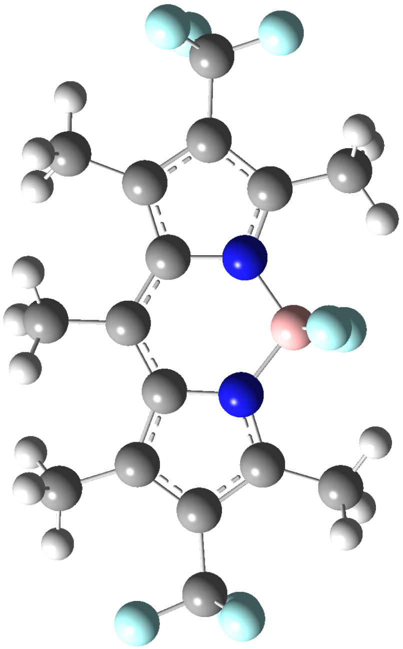

20	1	0	9.191216	-5.939292	-1.367337
21	1	0	7.443864	-5.694979	-1.581199
22	1	0	8.608442	-4.767402	-2.535772
23	6	0	9.905593	-2.317454	2.472631
24	1	0	10.053356	-3.066152	3.257381
25	1	0	10.901344	-2.020985	2.133203
26	1	0	9.395093	-1.456335	2.896865
27	7	0	7.906105	-2.480792	0.999221
28	9	0	6.978403	-1.641195	3.070931
29	9	0	7.795414	-0.121418	1.547883
30	5	0	7.122504	-1.336806	1.714089
31	6	0	5.800421	-3.579235	-1.990430
32	1	0	5.289203	-4.536328	-1.827208
33	1	0	5.134119	-2.940612	-2.565579
34	1	0	6.676621	-3.774258	-2.604210
35	6	0	10.828591	-4.753455	0.467543
36	6	0	2.265478	0.074123	0.775482
37	9	0	11.399708	-4.713838	-0.755059
38	9	0	11.668179	-4.209876	1.374247
39	9	0	10.572610	-6.035733	0.803307
40	9	0	1.415778	-0.249040	-0.222554
41	9	0	1.716470	-0.264789	1.961327
42	9	0	2.502010	1.403042	0.752498

BODIPY-CO_2H
HOMO = -0.23031 Hartree (-6.27 eV)

Center Number	Atomic Number	Atomic Type	Coordinates (Angstroms) X	Y	Z
1	6	0	5.298010	-2.010707	-0.068612
2	6	0	6.181182	-2.920853	-0.686305
3	7	0	5.708633	-1.261885	1.046383
4	6	0	8.452522	-4.140231	-0.472699
5	6	0	9.504030	-3.951785	0.445918
6	6	0	9.123527	-2.903757	1.340095
7	6	0	4.709812	-0.460339	1.439525
8	6	0	3.583902	-0.682373	0.588497
9	6	0	3.942342	-1.661876	-0.358344
10	6	0	7.445350	-3.191437	-0.120731
11	6	0	3.039453	-2.272401	-1.392352
12	1	0	3.178714	-3.353328	-1.459392
13	1	0	1.997234	-2.073282	-1.152682
14	1	0	3.222614	-1.849187	-2.388542

15	6	0	4.826052	0.491134	2.583160
16	1	0	4.250449	0.118158	3.437567
17	1	0	5.862807	0.622247	2.886327
18	1	0	4.386634	1.454401	2.316110
19	6	0	8.408104	-5.192216	-1.542742
20	1	0	9.183898	-5.935093	-1.366299
21	1	0	7.436545	-5.690779	-1.580161
22	1	0	8.601123	-4.763203	-2.534734
23	6	0	9.898274	-2.313255	2.473669
24	1	0	10.046037	-3.061953	3.258418
25	1	0	10.894025	-2.016786	2.134241
26	1	0	9.387774	-1.452136	2.897902
27	6	0	2.320915	0.042314	0.767619
28	8	0	2.051808	0.754712	1.720860
29	8	0	1.427962	-0.119526	-0.251362
30	1	0	0.647368	0.404949	-0.004834
31	6	0	10.759165	-4.711656	0.467512
32	8	0	11.112266	-5.541364	-0.356356
33	8	0	11.554523	-4.413067	1.532701
34	1	0	12.348205	-4.965941	1.436870
35	7	0	7.898787	-2.476593	1.000259
36	9	0	6.971085	-1.636996	3.071969
37	9	0	7.788095	-0.117219	1.548920
38	5	0	7.115185	-1.332607	1.715126
39	6	0	5.793103	-3.575035	-1.989392
40	1	0	5.281885	-4.532129	-1.826169
41	1	0	5.126801	-2.936413	-2.564541
42	1	0	6.669302	-3.770059	-2.603172

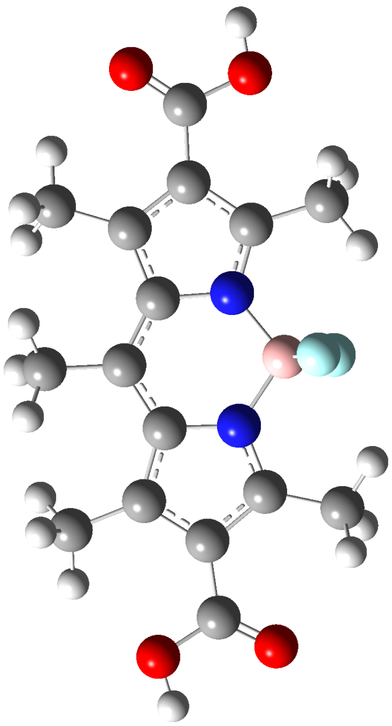

BODIPY-CN
HOMO = -0.24378 Hartree (-6.63 eV)
LUMO = -0.13286 Hartree (-5.80 eV)

Center Number	Atomic Number	Atomic Type	Coordinates (Angstroms) X	Y	Z
1	6	0	5.307885	-2.015106	-0.069506
2	6	0	6.179084	-2.939588	-0.681000
3	7	0	5.717749	-1.280074	1.055681
4	6	0	8.468735	-4.129655	-0.495260
5	6	0	9.507746	-3.937170	0.429010
6	6	0	9.128478	-2.907124	1.331695
7	6	0	4.720494	-0.479404	1.457399
8	6	0	3.608590	-0.688798	0.597473
9	6	0	3.959534	-1.647908	-0.365874
10	6	0	7.452167	-3.194266	-0.131391
11	6	0	3.019866	-2.140010	-1.425829
12	1	0	2.954051	-3.231702	-1.445880
13	1	0	2.017244	-1.747145	-1.241617
14	1	0	3.319756	-1.806050	-2.426339
15	6	0	4.809532	0.452664	2.617687
16	1	0	4.907788	-0.107528	3.553260
17	1	0	5.692260	1.092873	2.531924
18	1	0	3.914959	1.075696	2.669842
19	6	0	8.507499	-5.159639	-1.584604
20	1	0	9.378052	-5.806110	-1.451160
21	1	0	7.615925	-5.793300	-1.587417
22	1	0	8.593536	-4.702032	-2.577448
23	6	0	9.917382	-2.361487	2.473096
24	1	0	9.426913	-2.594443	3.423782
25	1	0	10.921263	-2.789626	2.476844
26	1	0	9.988536	-1.271952	2.406910
27	7	0	7.902984	-2.482642	0.992705
28	9	0	6.995570	-1.653915	3.076899
29	9	0	7.791449	-0.127777	1.553797
30	5	0	7.125593	-1.345264	1.721158
31	6	0	5.746575	-3.664003	-1.929418
32	1	0	5.245726	-4.608855	-1.682142
33	1	0	5.053430	-3.066003	-2.516125
34	1	0	6.597002	-3.898103	-2.565431
35	6	0	10.735285	-4.649909	0.473897
36	6	0	2.353671	-0.033904	0.712426
37	7	0	11.740349	-5.238899	0.505084
38	7	0	1.321268	0.499373	0.800722

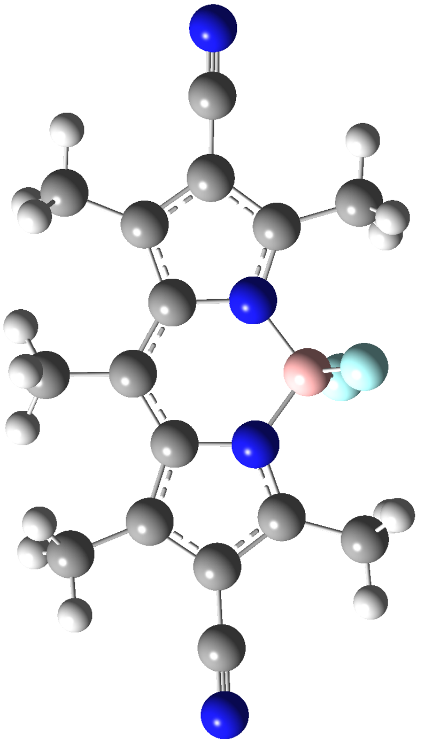

Probe 1a
HOMO = -0.22702 Hartree (-6.18 eV)
LUMO = -0.12451 Hartree (-3.39 eV)

Center Number	Atomic Number	Atomic Type	Coordinates (Angstroms) X	Y	Z
1	6	0	5.400928	-1.938585	-0.073046
2	6	0	6.390065	-2.686286	-0.739176
3	7	0	5.647821	-1.391105	1.200845
4	6	0	8.793021	-3.611666	-0.607481
5	6	0	9.734967	-3.519430	0.433215
6	6	0	9.165018	-2.783655	1.506565
7	6	0	4.554354	-0.739076	1.625134
8	6	0	3.554233	-0.853366	0.622421
9	6	0	4.065851	-1.607907	-0.448634
10	6	0	7.638707	-2.922430	-0.133315
11	6	0	3.294828	-1.952432	-1.683437
12	1	0	3.361290	-3.017507	-1.916858
13	1	0	2.242450	-1.690602	-1.552761
14	1	0	3.672524	-1.407449	-2.554099
15	6	0	4.443752	-0.047140	2.940951
16	1	0	4.398143	-0.778391	3.755149
17	1	0	5.310714	0.592892	3.121707
18	1	0	3.538765	0.561453	2.973449
19	6	0	9.038729	-4.301933	-1.911895
20	1	0	10.099229	-4.543243	-2.013701
21	1	0	8.472451	-5.236145	-1.979852
22	1	0	8.740021	-3.681836	-2.759972
23	6	0	9.798324	-2.447427	2.813647
24	1	0	9.301992	-2.984115	3.629008
25	1	0	10.852806	-2.727136	2.806686
26	1	0	9.714984	-1.378328	3.025218
27	7	0	7.918763	-2.429979	1.155702
28	9	0	6.705204	-2.167953	3.241380
29	9	0	7.548354	-0.287694	2.230153
30	5	0	6.965193	-1.549225	2.006764
31	6	0	11.046269	-4.058533	0.427849
32	6	0	2.253793	-0.295490	0.710082
33	7	0	12.124875	-4.502136	0.421820
34	7	0	1.183985	0.163510	0.781001
35	6	0	6.113085	-3.233832	-2.098057
36	6	0	5.731645	-4.570084	-2.269950
37	6	0	6.222650	-2.416698	-3.233540
38	6	0	5.459457	-5.087293	-3.536401
39	1	0	5.622286	-5.216786	-1.404409

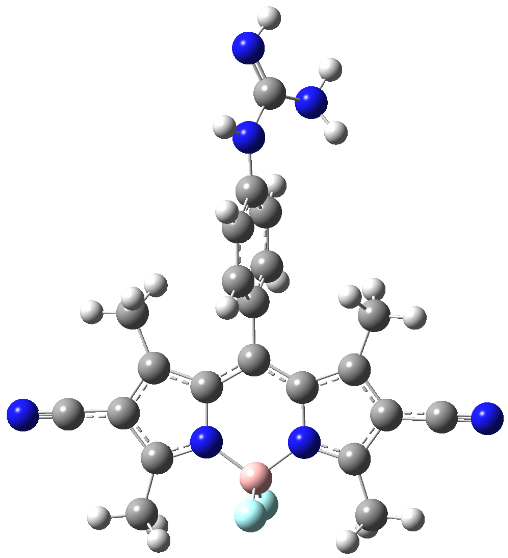

40	6	0	5.982549	-2.930667	-4.502761
41	1	0	6.514506	-1.376166	-3.127679
42	6	0	5.596901	-4.275103	-4.677421
43	1	0	5.126404	-6.113050	-3.628666
44	1	0	6.092333	-2.287634	-5.371212
45	7	0	5.329802	-4.704971	-5.983533
46	1	0	5.082347	-3.983065	-6.647416
47	6	0	5.223326	-5.976277	-6.536401
48	7	0	4.594389	-6.079151	-7.663542
49	7	0	5.788875	-7.011220	-5.816218
50	1	0	4.667213	-7.026455	-8.032257
51	1	0	5.860946	-7.888165	-6.314862
52	1	0	6.611686	-6.800936	-5.264558

Probe 2a
HOMO = -0.23814 Hartree (-6.48 eV)
LUMO = -0.12421 Hartree (-3.38 eV)

Center Number	Atomic Number	Atomic Type	Coordinates (Angstroms) X	Y	Z
1	6	0	5.325248	-2.077006	-0.102032
2	6	0	6.264034	-2.893953	-0.758550
3	7	0	5.651294	-1.433497	1.107679
4	6	0	8.643864	-3.882098	-0.666841
5	6	0	9.657887	-3.692178	0.288908
6	6	0	9.185110	-2.803741	1.292129
7	6	0	4.595701	-0.722382	1.532284
8	6	0	3.543642	-0.886992	0.591111
9	6	0	3.984760	-1.731330	-0.443315
10	6	0	7.549566	-3.085264	-0.218874
11	6	0	3.154820	-2.130214	-1.622288
12	1	0	2.922128	-3.199149	-1.602920
13	1	0	2.212201	-1.578499	-1.620469
14	1	0	3.670914	-1.930532	-2.564502
15	6	0	4.566808	0.075418	2.791256
16	1	0	4.466621	-0.585935	3.659268
17	1	0	5.487736	0.648721	2.916490
18	1	0	3.717202	0.760352	2.784950
19	6	0	8.769236	-4.760589	-1.871250
20	1	0	9.721757	-5.294475	-1.848366
21	1	0	7.962313	-5.496138	-1.918553
22	1	0	8.727795	-4.178298	-2.796461
23	6	0	9.913455	-2.336027	2.505848

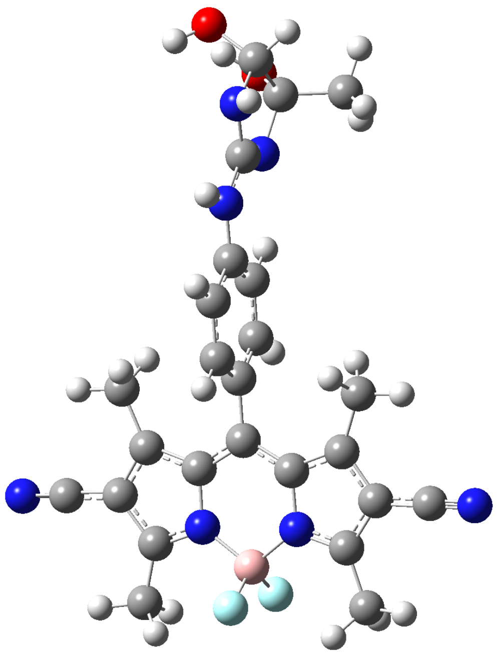

24	1	0	9.499271	-2.805817	3.404508
25	1	0	10.970653	-2.597076	2.437197
26	1	0	9.820136	-1.254153	2.627090
27	7	0	7.928985	-2.450615	0.979461
28	9	0	6.895247	-1.968921	3.120996
29	9	0	7.611311	-0.204608	1.838857
30	5	0	7.035532	-1.489432	1.808305
31	6	0	10.945779	-4.284841	0.275478
32	6	0	2.264665	-0.284013	0.694954
33	7	0	12.005082	-4.772626	0.263848
34	7	0	1.212813	0.212640	0.779413
35	6	0	5.894219	-3.566538	-2.038369
36	6	0	5.228695	-4.797991	-2.036021
37	6	0	6.211080	-2.975210	-3.270646
38	6	0	4.884720	-5.439024	-3.227187
39	1	0	4.971941	-5.270883	-1.092516
40	6	0	5.870880	-3.601858	-4.464317
41	1	0	6.725996	-2.019685	-3.299525
42	6	0	5.208574	-4.844147	-4.458150
43	1	0	4.368162	-6.388696	-3.212807
44	1	0	6.126335	-3.128785	-5.408578
45	7	0	4.907788	-5.418991	-5.701511
46	1	0	5.133374	-4.851989	-6.507993
47	6	0	4.361054	-6.643140	-5.984154
48	7	0	3.848947	-7.487129	-5.153695
49	7	0	4.311144	-6.990913	-7.347246
50	6	0	3.530834	-8.709410	-5.920781
51	6	0	3.672098	-8.281352	-7.436690
52	1	0	5.117691	-6.811356	-7.934114
53	6	0	4.500586	-9.825983	-5.544520
54	8	0	2.219709	-9.162537	-5.645997
55	1	0	4.258293	-8.995504	-8.018626
56	8	0	2.384027	-8.175854	-8.058566
57	1	0	4.416597	-10.038340	-4.474926
58	1	0	4.269191	-10.735560	-6.107760
59	1	0	5.532053	-9.529338	-5.758522
60	1	0	1.641750	-8.763041	-6.320554
61	1	0	2.219846	-7.234110	-8.219950

Probe 2a’
HOMO = -0.24402 Hartree (-6.64 eV)
LUMO = -0.12501 Hartree (-3.40 eV)

Center Number	Atomic Number	Atomic Type	Coordinates (Angstroms) X	Y	Z
1	6	0	5.476152	-2.174165	-0.100184
2	6	0	6.625205	-2.742815	-0.680054
3	7	0	5.510215	-1.665774	1.212393
4	6	0	9.100683	-3.363752	-0.311662
5	6	0	9.922291	-3.147520	0.808667
6	6	0	9.167015	-2.483894	1.813161
7	6	0	4.299701	-1.185142	1.535392
8	6	0	3.438788	-1.368073	0.419793
9	6	0	4.161357	-1.983392	-0.617966
10	6	0	7.833915	-2.809186	0.036874
11	6	0	3.590878	-2.326840	-1.957621
12	1	0	3.508648	-3.409990	-2.089909
13	1	0	2.591640	-1.897820	-2.060167
14	1	0	4.214472	-1.949028	-2.771263
15	6	0	3.959374	-0.582215	2.855473
16	1	0	3.967047	-1.348001	3.638368
17	1	0	4.688114	0.182379	3.136144
18	1	0	2.966201	-0.132036	2.821834
19	6	0	9.543126	-4.048249	-1.566173
20	1	0	10.536574	-4.480224	-1.426780
21	1	0	8.855625	-4.847086	-1.853545
22	1	0	9.593465	-3.348018	-2.405642
23	6	0	9.626771	-2.082073	3.173072
24	1	0	9.242227	-2.778649	3.926600
25	1	0	10.716910	-2.095005	3.222450
26	1	0	9.268255	-1.083584	3.431633
27	7	0	7.925582	-2.289655	1.342776
28	9	0	6.525318	-2.294869	3.324619
29	9	0	7.096843	-0.271272	2.404568
30	5	0	6.767100	-1.610535	2.122034
31	6	0	11.282028	-3.527501	0.938470
32	6	0	2.075405	-0.981228	0.377526
33	7	0	12.400681	-3.839755	1.044462
34	7	0	0.954033	-0.662923	0.341385
35	6	0	6.559310	-3.285266	-2.069031
36	6	0	6.070781	-4.575069	-2.309732
37	6	0	6.986622	-2.511914	-3.157780
38	6	0	6.006010	-5.096266	-3.602273
39	1	0	5.738185	-5.190343	-1.479096

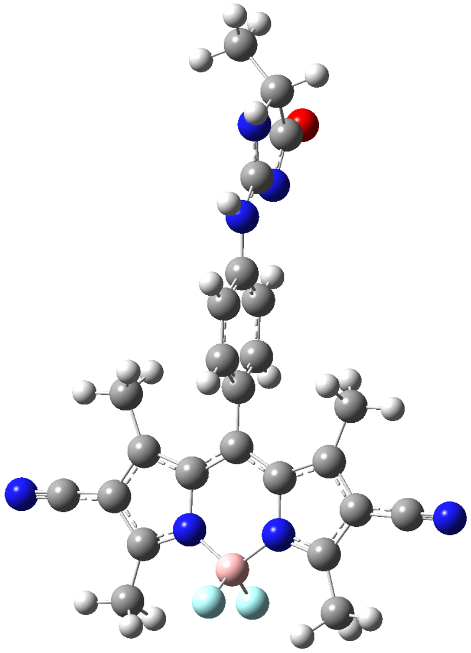

40	6	0	6.918526	-3.016906	-4.452187
41	1	0	7.369835	-1.509066	-2.996973
42	6	0	6.429224	-4.313441	-4.687379
43	1	0	5.629554	-6.094603	-3.773163
44	1	0	7.250096	-2.401898	-5.284097
45	7	0	6.402296	-4.744629	-6.030980
46	1	0	6.738057	-4.060987	-6.698430
47	6	0	5.989217	-5.917856	-6.565445
48	7	0	5.455672	-6.930722	-5.881193
49	7	0	6.079480	-6.108538	-7.911436
50	6	0	5.230884	-7.935479	-6.796475
51	6	0	5.690352	-7.488020	-8.200256
52	1	0	6.732324	-5.600965	-8.494045
53	8	0	4.730801	-9.033151	-6.550316
54	1	0	6.574297	-8.076540	-8.479023
55	6	0	4.612453	-7.618826	-9.275567
56	1	0	3.725785	-7.035602	-9.009755
57	1	0	4.326167	-8.669185	-9.377882
58	1	0	4.987761	-7.267103	-10.240763

VII. Supplementary Figure S3: Synthesis of Probe 1a

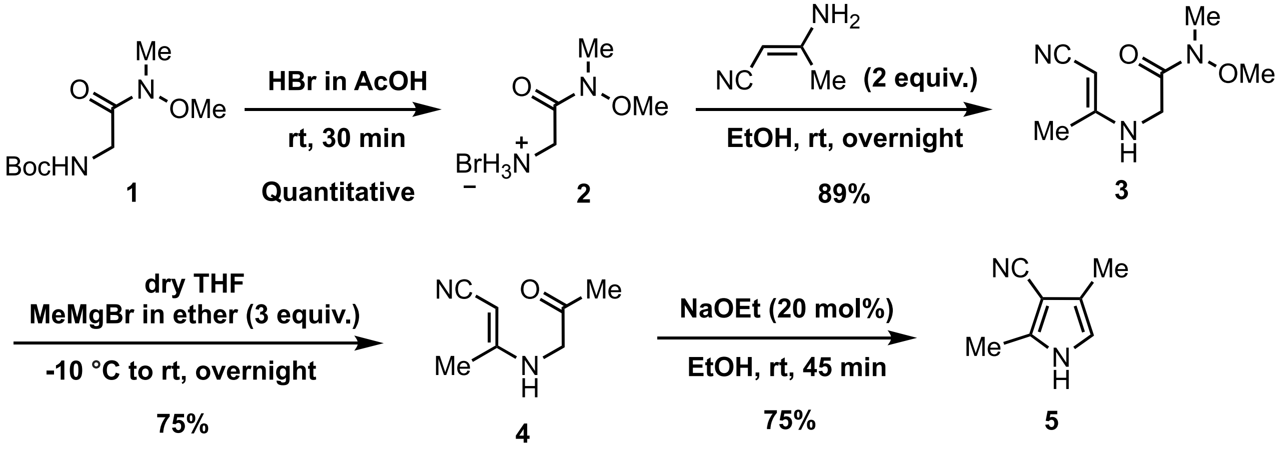

Step-I: *tert*-Butyl (2-(methoxy(methyl)amino)-2-oxoethyl)carbamate **1** (6.54 g, 30 mmol, 1 equiv.) was taken in a 100 mL round bottom flask and 25% HBr in AcOH (25 mL) was added to it dropwise at room temperature while stirring. The reaction mixture was stirred further for 30 minutes. After completion, diethyl ether (Et_2O) was added to the mixture. A white solid precipitate formed, which was collected by filtration and washed with Et_2O several times to afford the salt 2-(methoxy(methyl)amino)-2-oxoethan-1-aminium bromide **2** (5.97 g, quantitative yield). The product was dried over high vacuum overnight and used for the next step without further purification. **^{1}H NMR** (400 MHz, D_2O) δ (ppm): 4.06 (s, 2H), 3.75 (s, 3H), 3.22 (s, 3H).

Step-II: In a 500 mL round bottom flask, 2-(methoxy(methyl)amino)-2-oxoethan-1-aminium bromide salt **2** (5.97 g, 30 mmol, 1 equiv.) and 3-aminocrotononitrile (4.92 g, 60 mmol, 2 equiv.) were taken. Then, the solids were dissolved in ethanol (400 mL) at room temperature and stirred overnight at room temperature. After completion, the solvent was evaporated in vacuo and the product was isolated via silica gel column chromatography by using 50% ethyl acetate/hexane as eluent. The desired product (*E*)-2-((1-cyanoprop-1-en-2-yl)amino)-*N*-methoxy-*N*-methylacetamide **3** (4.89 g, 89% yield) was obtained as pale white solid. Analytical TLC, 1:1 ethyl acetate:hexane eluent, R_f = 0.4. **^{1}H NMR** (400 MHz, $CDCl_3$) δ (ppm): 5.38 (br s, 1H), 3.73 (s, 2H), 3.67 – 3.65 (m, 4H), 3.14 (s, 3H), 2.07 (s, 3H).

Step-III: In a 250 mL two necked round bottom flask, (*E*)-2-((1-cyanoprop-1-en-2-yl)amino)-*N*-methoxy-*N*-methylacetamide **3** (3.6g, 20 mmol, 1 equiv.) and the flask was put under inert conditions by evacuating and backfilled with nitrogen (three times). Then, dry tetrahydrofuran (THF, 200 mL) was added to the flask at room temperature under N_2. The system was cooled to -10 °C and to the solution, MeMgBr (3 M in diethyl ether) (20 mL, 3 equiv.) was added in a dropwise manner over 30 mins and left the reaction mixture stirred for overnight at room temperature. After completion, the yellowish orange reaction mixture was quenched with minimum amount of saturated ammonium chloride solution (until the precipitate formation stopped). The organic layer was removed, and water (H_2O, 80 mL) was added to the solid precipitate, which was extracted with dichloromethane (3 x 50 mL). The accumulated organic layers were concentrated in vacuo and the crude product was purified by column chromatography using 50% ethyl acetate/hexane as eluent. The desired product (*E*)-3-((2-oxopropyl)amino)but-2-enenitrile **4** was obtained as pale-yellow solid (2.1 g, 75% yield). Analytical TLC, 1:1 ethyl acetate:hexane eluent, R_f = 0.5. **^{1}H NMR** (400 MHz, $CDCl_3$) δ (ppm): 5.29 (br s, 1H), 3.78 (d, *J* = 4.3 Hz, 2H), 3.64 (s, 1H), 2.17 (s, 3H), 2.06 (s, 3H).

Step-IV: In a 100 mL round bottom flask, (*E*)-3-((2-oxopropyl)amino)but-2-enenitrile **4** (2.1 g, 15 mmol, 1 equiv.) was dissolved in ethanol (60 mL) while stirring at room temperature. Then, sodium ethoxide (205 mg, 3 mmol, 0.2 equiv.) was added to the solution at once the reaction mixture was further stirred for 45 minutes at room temperature. After completion, the solvent was evaporated in vacuo and the crude product was isolated in silica gel column chromatography by using 15% ethyl acetate/hexane as eluent. The desired product 2,4-dimethyl-1*H*-pyrrole-3-carbonitrile **5** (1.35 g, 75% yield) was obtained as yellow solid. Analytical TLC, 3:7 ethyl acetate:hexane eluent, R_f = 0.6. **^{1}H NMR** (400 MHz, $CDCl_3$) δ (ppm): 8.56 (brs, 1H), 6.38 (d, *J* = 1.2 Hz, 1H), 2.37 (s, 3H), 2.12 (s, 3H). **^{13}C {^{1}H} NMR** (101 MHz, $CDCl_3$) δ (ppm): 137.2, 121.8, 117.4, 114.9, 92.4, 12.4, 10.7. Spectra matches literature reports.[3]

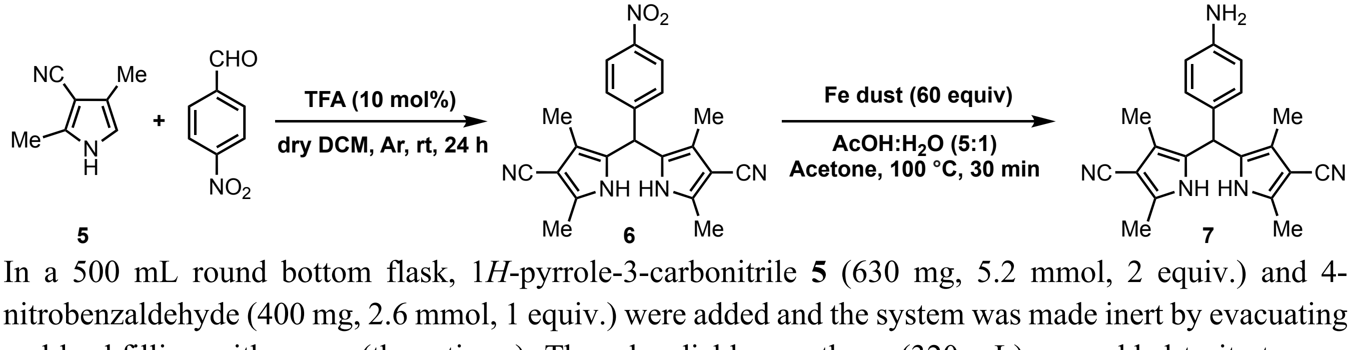

In a 500 mL round bottom flask, 1*H*-pyrrole-3-carbonitrile **5** (630 mg, 5.2 mmol, 2 equiv.) and 4-nitrobenzaldehyde (400 mg, 2.6 mmol, 1 equiv.) were added and the system was made inert by evacuating and backfilling with argon (three times). Then dry dichloromethane (320 mL) was added to it at room

temperature under argon atmosphere and stirred for 24 h at the same temperature. After complete consumption of the starting materials via TLC, solvent was evaporated under reduced pressure then washed and triturated with diethyl ether (2 x 100 mL). The crude pale orange solid product 5,5'-((4-nitrophenyl)methylene)bis(2,4-dimethyl-1*H*-pyrrole-3-carbonitrile) **6** was dried in vacuum for 3-4 hours and used for the next step without further purification. Analytical TLC, 1:1 ethyl acetate:hexane eluent, $R_f = 0.4$. **^{1}H NMR** (400 MHz, DMSO-d_6) δ (ppm): 10.85 (s, 2H), 8.21 (d, $J = 8.8$ Hz, 2H), 7.35 (d, $J = 8.3$ Hz, 2H), 5.65 (s, 1H), 2.25 (s, 6H), 1.80 (s, 6H).

In a 250 mL pressure tube, crude nitrophenyl-pyrrole adduct **6** (968 mg, 2.6 mmol, 1 equiv.) was dissolved in 130 mL acetone while stirring. After that, Fe dust (8.7 g, 156 mmol, 60 equiv.) was added to the solution at room temperature followed by water (2.6 ml), then acetic acid (13 ml) was added to the reaction mixture with vigorous stirring. The reaction mixture was heated at 100 °C for 30 mins. After completion, the reaction mixture was cooled to room temperature and filtered through 230–400 mesh silica bed followed by washing with acetone. The filtrate was evaporated and diluted with water (80 mL). The aqueous layer was extracted with ethyl acetate (3 x 50 mL), then the organic layer was washed with saturated sodium bicarbonate solution (80 mL) and brine (50 mL). Then, the accumulated organic layer was concentrated in vacuum and the crude product was purified through silica gel column chromatography using 70% ethyl acetate/hexane as eluent. The desired product 5,5'-((4-aminophenyl)methylene)bis(2,4-dimethyl-1*H*-pyrrole-3-carbonitrile) **7** (767 mg, 86% yield) was obtained as pale-yellow solid. Analytical TLC, 1:1 ethyl acetate:hexane eluent, $R_f = 0.2$. **^{1}H NMR** (400 MHz, DMSO-d_6) δ (ppm): 10.77 (br s, 2H), 6.77 (d, $J = 8.4$ Hz, 2H), 6.60 (d, $J = 8.5$ Hz, 2H), 5.91 (br s, 2H), 5.26 (s, 1H), 2.23 (s, 6H), 1.78 (s, 6H).

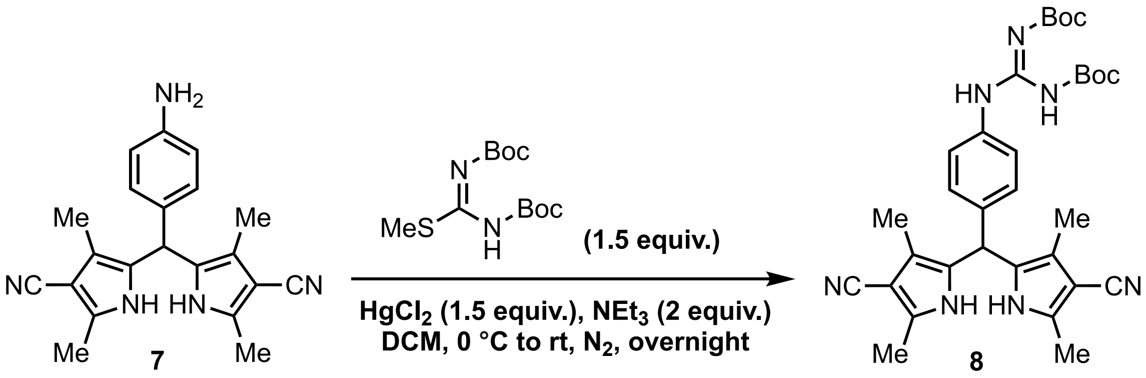

In a 250 mL round bottom flask, reduced aniline substrate **7** (767 mg, 2.23 mmol, 1 equiv.), 1,3-bis(tert-butoxycarbonyl)-2-methyl-2-thiopseudourea (973 mg, 3.35 mmol, 1.5 equiv.) and were taken and made the system inert by evacuating and backfilled with nitrogen (three times). Then, dry dichloromethane (130 mL) was added to the flask at room temperature under nitrogen atmosphere. The system was further cooled to 0 °C and to the solution, triethylamine (0.6 mL, 4.46 mmol, 2 equiv.) was added in a dropwise manner and stirred for 5-10 mins at the same temperature. After that, mercury(II) chloride (908 mg, 3.35 mmol, 1.5 equiv.) was added at once to the reaction mixture under positive nitrogen pressure at 0 °C. The solution was gradually warmed to room temperature and stirred overnight. After completion, the reaction mixture was filtered through celite which was washed with DCM. Then the organic layer was washed with water (80 mL) and brine (50 mL). The accumulated organic layer was concentrated in vacuum and the crude

product was purified through silica gel column chromatography using 40% ethyl acetate/hexane as eluent. The desired Boc-protected aniline-pyrrole product **8** (1.17 g, 91% yield) was obtained as pale-orange solid. Analytical TLC, 7:3 ethyl acetate:hexane eluent, $R_f = 0.2$. **^{1}H NMR** (400 MHz, DMSO-d_6) δ (ppm): 11.39 (s, 1H), 10.81 (s, 2H), 9.98 (s, 1H), 7.51 (d, J = 8.7 Hz, 2H), 7.04 (d, J = 8.6 Hz, 2H), 5.43 (s, 1H), 2.25 (s, 6H), 1.81 (s, 6H), 1.50 (s, 9H), 1.39 (s, 9H). **^{13}C {^{1}H} NMR** (101 MHz, DMSO-d_6) δ (ppm): 162.6, 152.8, 152.1, 137.3, 136.0, 135.3, 128.3, 126.9, 122.8, 117.1, 116.5, 91.2, 83.4, 78.8, 27.9, 27.6, 11.9, 9.5.

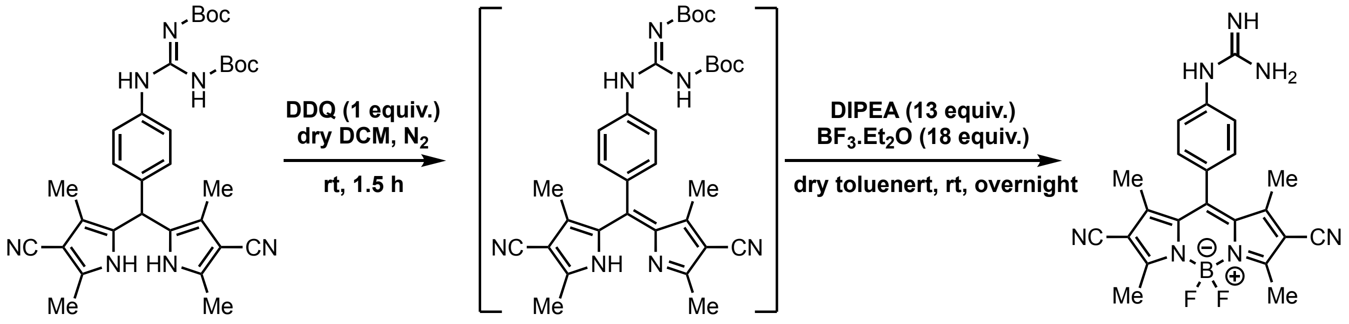

In a 50 mL round bottom flask, Boc-protected substrate **8** (58 mg, 0.1 mmol, 1 equiv.) was taken and made the system inert by evacuating and backfilled with nitrogen (three times). Dry DCM (10 mL) was added to the flask at room temperature under nitrogen atmosphere. Then, the solution was again degassed with nitrogen by purging through needle for 15 mins. After that, 2,3-Dichloro-5,6-dicyano-1,4-benzoquinone (DDQ, 23 mg, 0.1 mmol, 1 equiv.) was added to the solution at once under positive nitrogen pressure and left the solution stirred at the same temperature for 2 hours. After completion, the reaction mixture was quenched with brine (8 mL) and the aqueous layer was extracted with DCM (3 x 5 mL). The accumulated organic layer was evaporated under reduced pressure and the crude product was passed through basic alumina column using DCM as eluent to afford an orange-solid intermediate.

Next, the orange-solid intermediate was taken in a 50 mL round bottom flask, and made the system inert by evacuating and backfilled with nitrogen (three times). Dry toluene (5 mL) was added to the flask at room temperature under nitrogen atmosphere. Then, *N,N*-diisopropylethylamine (0.23 mL, 1.3 mmol, 13 equiv.) was added under positive nitrogen pressure and stirred for 10-15 mins at the same temperature. After that, boron trifluoride etherate solution (0.2 ml, 1.8 mmol, 18 equiv.) was added in a dropwise manner under N_2 and left the solution stirred at the same temperature for overnight. After completion, the reaction mixture was quenched with brine (15 mL) and the aqueous layer was extracted with ethyl acetate (3 x 15 mL) then DCM (2 x 10 mL). The accumulated organic layer was evaporated under reduced pressure and the crude product was initially purified through silica gel column chromatography using 20% methanol/DCM as eluent. After evaporation of the solvent, the semi crude product was redissolved in MeOH:ACN (1:1) and finally purified through HPLC. The HPLC method used a non-linear gradient 10-60% of solvent B over 40 mins at RT with a flow rate of 1 mL·min^{-1}. The eluent was monitored by absorbance at 220 nm. The desired MGO probe **1a** (1.5 mg, 3.4% yield) was obtained as orange solid. **^{1}H NMR** (400 MHz, DMSO-d_6) δ (ppm): 10.32 (s, 1H), 7.76 (s, 3H), 7.52-7.46 (m, 4H), 2.65 (s, 6H), 1.62 (s, 6H). **^{13}C {^{1}H} NMR** (201 MHz, DMSO-d_6) δ (ppm): 159.0, 155.4, 149.4, 147.1, 137.7, 131.0, 129.0 (CH), 128.6, 123.6 (CH), 113.5, 105.5, 14.1 (CH_3), 13.6 (CH_3). **^{11}B NMR** (128 MHz, DMSO-d_6) δ (ppm):

0.53, 0.29, 0.04. **^{19}F NMR** (376 MHz, DMSO-d_6) δ (ppm): -142.73, -142.81, -142.90, -142.98. **HRMS (ESI) m/z:** $[M+H]^+$ Calcd for $C_{22}H_{21}BF_2N_7$ 432.1914; Found 432.1915. **Retention Time:** (0-80% solvent B over 30 minutes) 18.5 minutes.

HPLC of 1a

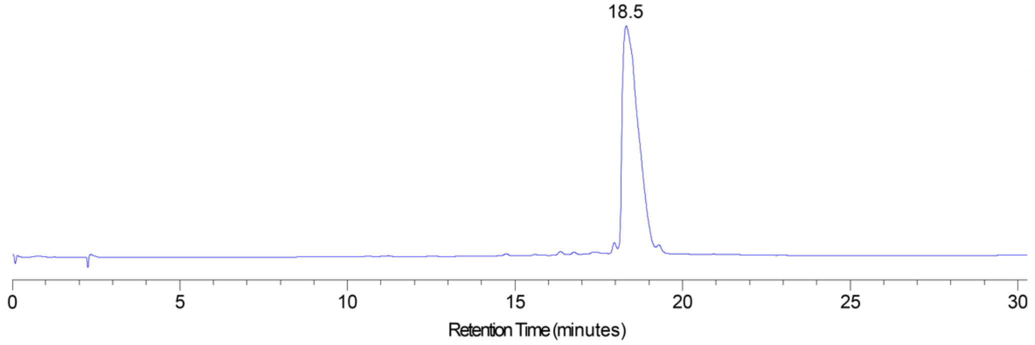

HRMS of 1a

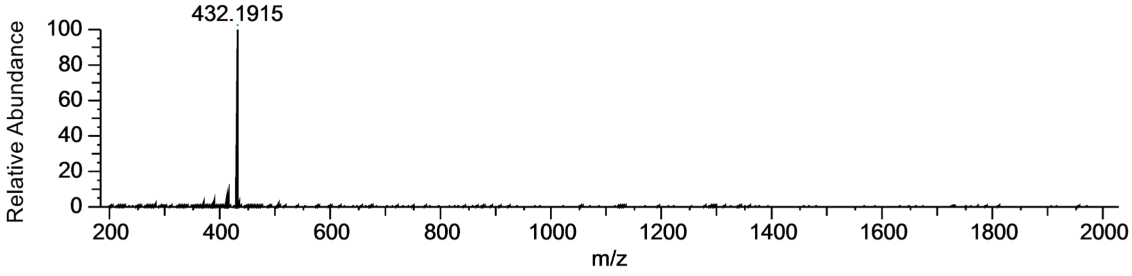

Synthesis of Probe 2a and 2a'

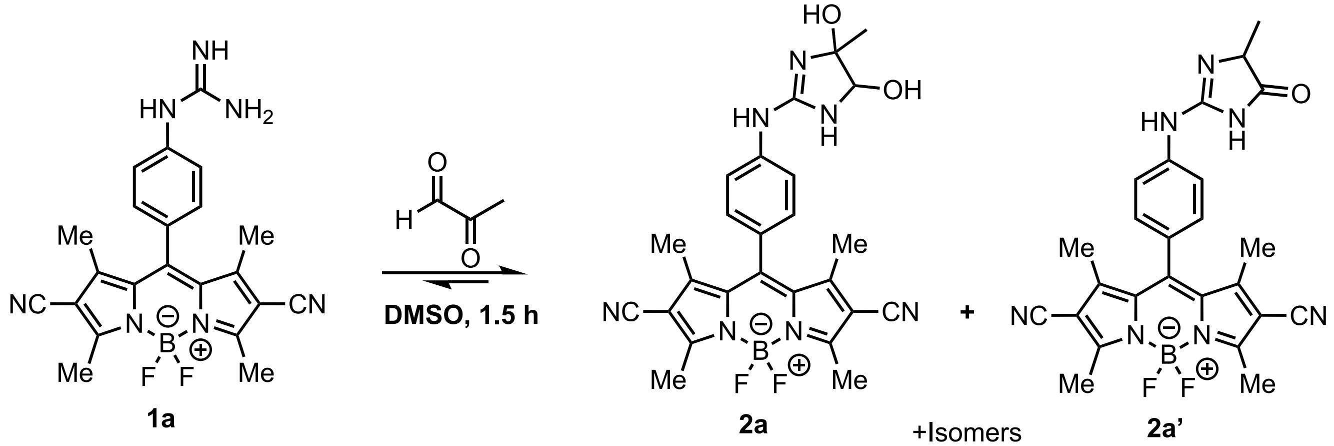

To a stirred solution of probe **1a** (1 equiv.) in DMSO, MGO (10 equiv.) was added, and the reaction was stirred at RT for 1.5 h. Crude mixture was analyzed via HPLC and HRMS. Attempts at product isolation were unsuccessful, likely due to reversibility. The dehydrate product was also observed as a minor product in the reaction mixture.

Retention Time: (0-80% solvent B over 30 minutes) 17.5-18 minutes.
2a: HRMS (ESI) m/z: $[M+H]^+$ Calcd for $C_{25}H_{24}BF_2N_7O_2$: 504.2125; Found 504.2126.
2a': HRMS (ESI) m/z: $[M+H]^+$ Calcd for $C_{25}H_{23}BF_2N_7O$: 482.2020; Found 482.2022.

HPLC of Probe 2a and 2a'

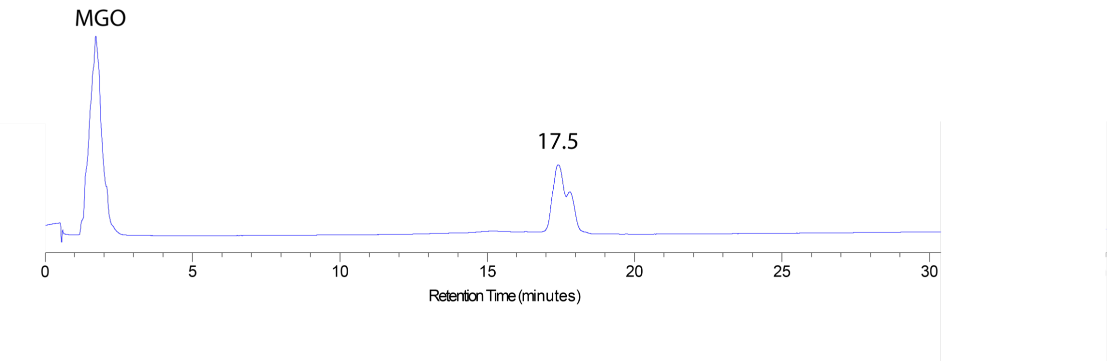

HRMS of 2a

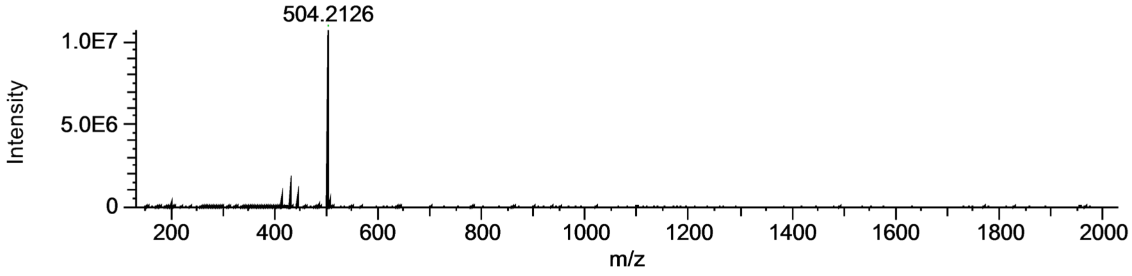

HRMS of 2a'

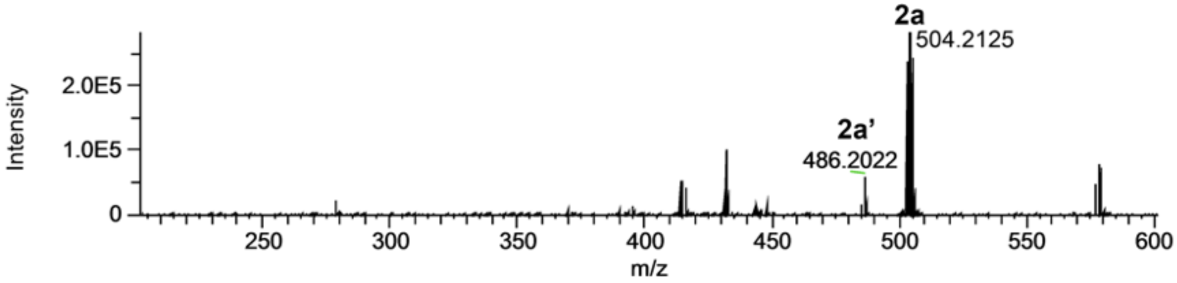

VIII. Supplementary Figure S4: Quantum yield determination of probes 1a and 2a. Quantum yield was calculated using the area under the curve of fluorescence versus absorption. Absorption was measured with a Cary 3500 UV-Vis utilizing the samples at using four separate concentrations with values being divided by ten for accurate analysis. Fluorescence area was measured with a Cary Eclipse fluorimeter using the same samples above with a 10X dilution. All measurements were run in triplicate. Quantum yields of probe **1a** and **2a** were determined using Cy2 as a reference compound. The following equation was used to calculate quantum yield:

$$\Phi = \Phi_r \times \frac{m}{m_r} \times (\frac{n}{n_r})^2$$

Φ is the quantum yield; m is the slope of the line described above; n is the refractive index of the solvent. Subscript r denotes the appropriate values for the reference (Cy2).

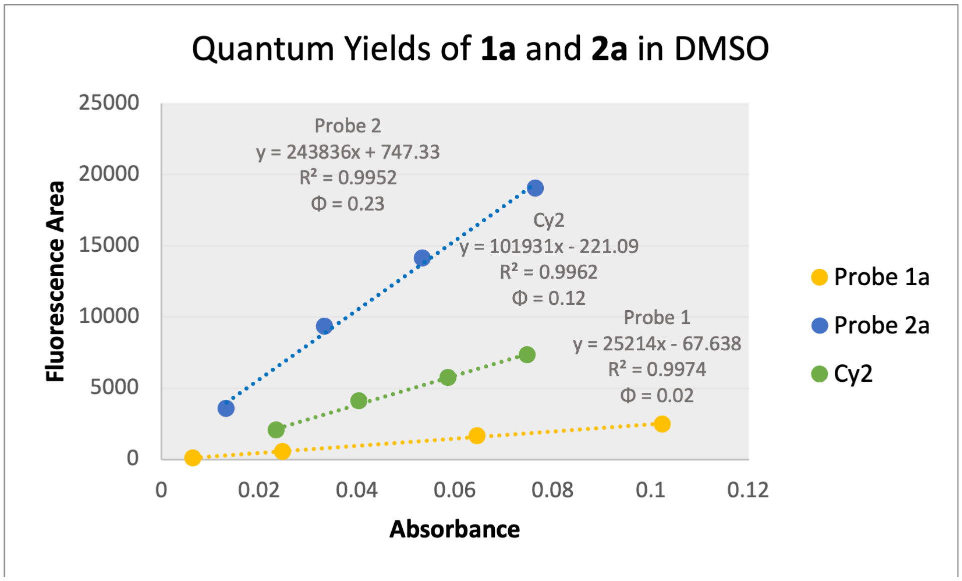

IX: Supplementary Figure S5: Probe 1a Characteristics and Chemoselectivity

Absorbance and Emission of 2a in PBS buffer (10 mM, pH 7.4)

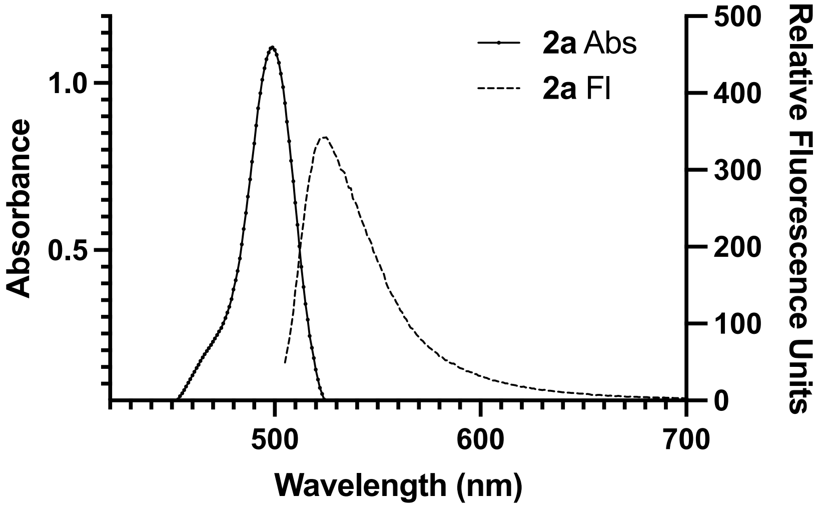

Chemoselectivity:

Probe **1a** (1 equiv.) was incubated with 100 equiv. of each compound in DMSO for 2 hours, then fluorescence intensity was recorded. This was repeated in triplicate. Error bars represent mean ± standard deviation. *Nitric oxide was produced by S-nitroso-N-acetylpenicillamine (SNAP).

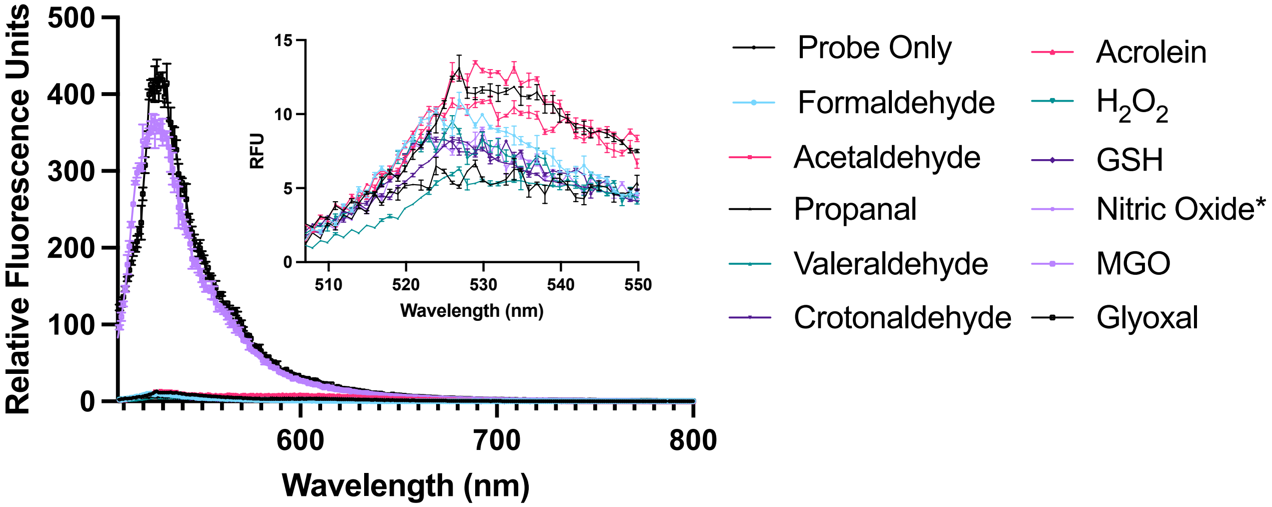

Competition Study:

Probe **1a** (1 equiv.) was incubated with 100 equiv. of each compound alone (left) or with 100 equiv. of MGO (right) in DMSO for 2 hours, then fluorescence intensity was recorded. This was repeated in triplicate. Error bars represent mean ± standard deviation. *Nitric oxide was produced by S-nitroso-N-acetylpenicillamine (SNAP). % RFU calculated as the peak RFU value for given trial divided by the peak RFU value for 100 equiv. MGO alone (x100). Experiments were repeated in triplicate.

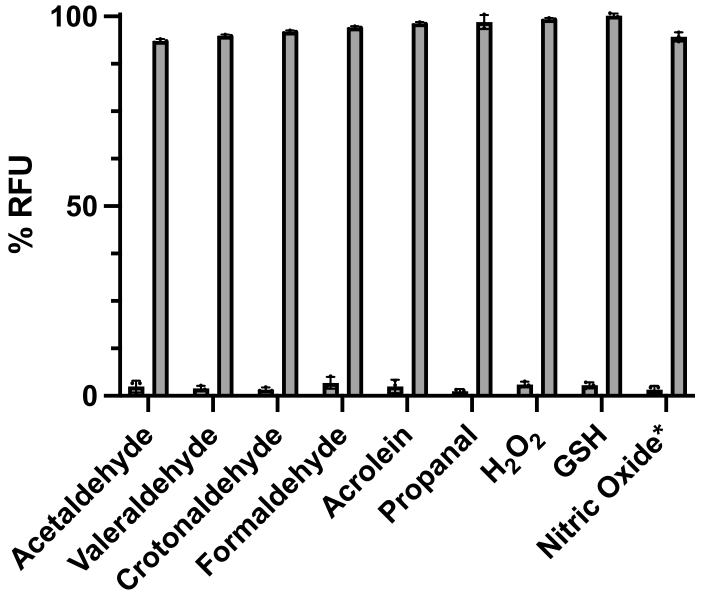

In Vitro Reversibility:
Probe **1a** was incubated with alternating and increasing amounts of either MGO or aminoguanidine (AG) in PBS buffer (10 mM, pH 7.4) and analyzed via fluorimeter. A) probe only. B) probe + 10 equiv. MGO for 1 hour then measurement. C) Previous + 20 equiv. AG for 1 hour then measurement. D) Previous + 40 equiv. MGO for 1 hour then measurement. E) Previous + 60 equiv. AG for 1 hour then measurement. F) Previous + 100 equiv. MGO for 1 hour then measurement. Experiment was repeated 3 separate times. HRMS was acquired for points A-D to verify probe reversability.

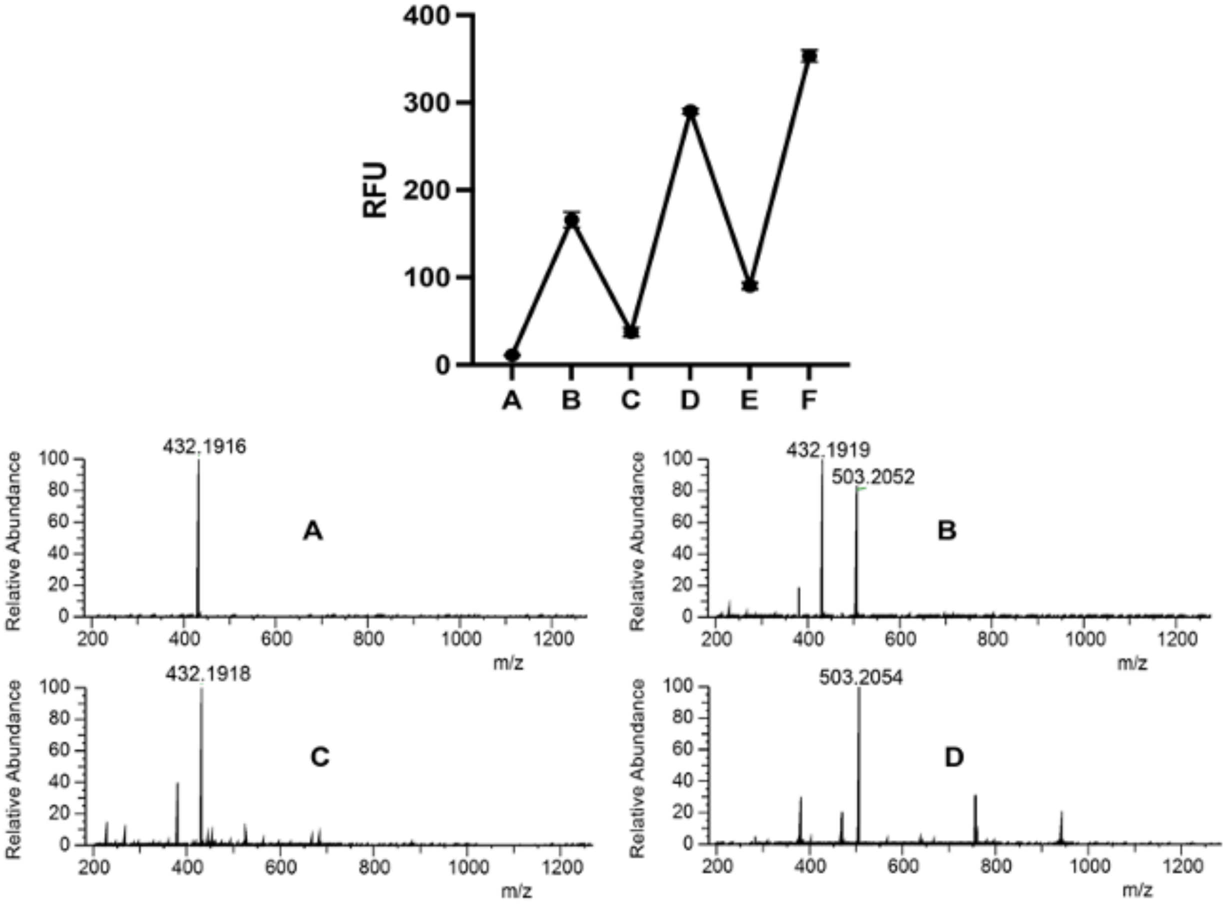

Kinetics:
The rate of dihydroxyimidazolidine formation was determined upon reaction of sensor **1a** (10 μM, 1 equiv.) with MGO (1 mM, 100 equiv.) in PBS buffer (pH 7.4) at 37 °C. Fluorescence intensity was monitored over 4 hours with measurements collected every 5 minutes using a microwell plate reader. The data were fitted to a plateau followed by one-phase association model in GraphPad Prism to account for the observed lag phase, and the pseudo-first-order rate constant k_{obs} was extracted from the fit. The experiment was performed in triplicate. The lag phase of approximately 15 minutes is consistent with the two-step mechanism established for MGO-guanidine condensation, in which an initial reversible imine intermediate accumulates prior to cyclization to the fluorescent dihydroxyimidazolidine product.[4] The electron-withdrawing BODIPY fluorophore reduces the pKa of the guanidinium group relative to free arginine residues (pKa ~13.8), increasing the fraction of neutral reactive guanidine at physiological pH

and thereby accelerating the condensation reaction, consistent with the fluorophore-promoted deprotonation mechanism demonstrated for analogous aryl guanidine fluorescent probes for glyoxal species detection.[5]

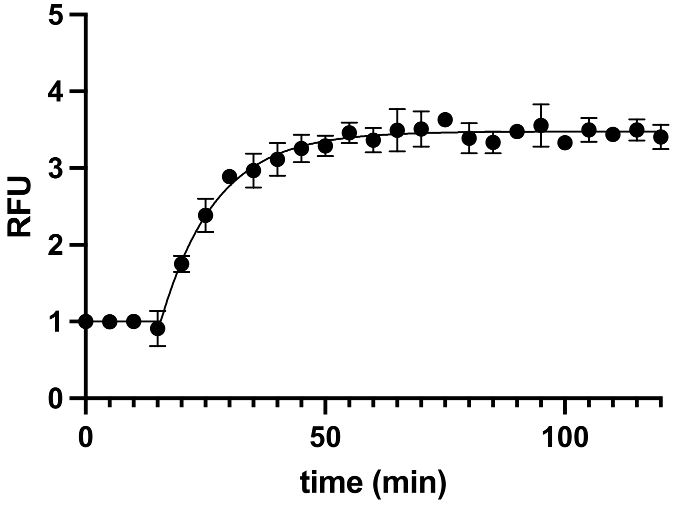

X: Supplementary Figure S6: Flow Cytometry Cell Death

Flow Cytometry: Cells were grown in 60 x 15 mm Nunclon™ dishes. 10 mM Stock solutions of probe **1a** (20 µM) and drugs methylglyoxal (MGO, 250 µM), curcumin (10 µM), resveratrol (Res, 10 µM), and Hesperetin (Hep, 50 µM) were prepared in DMSO before being diluted to the final desired concentration in 4 mL of media. Cells were placed in the incubator for treatment of compounds for 4 hours, except for probe **1a** and Res + Hep, which were dosed for 24 hours. Cells were then detached with trypsin, collected, washed, and stained using Annexin V/PI following the manufacturer's protocol. To avoid fluorescent crosstalk, commercially available Annexin V (AV) conjugated to Pacific Blue (PB) was used to determined apoptosis with the exception of cells dosed with curcumin, which were stained with AV conjugated to FITC to avoid fluorescent crosstalk. Propidium Iodide (PI) was used to determine necrosis within the cellular populations. Cells were analyzed via flow cytometry within 1 hour to quantify cell death. A V470/15 laser was used to detect AV-PB (apoptosis) while a B-710/50 laser was used to detect PI (necrosis). FlowJo software was used to analyze cytometry data. Gating was applied based on AV/PI/Unstained control samples run for each batch of samples; gating slightly varied between samples as seen in representative graphs below. All compounds were analyzed in triplicate and were normalized to naïve cell viability (in triplicate). The dashed line represents 85% cell viability.

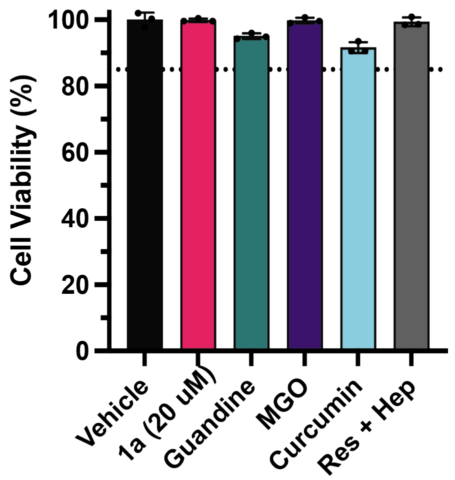

Representative Flow Cytometry of each Compound in HeLa cells

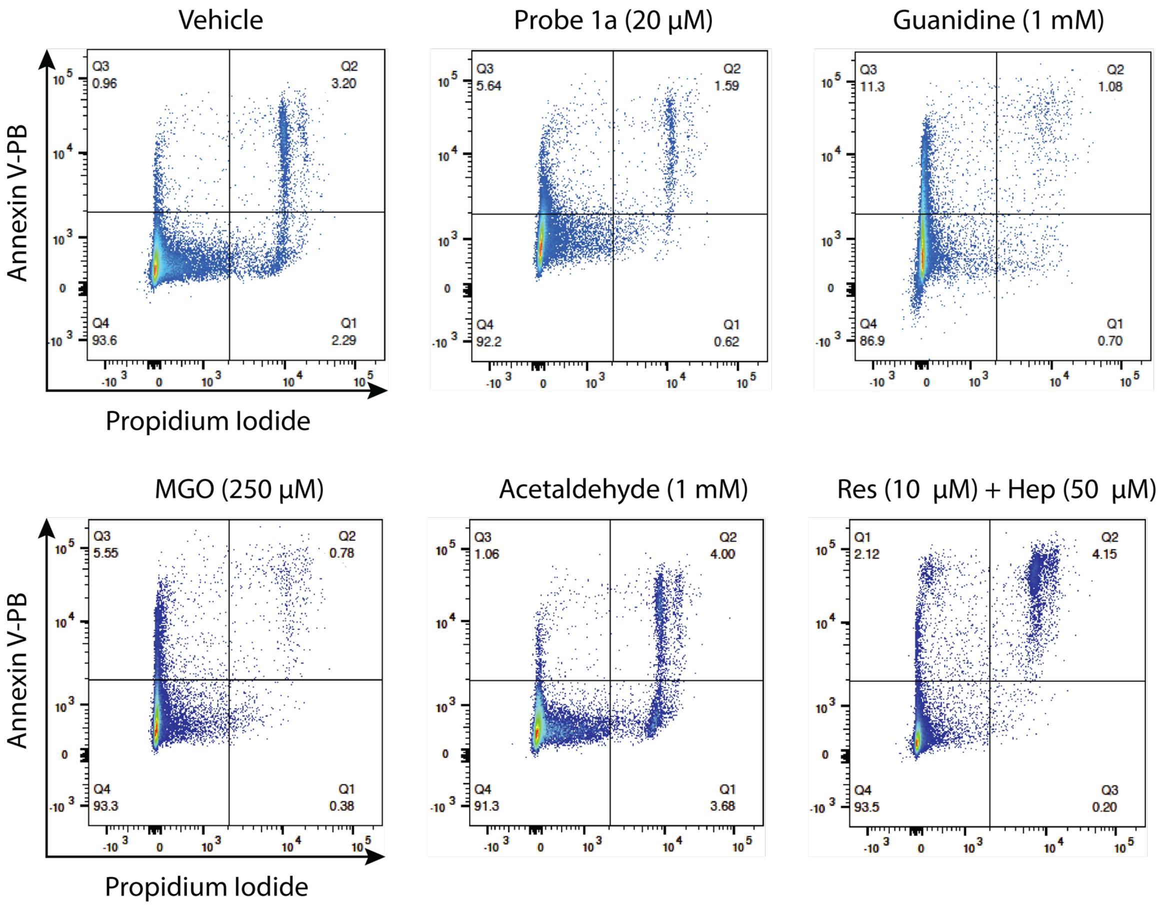

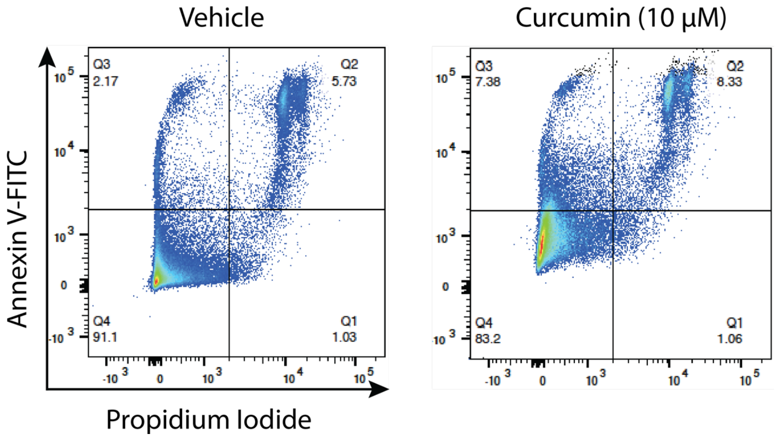

XI: Supplementary Figure S7: Localization of Probe 1a HeLa cells

HeLa cells were plated in an IBIDI 8-well glass bottom chamber at a density of 25,000 cells per well in media and allowed to adhere overnight at 37 °C, 5% CO_2. A fresh 10 mM stock solution of probe **1a** was prepared in DMSO on the day of experimentation. Working solutions of all compounds in media were prepared the day of experimentation. Media was removed from wells and 200 µL of 10 µM **1a** was added to desired wells and placed in an incubator for 1.5 hours. Probe media was removed, and cells were washed with 200 µL of PBS for 5 minutes in the incubator (repeated 3x). Cells were then stained with either LysoTracker® RED DND-99 (ThermoFischer, L7528) or Mitrotracker® FM (ThermoFischer, M22425) was added according to manufacturer protocol, and cells were incubated for 20 minutes before staining media was removed and cells were washed with 200 µL of PBS for 5 minutes (repeated 3 times). Cells were stained with Hoechst 33342 according to the manufacturer's protocol for 5 minutes. Staining media was removed, and cells were washed with 200 µL of PBS for 5 minutes (repeated 3 times). PBS was replaced with 200 µL of fresh media followed by immediate imaging. Five images were captured for each well. Colocalization analysis for Pearsons' R (R) and Mander's Colocalization Coefficient (MCC) were conducted using the EzColocalization Plugin for ImageJ.[6] The scale bar represents 10 µm.

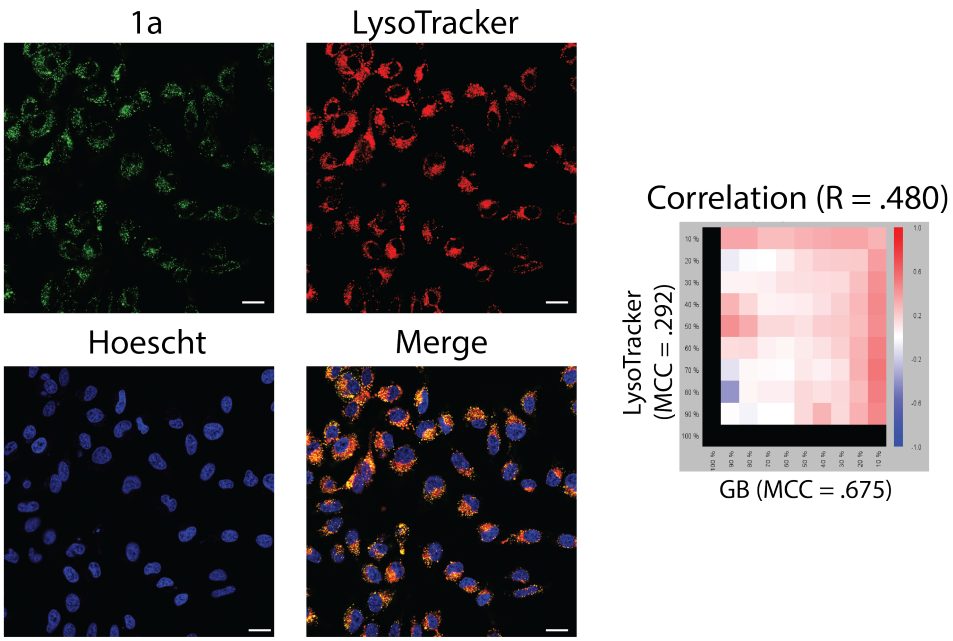
1a
LysoTracker
Hoescht
Merge
Correlation (R = .480)
LysoTracker
(MCC = .292)
GB (MCC = .675)

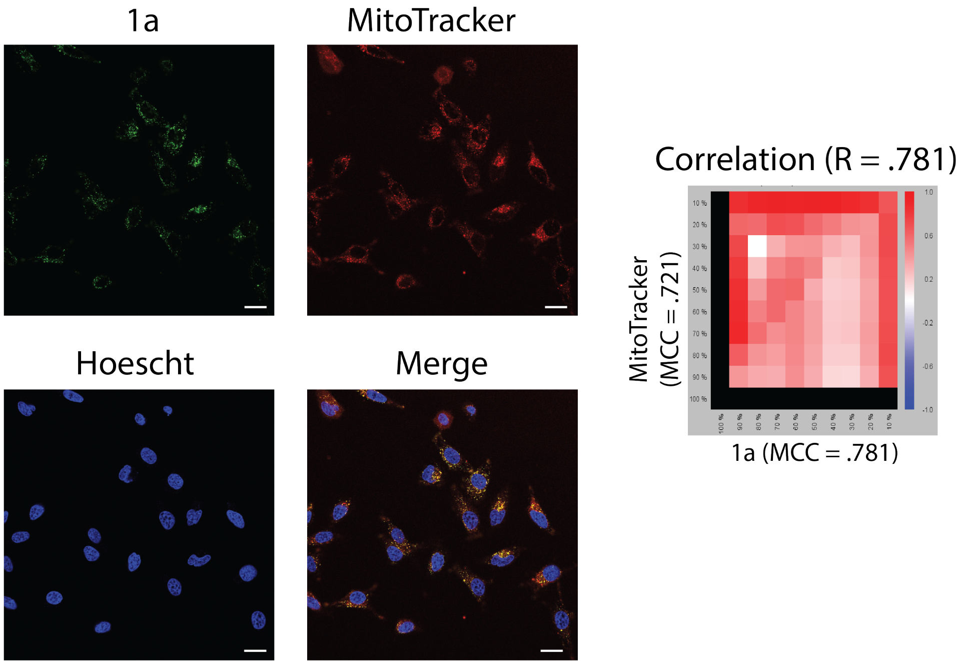
1a
MitoTracker
Hoescht
Merge
Correlation (R = .781)
MitoTracker
(MCC = .721)
1a (MCC = .781)

XII: Supplementary Figure S8: Imaging of HeLa cells with Exogenous MGO

HeLa ells were plated in an IBIDI 8-well glass bottom chamber at a density of 25,000 cells per well in media and allowed to adhere overnight at 37 °C, 5% CO_2. A fresh 10 mM stock solution of probe **1a** was prepared in DMSO on the day of experimentation. Working solution of 50 mM MGO was prepared the day of experimentation. Media was removed from wells and 200 µL of the final concentration of MGO in media (0, 10, 50, 100, or 250 µM) was added to desired wells. Cells were placed in an incubator for 15 minutes, followed by removal of the dosage media. Dosage media was removed, and 200 µL of 20 µM of probe **1a** in media was added to wells. Cells were incubated for 90 minutes. Next, media was removed and washed with 200 µL of PBS for 5 minutes in the incubator (washing repeated 3x total). Cells were stained with Hoechst 33342 according to the manufacturer's protocol for 5 minutes. Staining media was removed, and cells were washed with 200 µL of PBS for 5 minutes (repeated 3 times). PBS was replaced with 200 µL of fresh media followed by immediate imaging. Five images were captured for each well. This process was repeated in triplicate on separate days with different cell passage numbers. 10 cells were analyzed per image to determine average ROI for each image. An outlier test was performed to remove any extraneous data points before statistical analysis was conducted via Student's T-test (n = 15). Error bars represent standard deviation. The layout for this experiment is depicted below where x represents a blank well:

0 µM	10 µM	50 µM	100 µM
250 µM	**X**	**X**	**X**

XIII: Supplementary Figure S9: Endogenous Imaging of MGO in HeLa cells

HeLa cells were plated in an IBIDI 8-well glass bottom chamber at a density of 25,000 cells per well in media and allowed to adhere overnight at 37 °C, 5% CO_2. A fresh 10 mM stock solution of probe **1a** was prepared in DMSO on the day of experimentation. Working solutions of drugs prepared the day of experimentation. Media was removed from wells and 200 μL of the final concentration in media of resveratrol and hesperetin (Res and Hep, 10 μM and 50 μM), curcumin (Cur, 10 μM), or curcumin with MGO (Cur and MGO, 10 μM and 50 μM) was added to desired wells. Cells were placed in an incubator for 4 hours, followed by removal of the dosage media. Res and Hep were dosed 20 hours ahead of time to obtain a final dosing time of 24 hours. Dosage media was removed, and 200 μL of 20 μM of probe **1a** in media was added to wells. Cells were incubated for 90 minutes. Probe media was removed, and cells were washed with 200 μL of PBS for 5 minutes in the incubator (repeated 3x). Cells were then stained with CellMask™ Membrane Stain (ThermoFischer, C10046) in media according to manufacturer protocol, and cells were incubated for 20 minutes before staining media was removed and cells were washed with 200 μL of PBS for 5 minutes (repeated 3 times). Next, media was removed and washed with 200 μL of PBS for 5 minutes in the incubator (washing repeated 3x total). Cells were stained with Hoechst 33342 according to the manufacturer's protocol for 5 minutes. Staining media was removed, and cells were washed with 200 μL of PBS for 5 minutes (repeated 3 times). PBS was replaced with 200 μL of fresh media followed by immediate imaging. Five images were captured for each well. This process was repeated in triplicate on separate days with different cell passage numbers. 10 cells were analyzed per image to determine average ROI for each image. An outlier test was performed to remove any extraneous data points before statistical analysis was conducted via Student's T-test (n = 15). Error bars represent standard deviation. The layout for this experiment is depicted below where x represents a blank well:

Blank	**1a**	1a + Res + Hep	**X**
**X**	**1a** + Cur	**1a +** Cur + MGO	**X**

XIV: Supplementary Figure S10: Probe 1a Reactivity with Cellular Modifiers

Probe **1a** (1 equiv.) was incubated with 10 equiv. of resveratrol, hesperetin, or curcumin in PBS (pH 7.4) and stirred at 37 °C for 24 hours. Reactions were analyzed via HPLC with 0-80% B over 30 minutes. No reaction was observed between probe **1a** and any of the cellular modifiers.

Probe 1a + curcumin

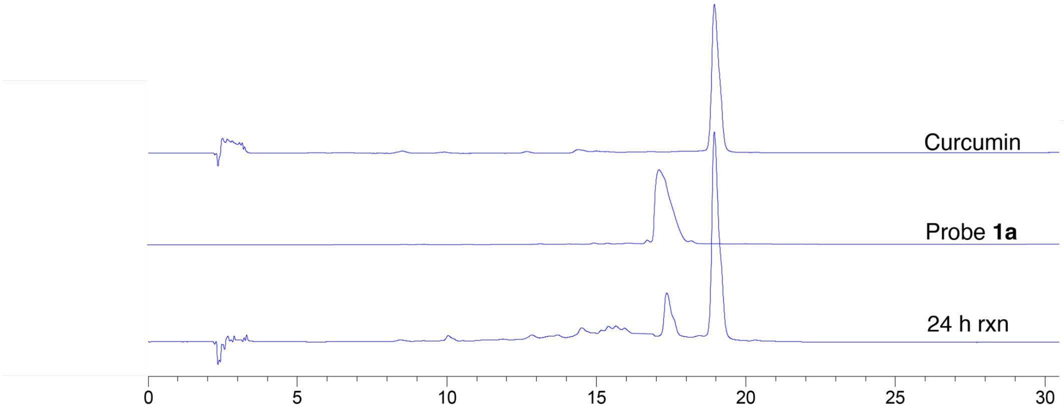

Probe 1a + hesperetin

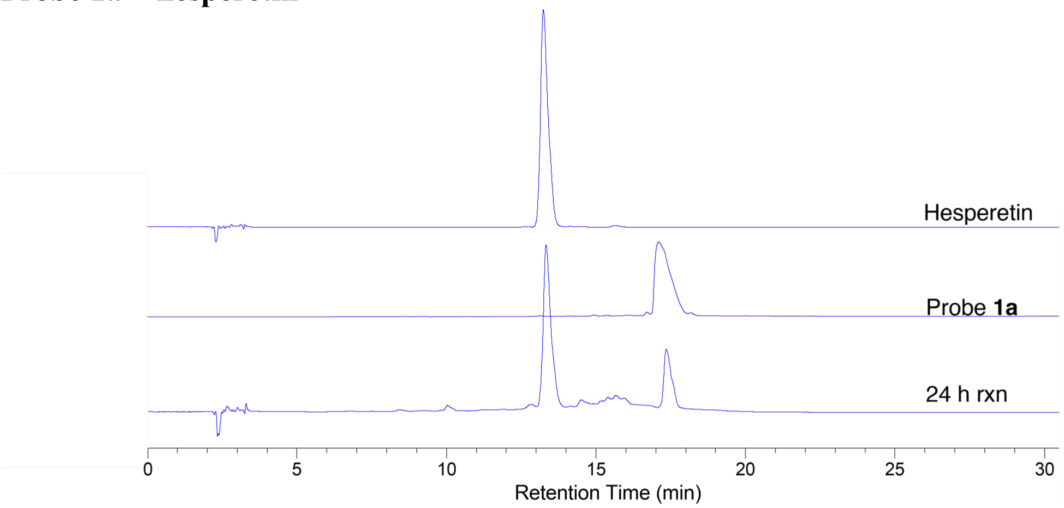

Probe 1a + resveratrol

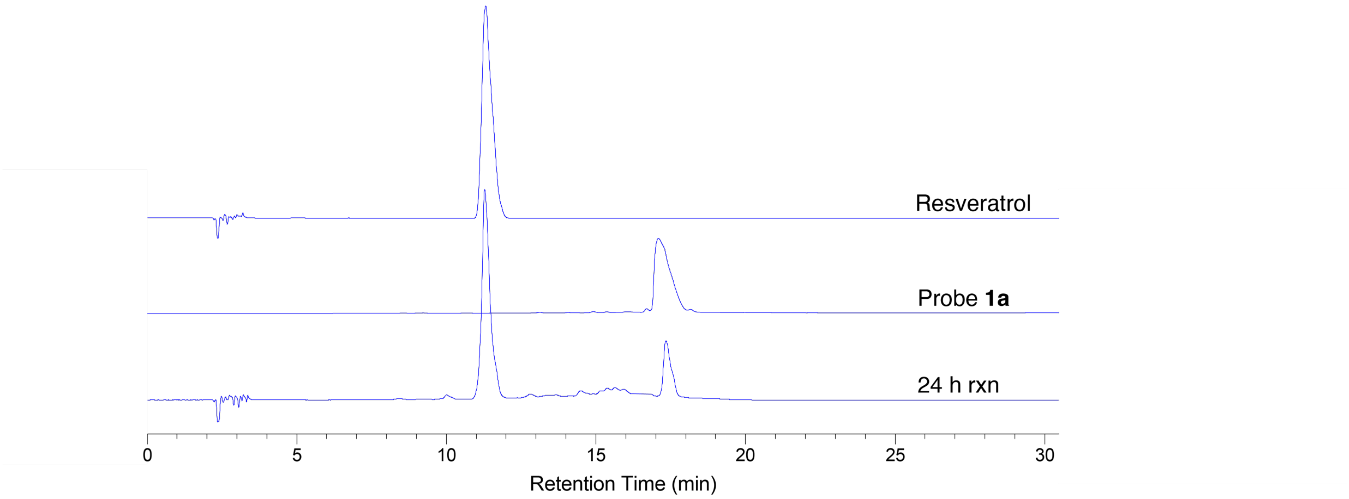

Fluorescence Spectra of Curcumin

Curcumin derivatives are a well-studied class of fluorophores. To alleviate potential concerns regarding the overlap of curcumin with probe **1a**, we analyzed curcumin's fluorescent spectra *in vitro* via a fluorimeter. 100 µM of curcumin in PBS (pH 7.4) was excited at 501 nm (excitation wavelength of probe **1a** used in imaging experiments). This matches literature spectra[7-10] of negligible fluorescence at ~500 nm excitation.

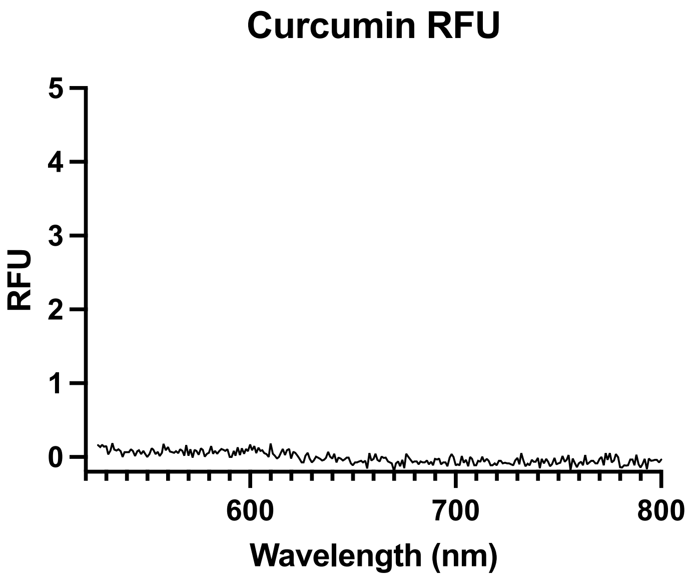

Impact of Curcumin on Probe Uptake

HeLa cells were plated in an IBIDI 8-well glass bottom chamber at a density of 25,000 cells per well in media and allowed to adhere overnight at 37 °C, 5% CO_2. A fresh 10 mM stock solution of probe **1a** was prepared in DMSO on the day of experimentation. Media was removed from wells and 200 μL of the final concentration in media with curcumin (10 μM) or curcumin (10 μM) and MGO (250 μM) was added to desired wells. Cells were placed in an incubator for 4 hours, followed by removal of the dosage media. Dosage media was removed, and 200 μL of 20 μM of probe **1a** in media was added to wells. Cells were incubated for 90 minutes. Probe media was removed, and cells were washed with 200 μL of PBS for 5 minutes in the incubator (repeated 3x). Cells were stained with Hoechst 33342 according to the manufacturer's protocol for 5 minutes. Staining media was removed, and cells were washed with 200 μL of PBS for 5 minutes (repeated 3 times). PBS was replaced with 200 μL of fresh media followed by immediate imaging. Five images were captured for each well. This process was repeated in triplicate on separate days with different cell passage numbers. 10 cells were analyzed per image to determine average ROI for each image. An outlier test was performed to remove any extraneous data points before statistical analysis was conducted via Student's T-test (n = 15). Data was normalized to cells treated with MGO alone. Error bars represent standard deviation.

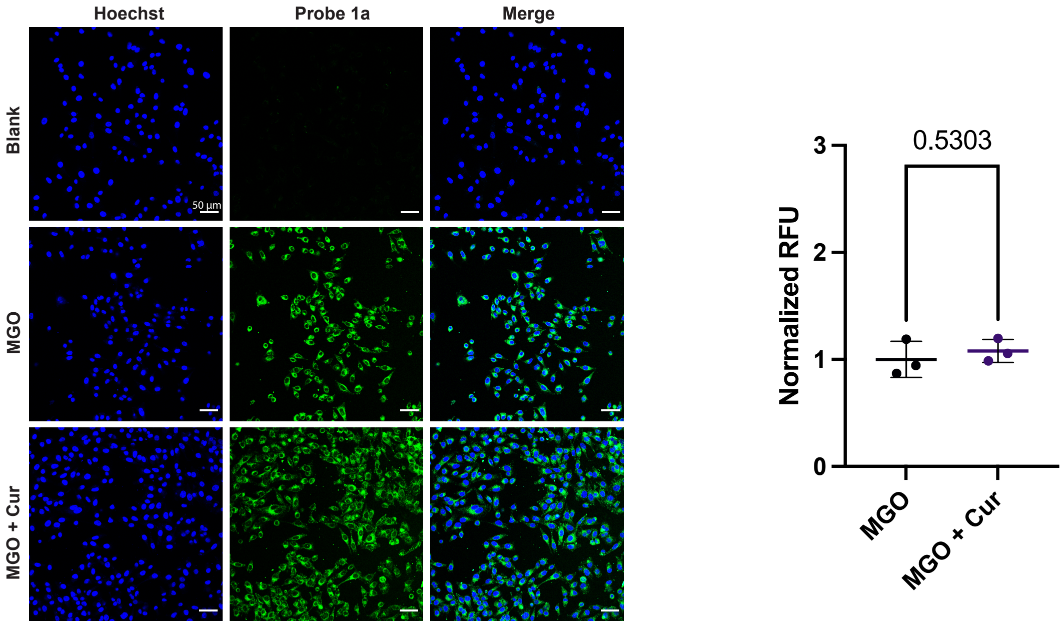

XV: Supplementary Figure S11: Reversible Monitoring of MGO in HeLa cells

HeLa ells were plated in an IBIDI 8-well glass bottom chamber at a density of 25,000 cells per well in media and allowed to adhere overnight at 37 °C, 5% CO_2. Media was removed from wells and 200 µL of resveratrol and hesperetin (Res and Hep, 10 µM and 50 µM) was added at t = -24 hours. At t = -1.5 hours, media was removed and 200 µL of 20 µM of probe **1a** in media was added to wells. Cells were incubated for 90 minutes. Media was removed and cells were washed 3 x 5 minutes with PBS. At t = 0 hours, media containing MGO (100 µM) was added to the wells, and again at t = 12 hours. Images were taken via confocal microscopy at the following time points: t = -5 minutes, t = 1 hour, t = 1.5 hours, t = 2 hours, t = 4 hours, t = 8 hours, t = 11 hours, t = 13 hours. Five images were captured for each well. 10 cells were analyzed per image to determine average ROI for each image. This process was repeated in triplicate on separate days with different cell passage numbers. An outlier test was performed to remove any extraneous data points before statistical analysis was conducted via Student's T-test (n = 15).

XVI: Supplementary Figure S12: Imaging of Murine Brain Tissue

Eight-week-old ICR-CD1 male mice (Charles River Laboratories) were injected with saline or MGO (200 µg or 400 µg per animal) in 10 µL volume by intracerebroventricular route (N=3/group) with the help of a hypodermic needle (27g x 1/4", Catalog # 4000, Cadence Science) attached to a Hamilton syringe under ketamine anesthesia. At 1-hour post injection, mice were sacrificed, and brain tissues were collected with a brief wash with ice-cold PBS. Samples were stored at -80 °C till the day of experimentation. Brains were transferred to 5 mL centrifuge tubes containing 10 µM probe **1a** in PBS (1.5 µL of 10 mM stock of probe **1a** in DMSO into 1498.5 µL of PBS) and allowed to diffuse for 16 hours in a 4 °C refrigerator. Solution was removed and brains were fixed with paraformaldehyde (PFA, 4% in H_2O) for 24 hours in a 4 °C refrigerator. PFA was removed and 1.5 mL of sucrose (30% w/v in H_2O) for 48 in a 4 °C refrigerator. Brains were then embedded in OT medium (Tissue-Tek) and sectioned by cryostat into 60 µM thick coronal sections at the level of the prefrontal cortex. Sections were immediately transferred to glass Superfrost Plus slides which were then cover slipped with Fluoromount-G (Southern Biotech, Birmingham, AL) and allowed to dry before imaging on a Leica Stellaris SP8 microscope. ROI values were calculated in FIJI ImageJ, checked via an outlier test, and analyzed by two-way Student's T-test.

XVII: References

[1]Kellum, M. W.; Oray, B.; Norton, S. J. A convenient quantitative synthesis of methylglyoxal for glyoxalase I assays. *Anal. Biochem.* **1978**, *85*, 586.

[2]Vince, R.; Daluge, S.; Wadd, W. B. Inhibition of glyoxalase I by S-substituted glutathiones. *J. Med. Chem.* **1971**, *14,* 402-404.

[3]Ueno, T.; Urano, Y.; Kojima, H.; Nagano, T. Mechanism-Based Molecular Design of Highly Selective Fluorescence Probes for Nitrative Stress. *J. Am. Chem. Soc.* **2006**, *128*, 10640.

[4] Xu, H.; Liu, Q.; Song, X.; Wang, C.; Wang, X.; Ma, S.; Wang, X.; Feng, Y.; Meng, X.; Liu, X.; Wang, W.; Lou, K. Fluorophore-Promoted Facile Deprotonation and Exocylic Five-Membered Ring Cyclization for Selective and Dynamic Tracking of Labile Glyoxals. *Anal. Chem*. **2020**, *92*, 13829-13838.

[5] Lo, T. W. C.; Westwood, M. E.; McLennon, A. C.; Selwood, T.; Thornalley, P. J. Binding and Modification of Proteins by Methylglyoxal under Physiological Conditions. **1994**, *269*, 32299.

[6] Stauffer, W.; Sheng, H.; Lin, H. N. EzColocalization: An ImageJ plugin for visualizing and measuring colocalization in cells and organisms. *Sci. Rep.* **2018**, *8*, 15764.

[7] Liu, Y.; Zhang, C.; Pan, H.; Li, L.; Yu, Y.; Liu, B. An insight into the *in vivo* imaging potential of curcumin analogues as fluorescence probes. *Asian J. Pharm. Sci.* **2021**, *16*, 419.

[8] Ferreira, J. M. R.; Alves, M.; Sousa, B.; Vieira, S. I.; Silva, A. M. S.; Guieu, S.; Cunha, A.; Nunes da Silva, R. Curcumin-based molecular probes for fluorescence imaging of fungi. *Org. Biomol. Chem.* **2023**, *21*, 1531.

[9] Saini, R. K.; Das, K. Photophysics of Curcumin excited state in toluene-polar solvent mixtures: Role of H-bonding properties of the polar solvent. *J. Lumin.* **2014**, *145*, 832.

[10] Ghosh, M.; Sarkar, N. Exploring the World of Curcumin: Photophysics, Photochemistry, and Applications in Nanoscience and Biology. *ChemBioChem* **2024**, *25*, e202400335.

XVIII: Characterization of synthesized compounds.

^{1}H NMR spectra of 2 in D_2O

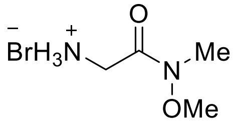

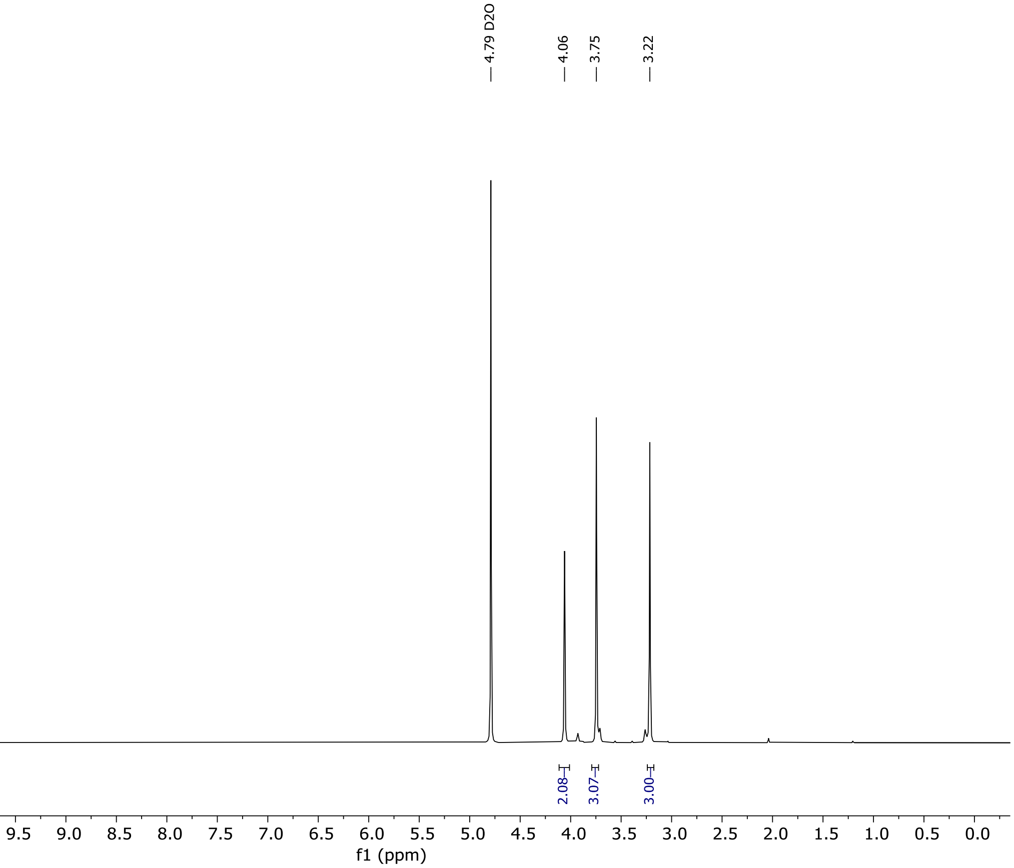

^{1}H NMR spectra of 3 in $CDCl_3$

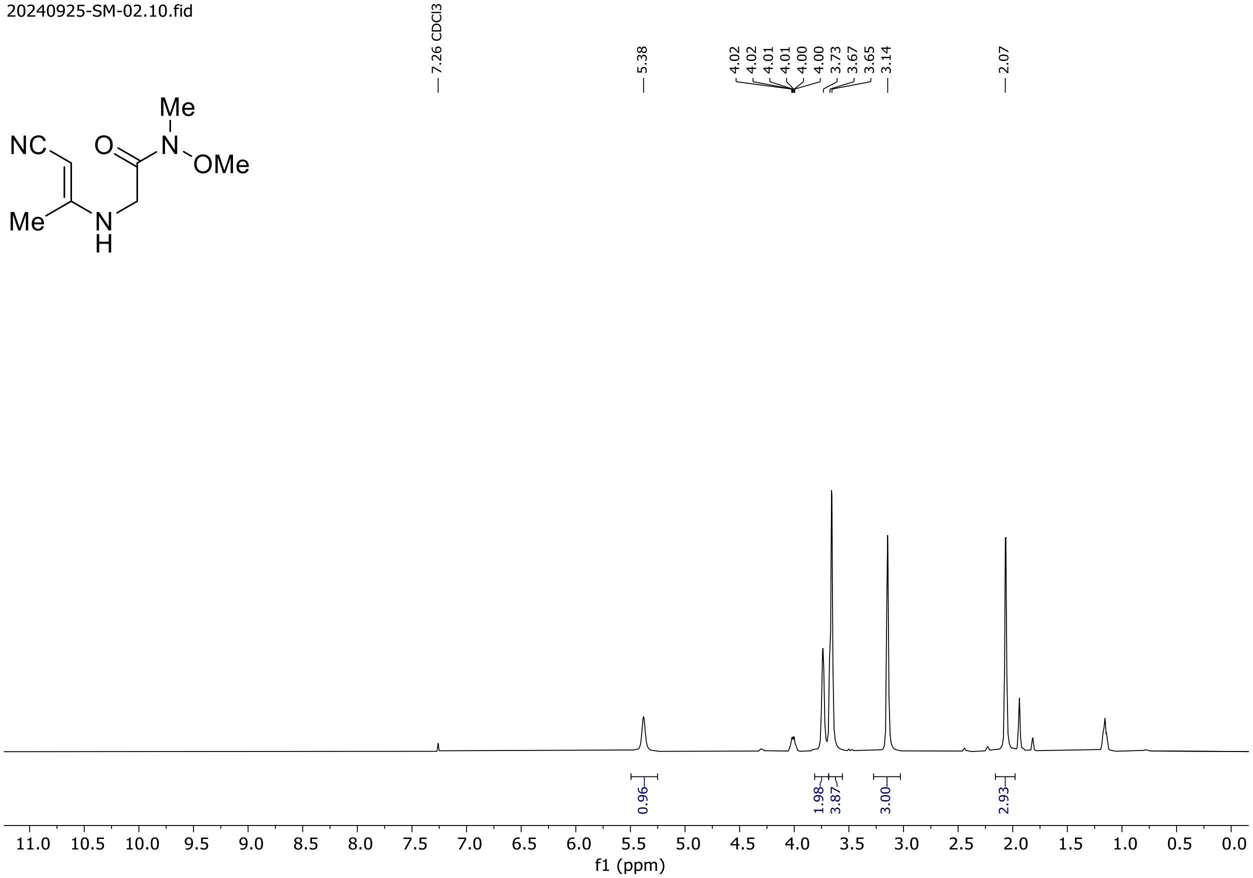

^{1}H NMR spectra of 4 in $CDCl_3$

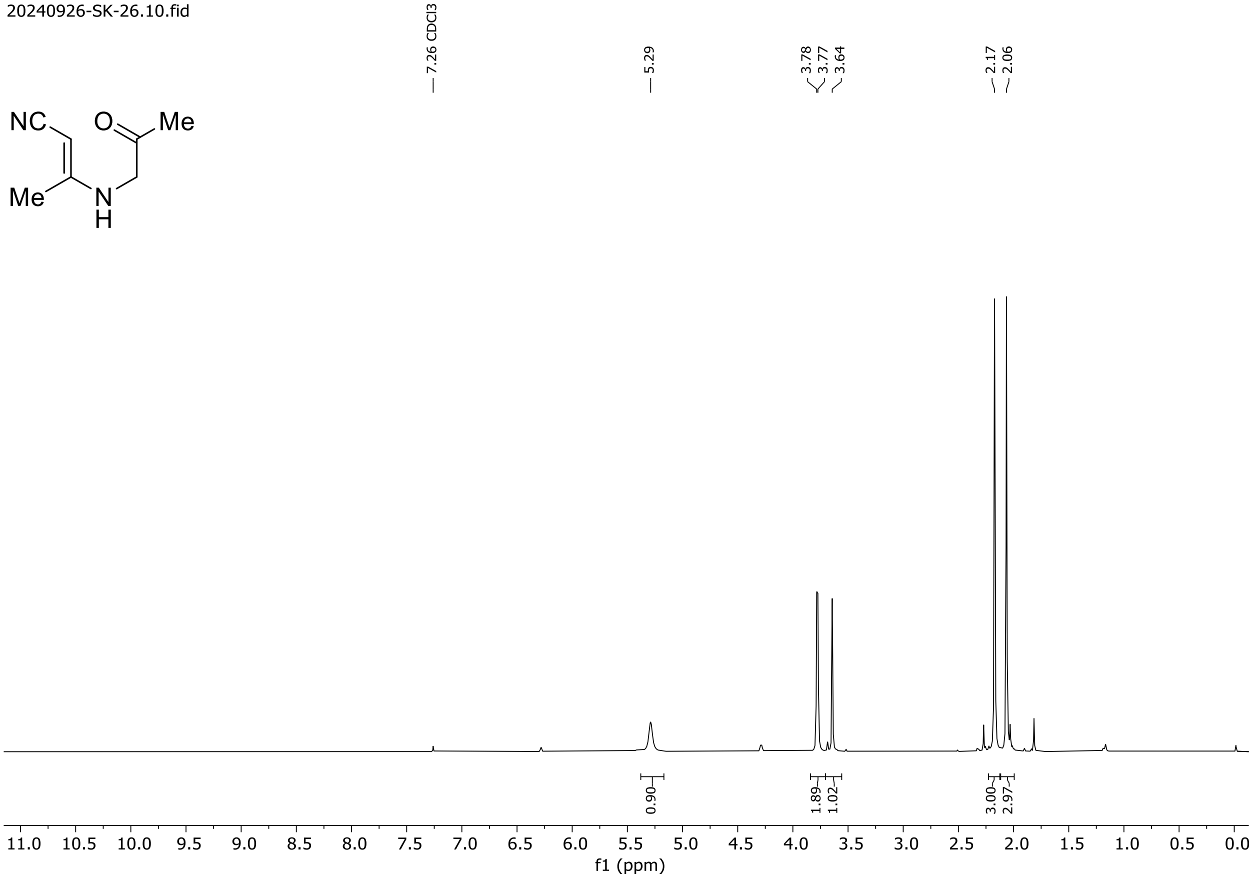

^{1}H NMR spectra of 5 in $CDCl_3$

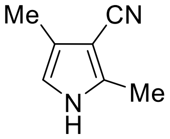

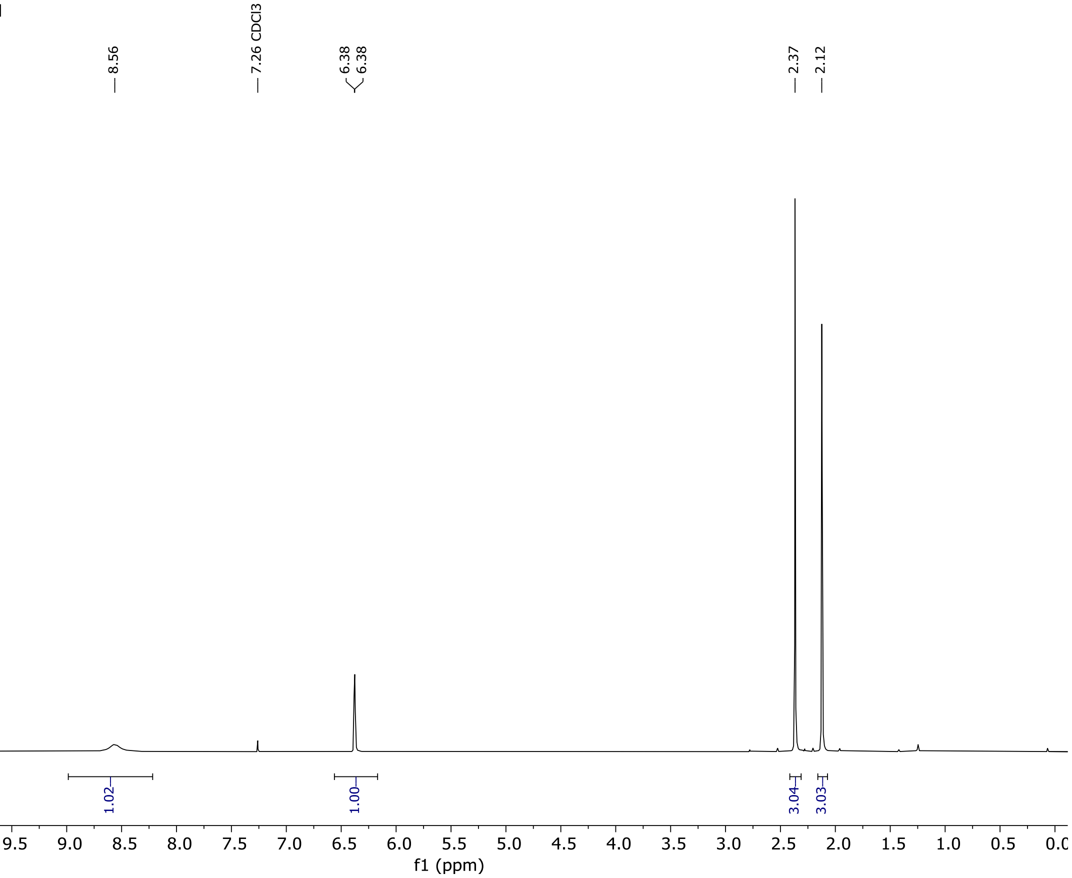

^{13}C NMR spectra of 5 in $CDCl_3$

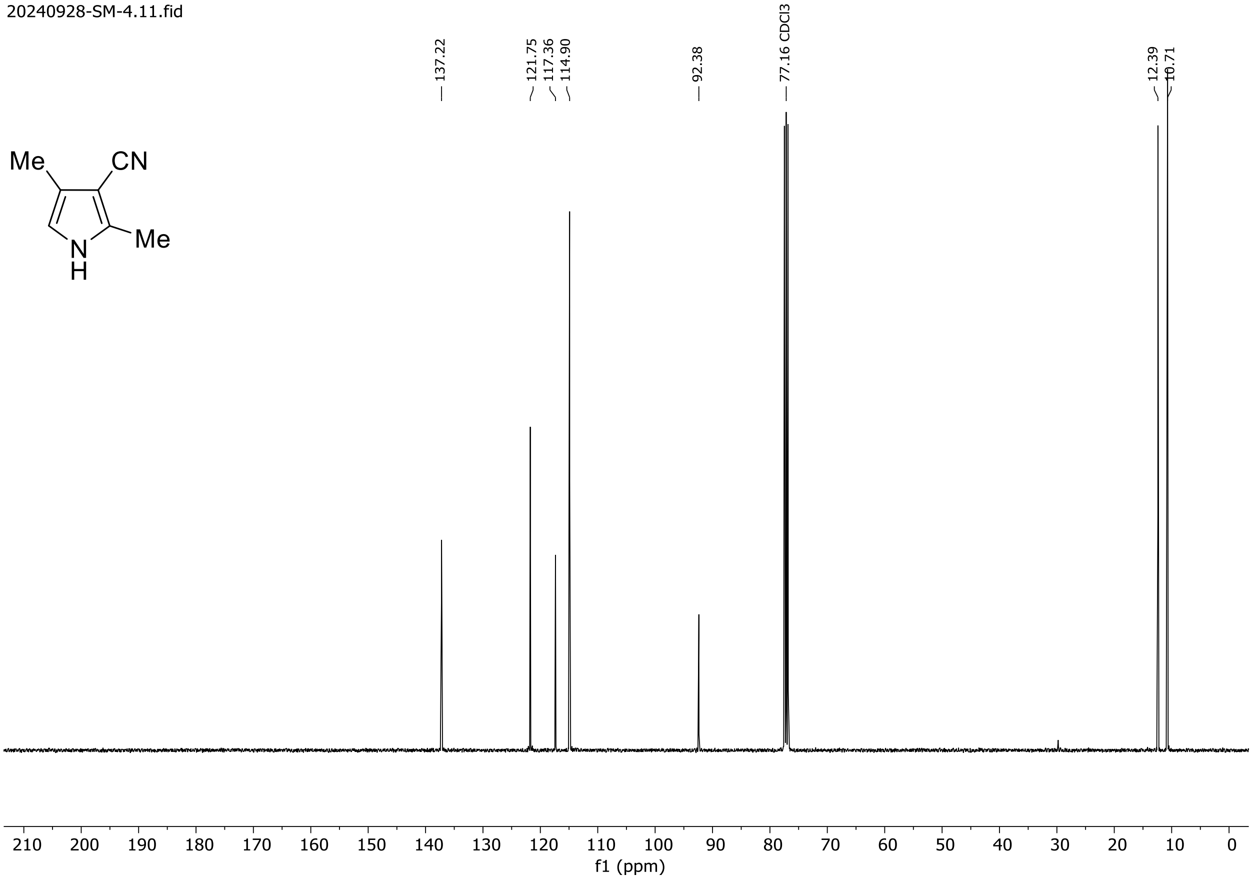

^{1}H NMR spectra of 6 in DMSO-d_6

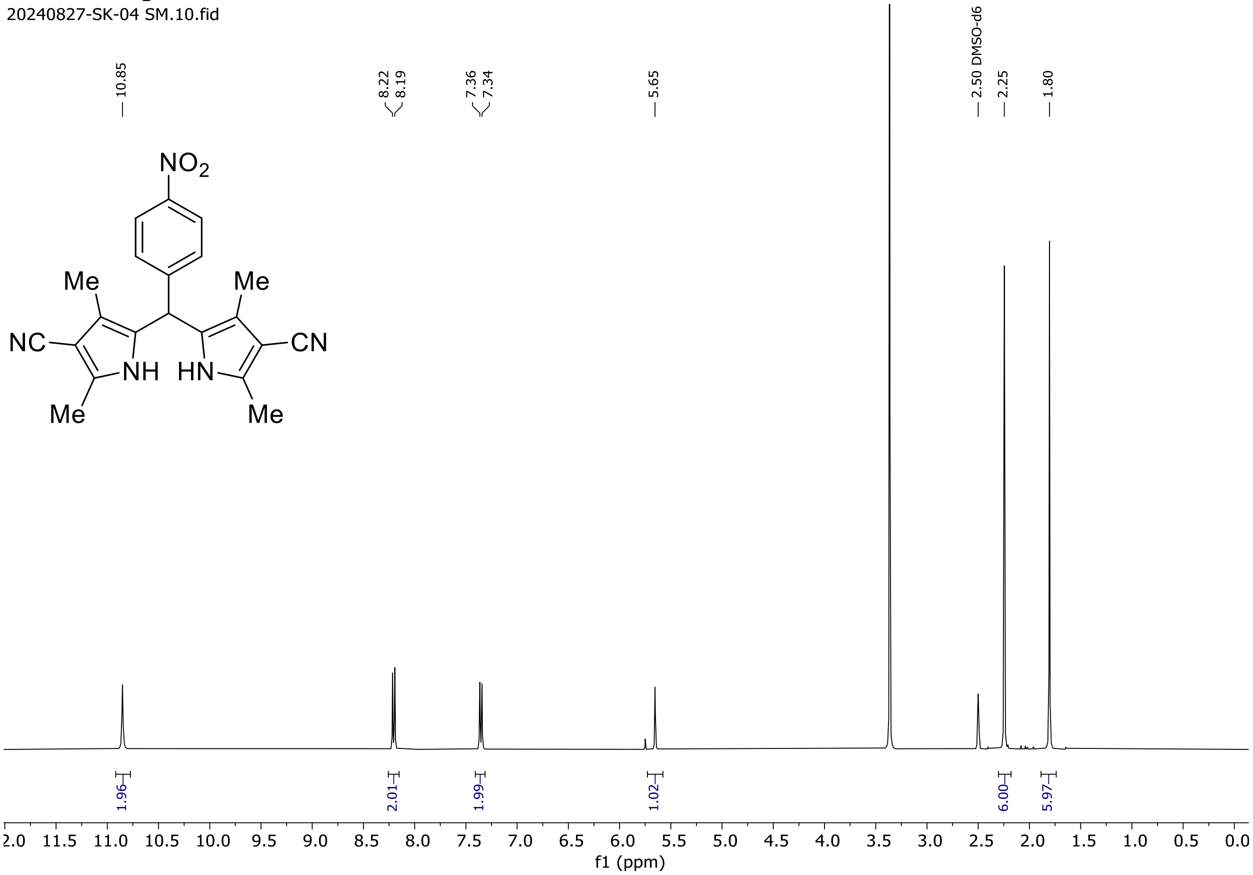

^{1}H NMR spectra of 7 in DMSO-d_6

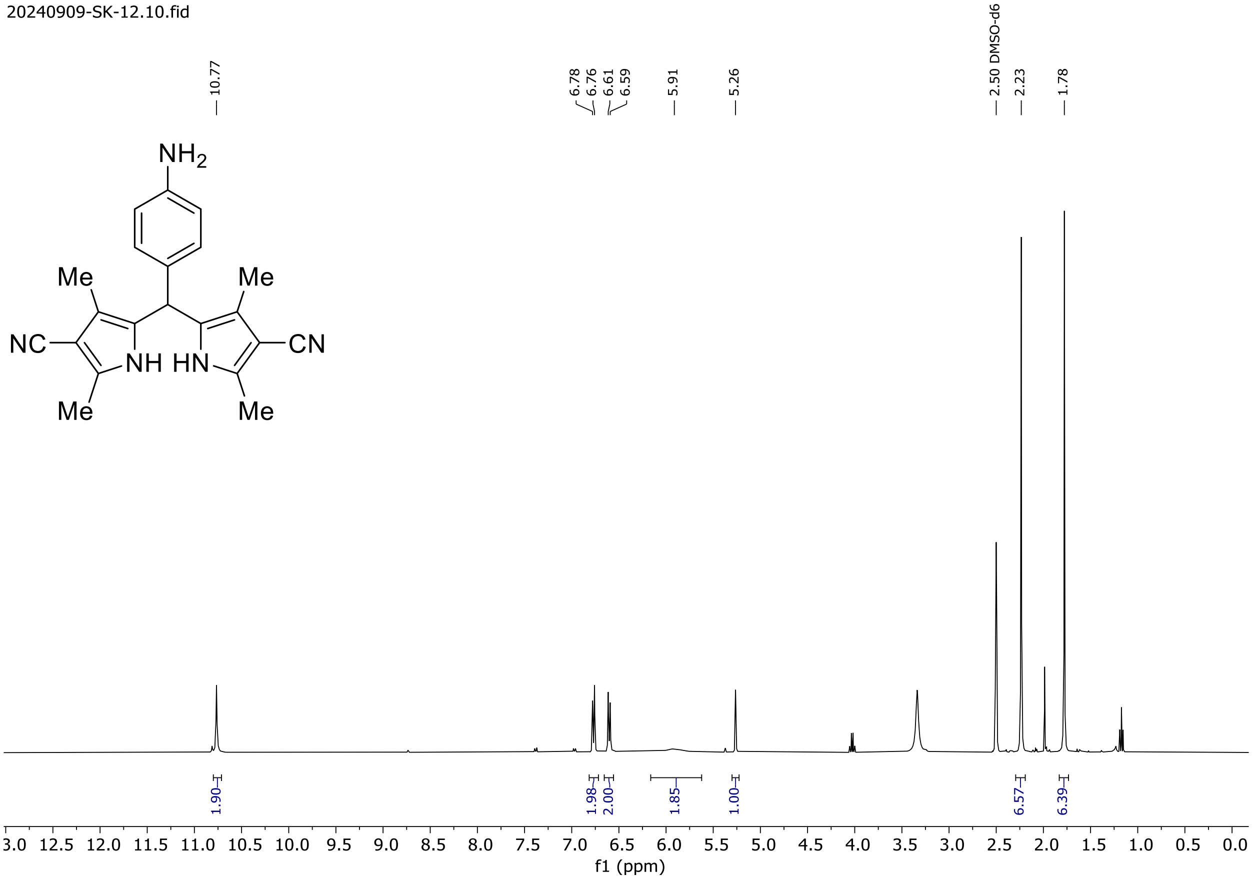

^{1}H NMR spectra of 8 in DMSO-d_6

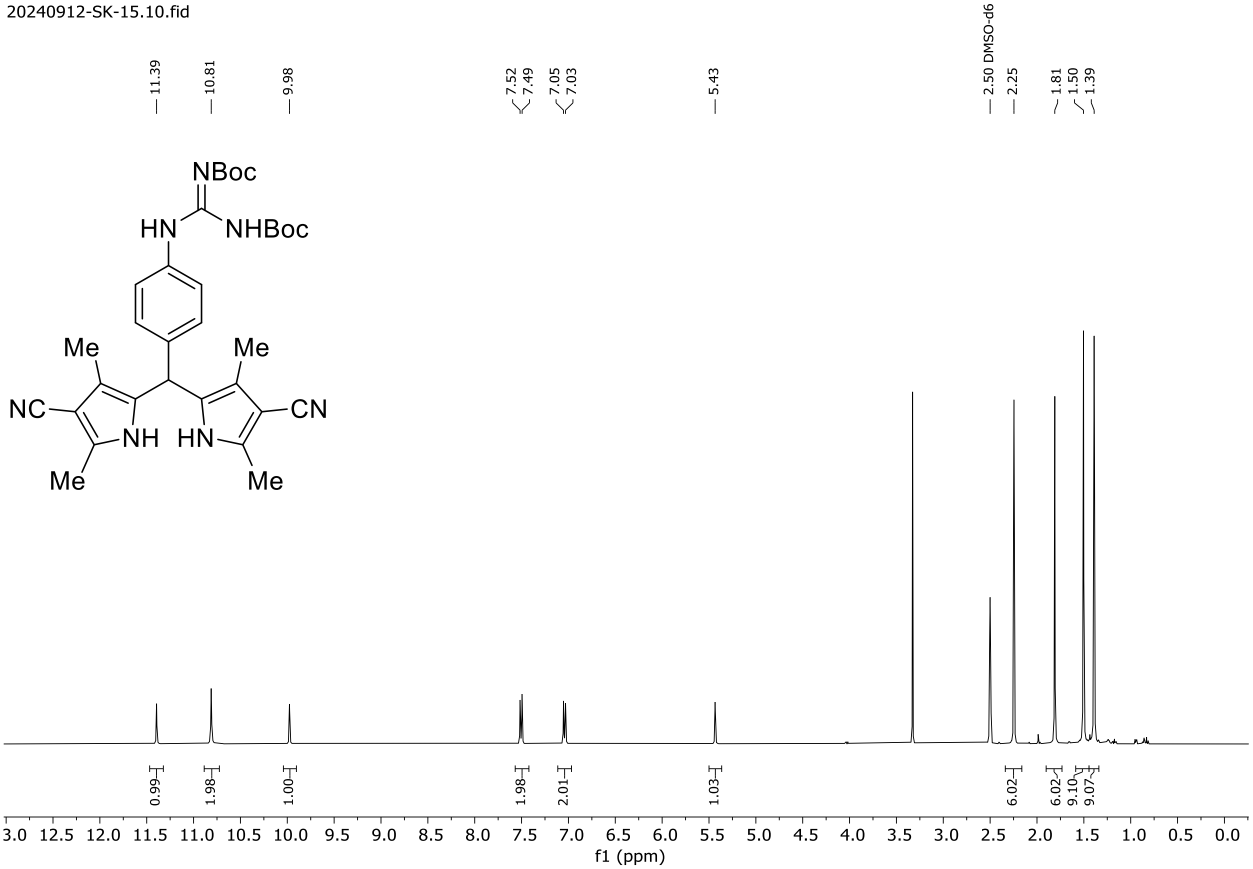

^{13}C NMR spectra of 8 in DMSO-d_6

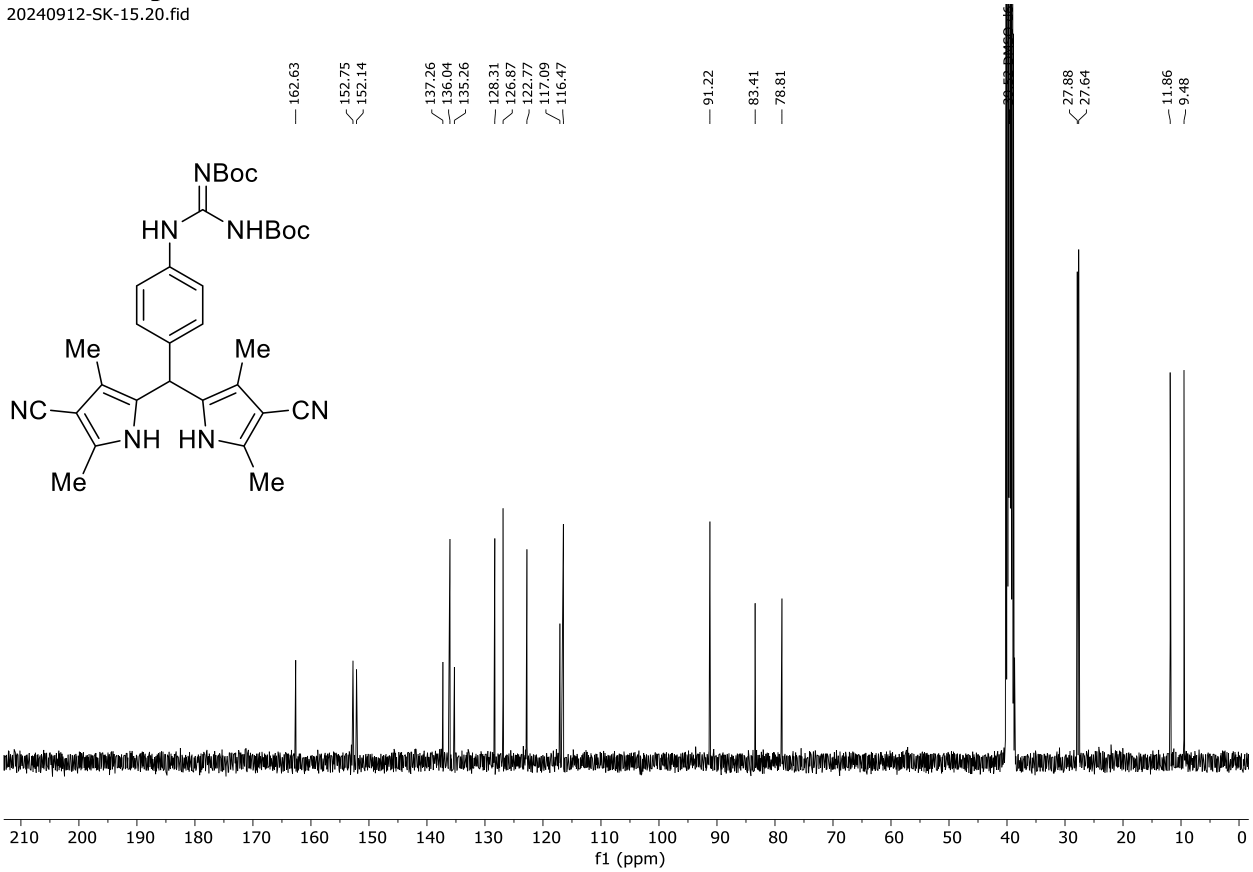

^{1}H NMR spectra of probe 1a in DMSO-d_6

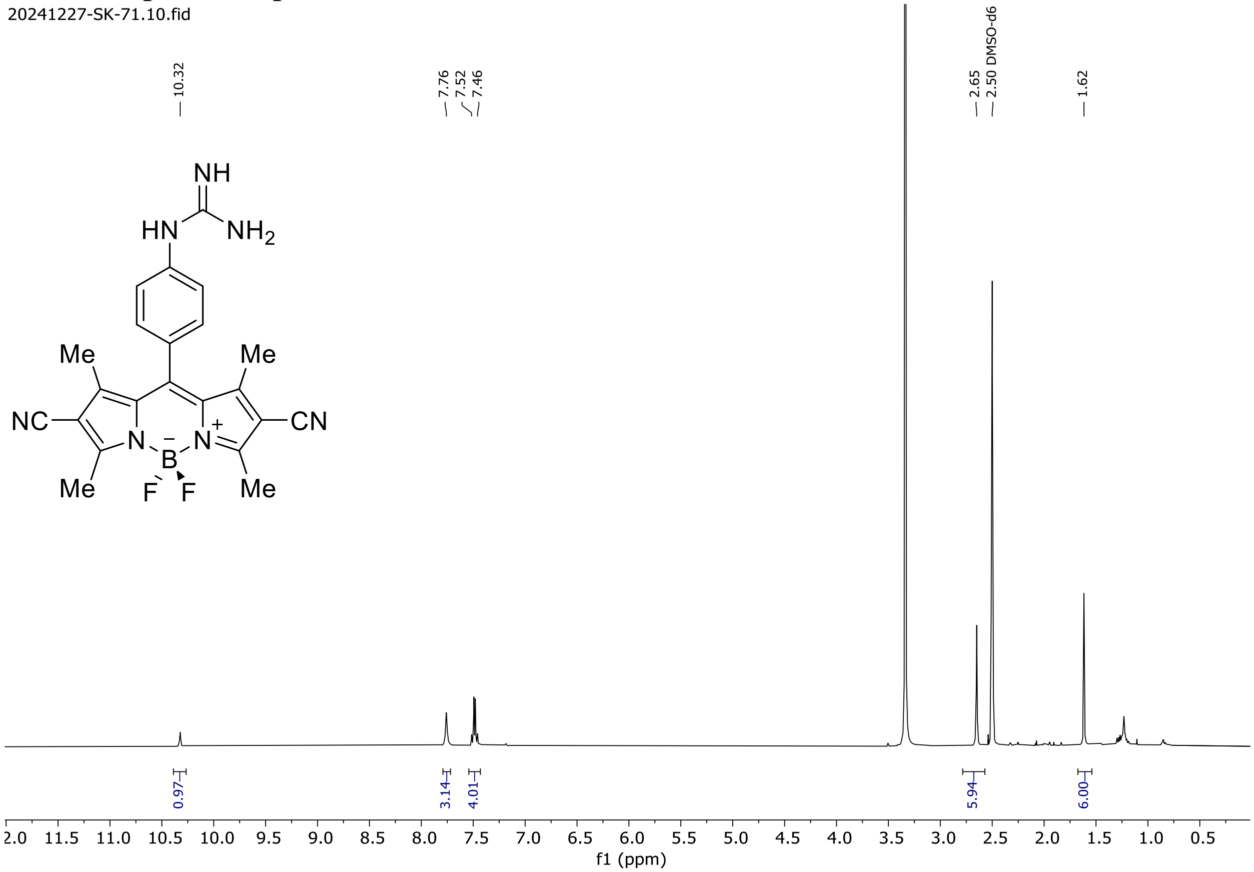

^{13}C NMR spectra of probe 1a in DMSO-d_6

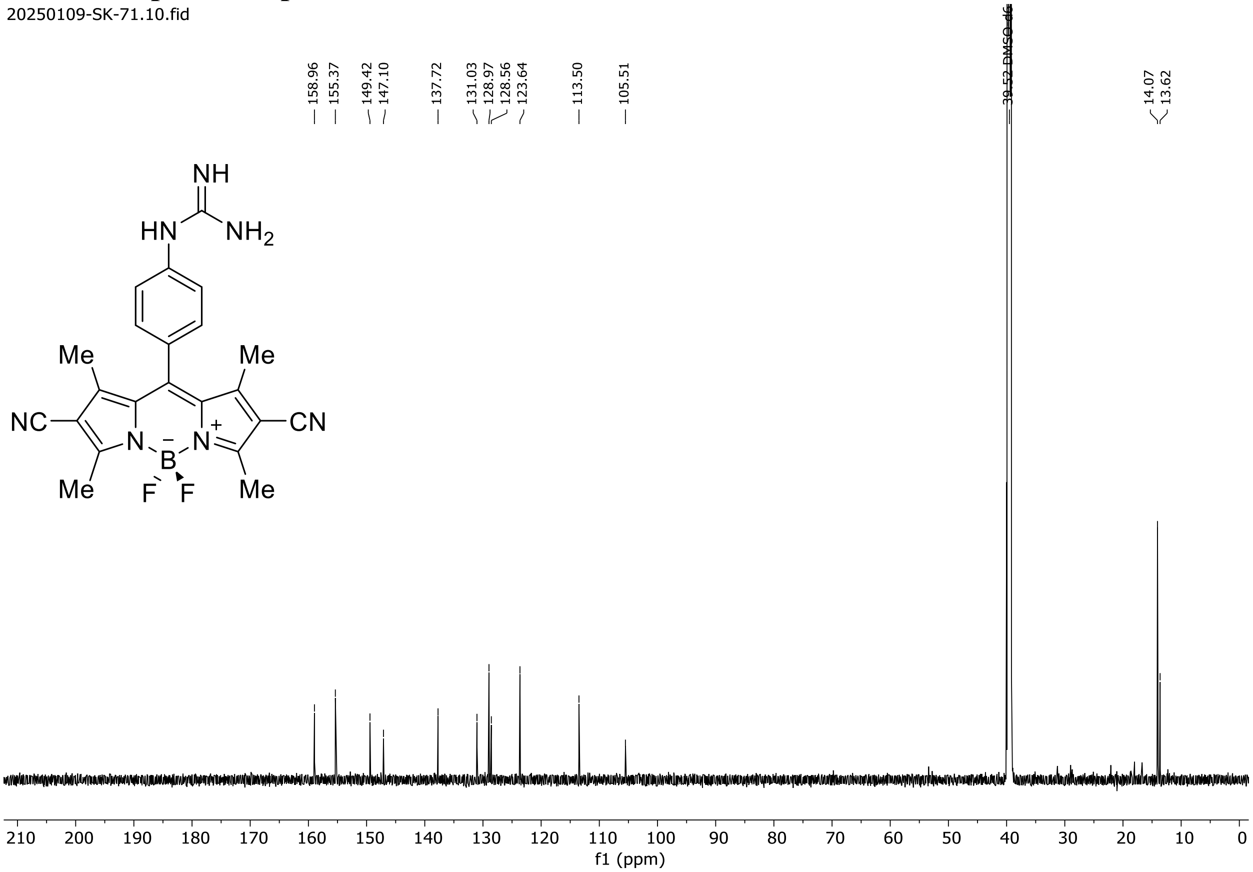

DEPT-135 NMR spectra of probe 1a in DMSO-d_6

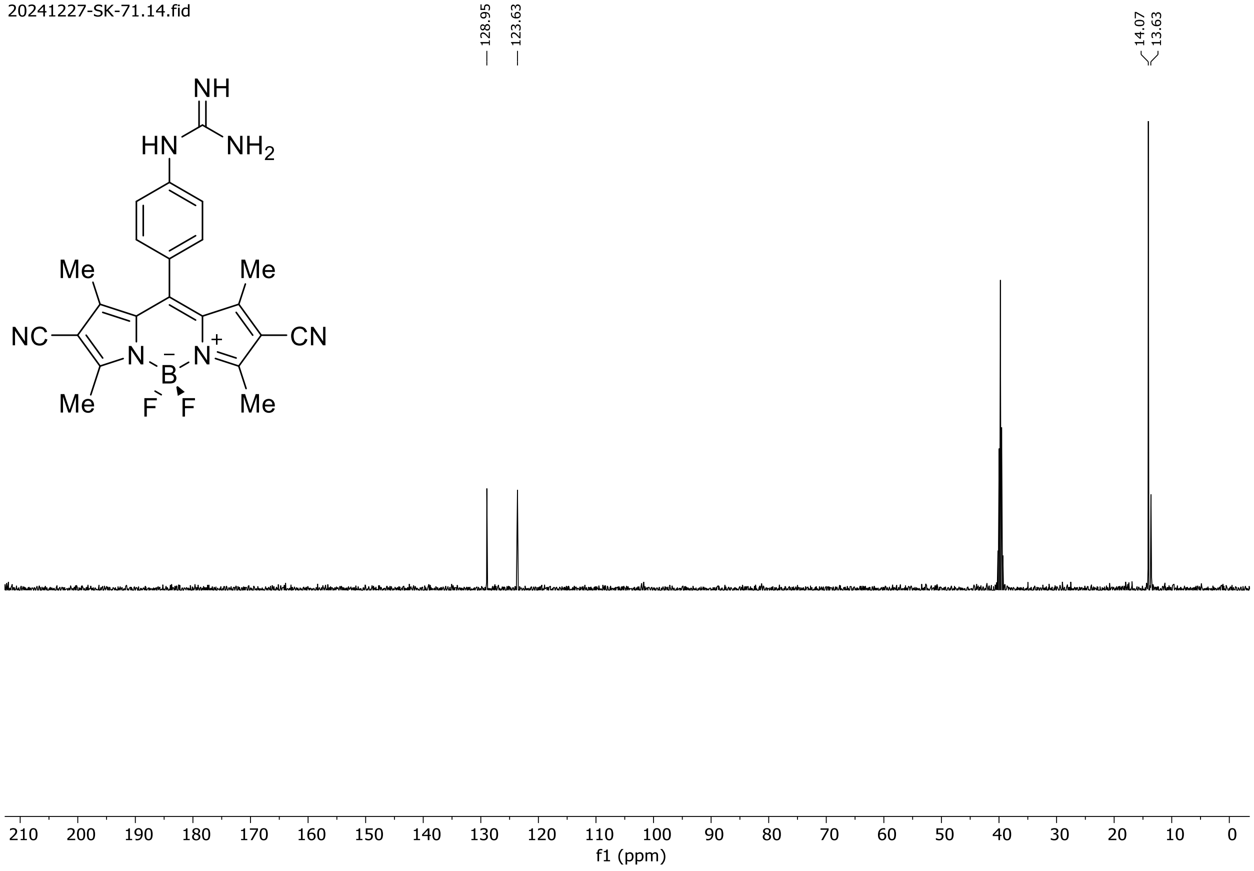

^{11}B NMR spectra of probe 1a in DMSO-d_6

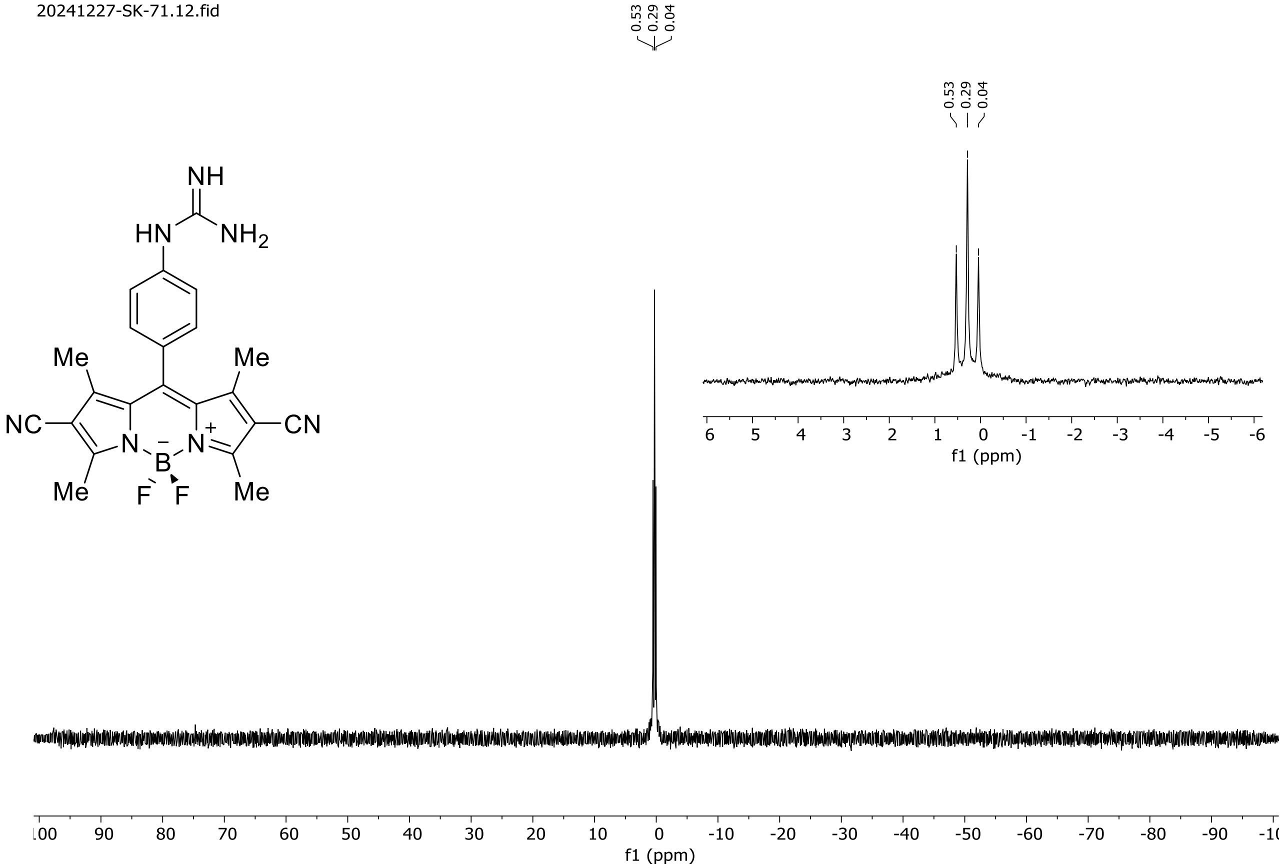

^{19}F NMR spectra of probe 1a in DMSO-d_6

20241227-SK-71.11.fid

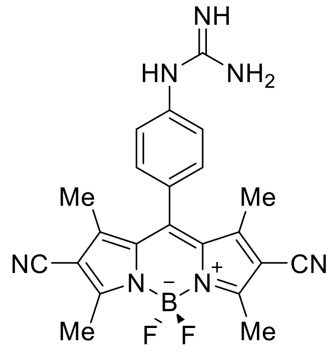

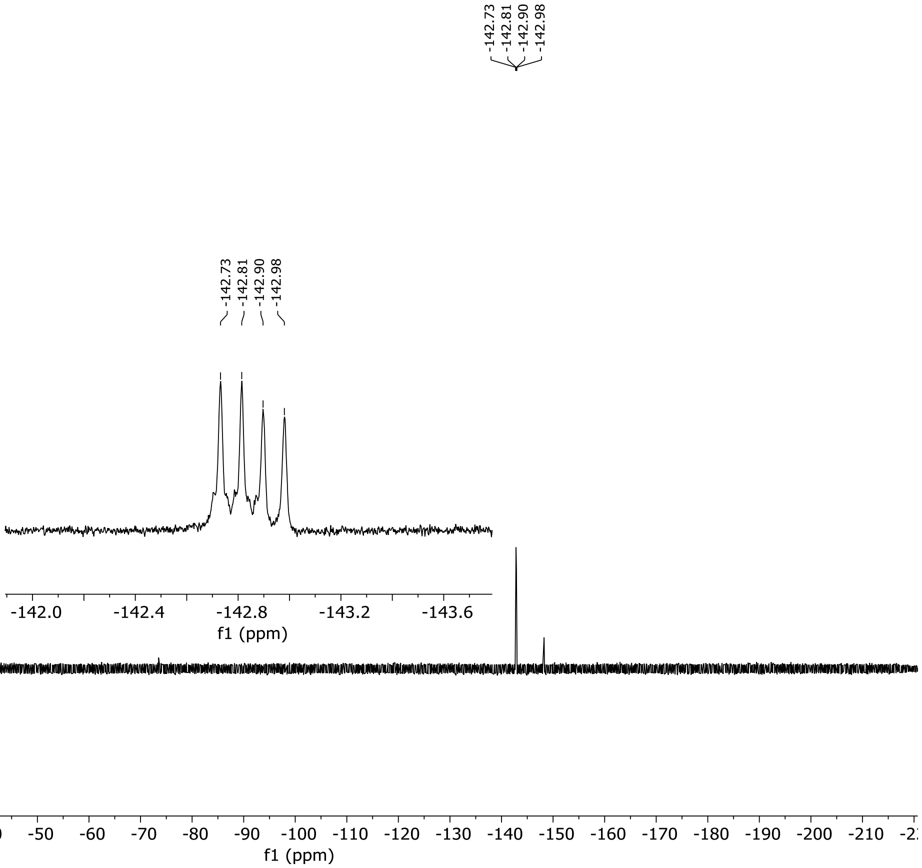

Supplement: Supplementary file 1 [file au6c00566_si_001.pdf]
